# *Salvia chinensia* Benth induces autophagy in esophageal cancer cells via AMPK/ULK1 signaling pathway

**DOI:** 10.3389/fphar.2022.995344

**Published:** 2022-09-02

**Authors:** Lei Jia, Xin-Rong Lin, Wen-Yan Guo, Ming Huang, Yang Zhao, Yu-Shuang Zhang, Jing Li

**Affiliations:** ^1^ College of Integrated Chinese and Western Medicine, Hebei Medical University, Shijiazhuang, Hebei, China; ^2^ Department of Traditional Chinese Medicine, The Third Hospital of Hebei Medical University, Shijiazhuang, Hebei, China; ^3^ Department of Traditional Chinese Medicine, Tumor Hospital of Hebei Province, The Fourth Hospital of Hebei Medical University, Shijiazhuang, Hebei, China

**Keywords:** Salvia chinensia Benth, herbal medicine, esophageal cancer, autophagy, proteomics, AMPK/ULK1 pathway, SensGFP-StubRFP-LC3

## Abstract

*Salvia chinensia* Benth (Shijianchuan in Chinese, SJC) has been used as a traditional anti-cancer herb. SJC showed good anti-esophageal cancer efficacy based on our clinical application. However, the current research on SJC is minimal, and its anti-cancer effect lacks scientific certification. This study aims to clarify the inhibitory effect of SJC on esophageal cancer and explore its underlying mechanism. Q-Orbitrap high-resolution LC/MS was used to identify the primary chemical constituents in SJC. Cell proliferation and colony formation assays showed that SJC could effectively inhibit the growth of esophageal tumor cells *in vitro*. To clarify its mechanism of action, proteomic and bioinformatic analyses were carried out by combining tandem mass labeling and two-dimensional liquid chromatography-mass spectrometry (LC-MS). Data are available *via* ProteomeXchange with identifier PXD035823. The results indicated that SJC could activate AMPK signaling pathway and effectively promote autophagy in esophageal cancer cells. Therefore, we further used western blotting to confirm that SJC activated autophagy in esophageal cancer cells through the AMPK/ULK1 signaling pathway. The results showed that P-AMPK and P-ULK1 were significantly up-regulated after the treatment with SJC. The ratio of autophagosomes marker proteins LC3II/I was significantly increased. In addition, the expression of the autophagy substrate protein P62 decreased with the degradation of autophagosomes. Using lentiviral transfection of fluorescent label SensGFP-StubRFP-LC3 protein and revalidation of LC3 expression before and after administration by laser confocal microscopy. Compared with the control group, the fluorescence expression of the SJC group was significantly enhanced, indicating that it promoted autophagy in esophageal cancer cells. Cell morphology and the formation of autophagosomes were observed by transmission electron microscopy. Our study shows that the tumor suppressor effect of SJC is related to promoting autophagy in esophageal tumor cells *via* the AMPK/ULK1 signaling pathway.

## Introduction

According to the global cancer incidence, mortality and prevalence report, esophageal cancer is the top 10 common type and cause of cancer-related death worldwide (Bray et al., 2018). In 2020, an iARC -- Cancer Today survey showed that 47.42% of esophageal cancer in the world occurred in China (Zhou et al., 2022). Hebei Province, specifically Cixian County and Shexian County located to the south of the Taihang Mountains, has particularly high mortality and morbidity of esophageal cancer when compared to the rest of China and the world (He et al., 2015; Yuan et al., 2019). Data have shown that people over 60 will mainly be affected (Bollschweiler et al., 2017). Esophageal carcinoma (EC) is divided into esophageal adenocarcinoma (EAC) and esophageal squamous cell carcinoma (ESCC). In Asia, especially China, ESCC is the most common histological type, accounting for more than 90% of esophageal cancers (Sakai et al., 2022). Smoking and drinking are the main factors leading to the high incidence of esophageal cancer. In Asia, frequent drinking of extremely hot drinks and eating pickled vegetables are also high-risk factors (Ghosh and Jones, 2022). Conventional treatment for esophageal cancer is not effective and the 5-year survival rate of patients is only about 20% (Chen et al., 2019). Therefore, there is an urgent need to find new treatments to improve patient survival.

In the treatment of cancer, Chinese herbal medicines have obvious benefits: they support patient immunity, alleviate adverse effects of radiotherapy and chemotherapy, prevent recurrence and metastasis, improve patient quality of life, and prolong the survival rate (Zhang F et al., 2022). Many Chinese herbal monomers and their compounds have been found to have anticancer activities and inhibit esophageal cancer (Wu et al., 2021). *Salvia chinensia* Benth (Shijianchuan in Chinese, SJC) is the whole grass of purple ginseng of Salvia genus in the Libidaceae family. It first appeared in the ancient Chinese medicine book Compendium of Materia Medica with the following functions: promotes blood circulation, removes stasis, reduces the mass and disperses knots. The Dictionary of Chinese Herbal Medicine records that SJC treats “Yege” (Tao, 2018), which is a traditional Chinese medicine term, manifested as dysphagia, or pain in swallowing, hard swallowing, or vomiting after eating, including esophageal cancer in modern medicine (Jiali, 2021). In clinical practice, we found that SJC has a significant effect on the treatment of esophageal cancer, so it is worth further study as a potential therapeutic drug. In this study, we made proteomic characterization of the effect of high efficiency mass spectrometry on the effect of SJC on esophageal cancer and explore its anticancer mechanism.

## Materials and methods

### Instruments

Cell incubator (Becton Dickinson and Company, United States); Automatic microplate tester (Bio Tek); Nanoscale ULTRA-high Performance Liquid Chromatograph (Thermo, United States); Orbitrap Fusion High Resolution Mass Spectrometer (Thermo, United States); Ultimate 3000 Ultra Rapid Liquid Chromatograph (Thermo Corporation, United States); Confocal Quantitative Image Cytometer (YOKOGAWA, Japan); ChemiDoc MP System All-in-one Imaging System (Bio-RAD Corporation, United States).

### Drugs and reagents

Experimental esophageal cancer cell lines KYSE-150 and TE-1 were purchased from the Cell Resource Center of Shanghai Institutes of Biological Sciences, Chinese Academy of Sciences. Phosphate buffered saline (PBS) (BI, Beit Haemek, Israel), RPMI 1640 cell culture medium (Corning, United States), newborn calf serum (NCS), Trypsin-EDTA solution (Solarbio, China), Trypsin -without EDTA Solution (Solarbio, China), Fetal calf serum (FCS) (BI, Beit Haemek, Israel), experimental dimethyl sulfoxide (DMSO) (Sigma, United States), Shi Jian Chuan granular (Jiangyin Pharmaceutical, China, batch number:19092701), and a Cell Counting Kit -8 (CCK-8 kit) (Tongren Corporation, Japan) were used.

### Preparation of the drug

SJC (60 mg) was dissolved in 50 ml RPMI 1640 medium and placed on a heated magnetic stir plate, then heated to 45°C for 30 min. After stirring for 30 min, impurities and bacteria were filtered through a sterile 0.22 μm filter membrane, and FCS was added in a ratio of 9:1 to prepare the mother liquor, which could be further diluted for experiments.

### Identification of chemical constituents of SJC by Q-OrbitRAP high resolution liquid mass spectrometry

After grinding SJC for 5 min, 1 ml 80% methanol solution and grinding beads were added, and the mixture was vortexed for 10 min. The supernatant was centrifuged at 4°C for 10 min with a centrifugal force of 20,000 × *g*. After filtration, the sample was analyzed using qEXactive high resolution mass spectrometer.

### Cell viability detection

According to the manufacturer’s instructions, CCK-8 is used to measure the ability of cells to proliferate. KYSE-150 cells were inoculated into 96-well plates at 2000 cells/well at the logarithmic growth stage. TE-1 cells were inoculated into 96-well plates at a density of 3000 cells/well, and the SJC concentrations were 0, 100, 200, 400, 800, 1600, 3200, and 4000 μg/ml. After 100 μl different concentrations of SJC culture solution treatment for 24, 48, 72 h, we added 10 μl CCK-8 solution to each well and incubated for 2 h. The cell survival rate was calculated by measuring the OD value at 450 nm with a microplate reader. Cell survival rate = [(experimental group minus blank group)/(control group minus blank group)] × 100%.

### Plate colony formation method

At the logarithmic growth stage, KYSE-150 cells stage was inoculated in 6-well plates at a density of 2000 cells/well and TE-1 cells of 1000 cells/well. Both kind of cell lines were cultured in 1.5 ml medium in each well for 24 h at 37°C with 5% CO_2_. Cells were attached to the plates, the medium was discarded, and normal culture media were successively added [62.5 μg/ml, 125 μg/ml, 250 μg/ml, 500 μg/ml of SJC] every other day. After 10 days of culture, the medium was discarded, cells were washed once with PBS, fixed with methanol for 10 min, washed with PBS once again, and incubated with 1 ml Giemsa dye solution for 20 min. The wells were rinsed with PBS once, and cells were then photographed.

### Proteomic analysis

KYSE-150 cells in the logarithmic growth stage were inoculated into a T25 culture flask at a density of 1 × 10^6^ cells per bottle and incubated at 37°C for 24 h with 5% CO_2_. After the cells adhered to the flask, the medium was discarded, and normal medium, 250 and 500 μg/ml of SJC were successively added. Cell proteins were extracted after 48 h culture. After determining the cell protein concentration by the BCA method, 50 µg protein extract was removed for each group, and 100 mM DTT and 500 mM IAM were added for reductive alkylation. The samples of each group were combined with glacial acetone and placed in the refrigerator at −20°C overnight to precipitate proteins. Lys-c enzyme and trypsin were successively added to the precipitated proteins for enzyme digestion. A tandem mass tag (TMT) labeling kit was used for isotope labeling of peptide samples in the control group, the low concentration group, and the high concentration group. The mixed labeled peptides were fractionated on a BEH C18 column using the Dionex Ultimate 3000 RSLC system. The fractions were determined by ULTRA-high performance liquid chromatography easy-NLC1000 tandem Orbitrap Fusion high resolution mass spectrometer. The high energy collisional dissociation (HCD) of three parent ions with the strongest signal were selected from the first-order spectrum, and the obtained data were retrieved and analyzed by Proteome Discoverer 2.1. The mass spectrometry proteomics data have been deposited to the ProteomeXchange Consortium *via* the PRIDE [1] partner repository with the dataset identifier PXD035823.

### Bioinformatics analysis

All proteins retrieved from the database were analyzed, and differential proteins were screened from proteins with altered expression. The fold changes greater than 1.2 were defined as up-regulated and less than 0.83 were down-regulated. The differential proteins obtained by screening were searched in the DAVID database. Omic Share Tools was used to analyze the protein interaction of the differential proteins and their functions were classified and analyzed.

### Western blotting detected the AMPK/ULK1 signaling pathway and the autophagy proteins

KYSE-150 cells treated with 0, 250 and 500 μg/ml of SJC were splitting and centrifuged at 4°C and 12,000 rpm, then quantified the collected supernatant. Each pretreated sample was subjected to polyacrylamide gel protein electrophoresis and transferred to PVDF membrane, which was blocked with 5% BSA at room temperature for 1 h, washed three times with PBS, then incubated with primary antibody and secondary antibody successively. The main antibodies used were P-AMPK α (CST, United States), AMPKα (CST reagent, United States), P-ULK1 (CST, United States), LC3-II/I (CST, United States), P62 (CST, United States), and β-actin (Thermo, United States). The antibody was diluted in the ratio of 1:1000. The secondary antibody was HRP labeled goat anti-rabbit/mouse IgG. ImageJ was used for quantitative analysis, and independent experiments were repeated three times.

### Laser confocal detection of autophagic proteins LC3

24 h after the detection of lentivirus by autophagy of cells with overexpression of sensGFP-StubRFP-LC3, puromycin at the appropriate concentration was added to screen stable expression cells, and 1 × 10^4^ KYSE-150 cells were placed in 96-well plates, and cells were attached to the bottom. Cells were treated with combinations of rapamycin or chloroquine and SJC for 48 h. The nuclei were stained with Hoescht and immediately placed in a confocal microscope for photography and data analysis.

### Transmission electron microscopy observation

Human esophageal cancer KYSE-150 cells at the logarithmic growth stage were inoculated into culture flasks with 1 × 10^5^ cells/ml. Cell suspension (6 ml) was added into each bottle and cultured for 24 h at 37°C in 5% CO_2_. Then, one group was supplemented with a normal medium and the other with 500 μg/ml of SJC medium, three bottles per group, cultured for 48 h, and collected the cells by centrifugation at 1000 rpm for 10 min. Next, transferred to a 1.5 ml Eppendorf tube, washed twice with pre-cooled PBS, fixed with 2.5% glutaraldehyde for 24 h, wrapped with 1% agarose, and rinsed with 0.1 M phosphoric acid buffer PB (pH 7.4) three times. 1% osmic acid was added for 2 h. After gradient dehydration with acetone, the cells were embedded with an epoxy resin embedding agent and made into ultra-thin sections. Then the cells were saturated with 2% uranium acetate and stained with lead citrate. The autophagy of the cells was observed by transmission electron microscopy and then photographed.

### Statistical analysis

We used SPSS 25.0 and GraphPad Prism 8.0 for statistical analysis. If the data conformed to normal distribution, analysis of variance was adopted. If the data did not conform to normal distribution, nonparametric rank sum test was used for analysis. *p* < 0.05 was considered statistically significant.

## Results

### Identification of chemical constituents of SJC based on Q-Orbitrap high resolution liquid mass spectrometry

**FIGURE 1:**
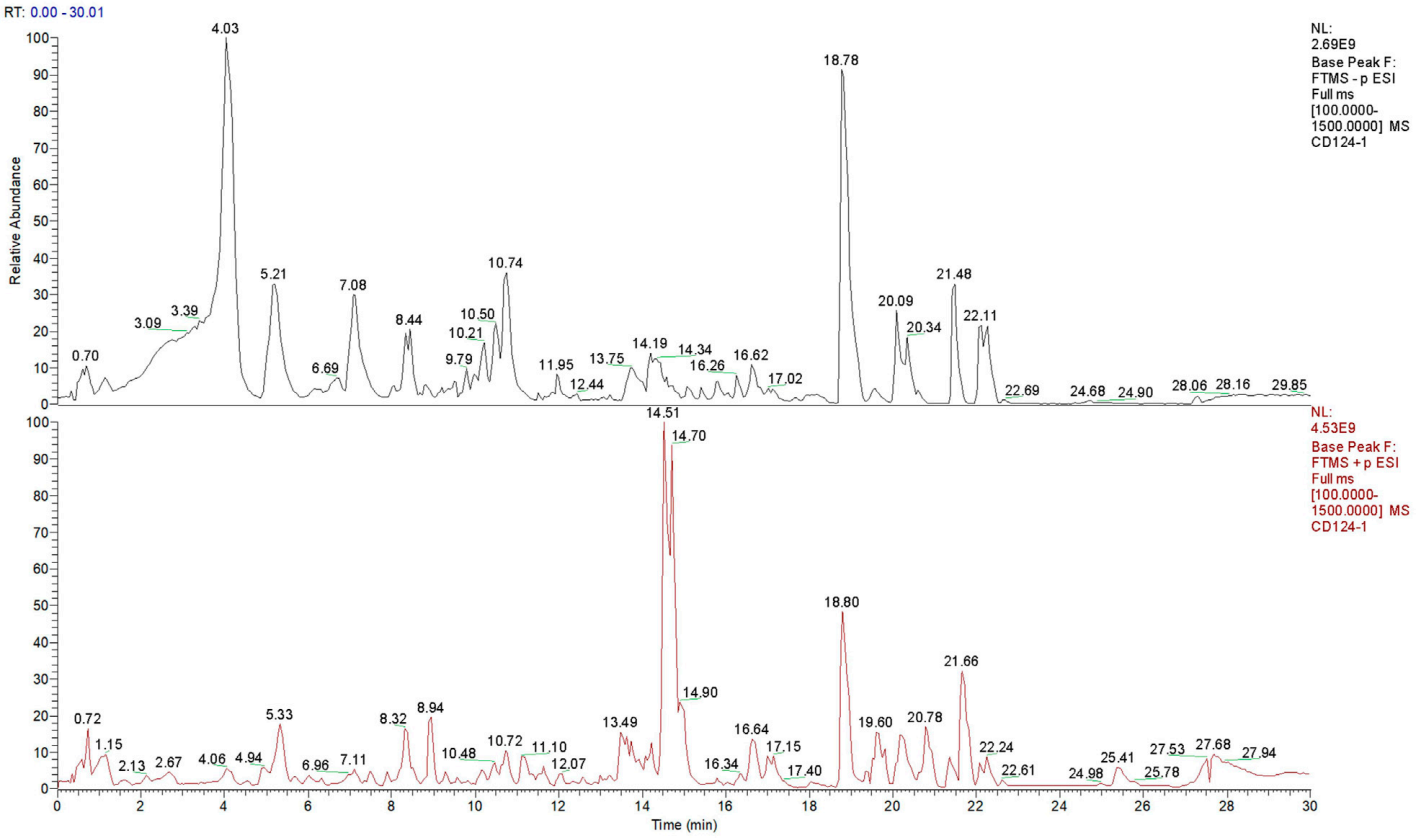
Total ion flow diagram of SJC.

The uPLC-Q-TOF/MS analysis method was used to analyze and identify the components contained in SJC. The collected data were preliminarily collated by CD2.1 (ThermoFisher) and then retrieved and compared to databases (Mz Cloud, Mz Vault). According to the identification results of Shi Jianchuan, 1231 compounds were matched in Mz Cloud (Figure 1), 445 compounds were matched in Mz Vault, and 195 compounds with comprehensive scores greater than 80 in Mz Cloud. A total of 161 compounds were identified after removing the repeated data in Mz Cloud, among which 28 compounds were mainly associated with tumors, including six terpenoids, four phenylpropanoids, three brasses, one steroid, one aldehyde, one phenolic acid and 12 other compounds.

### SJC inhibits the proliferation of esophageal cancer KYSE-150 cells and TE-1 cells

**FIGURE 2:**
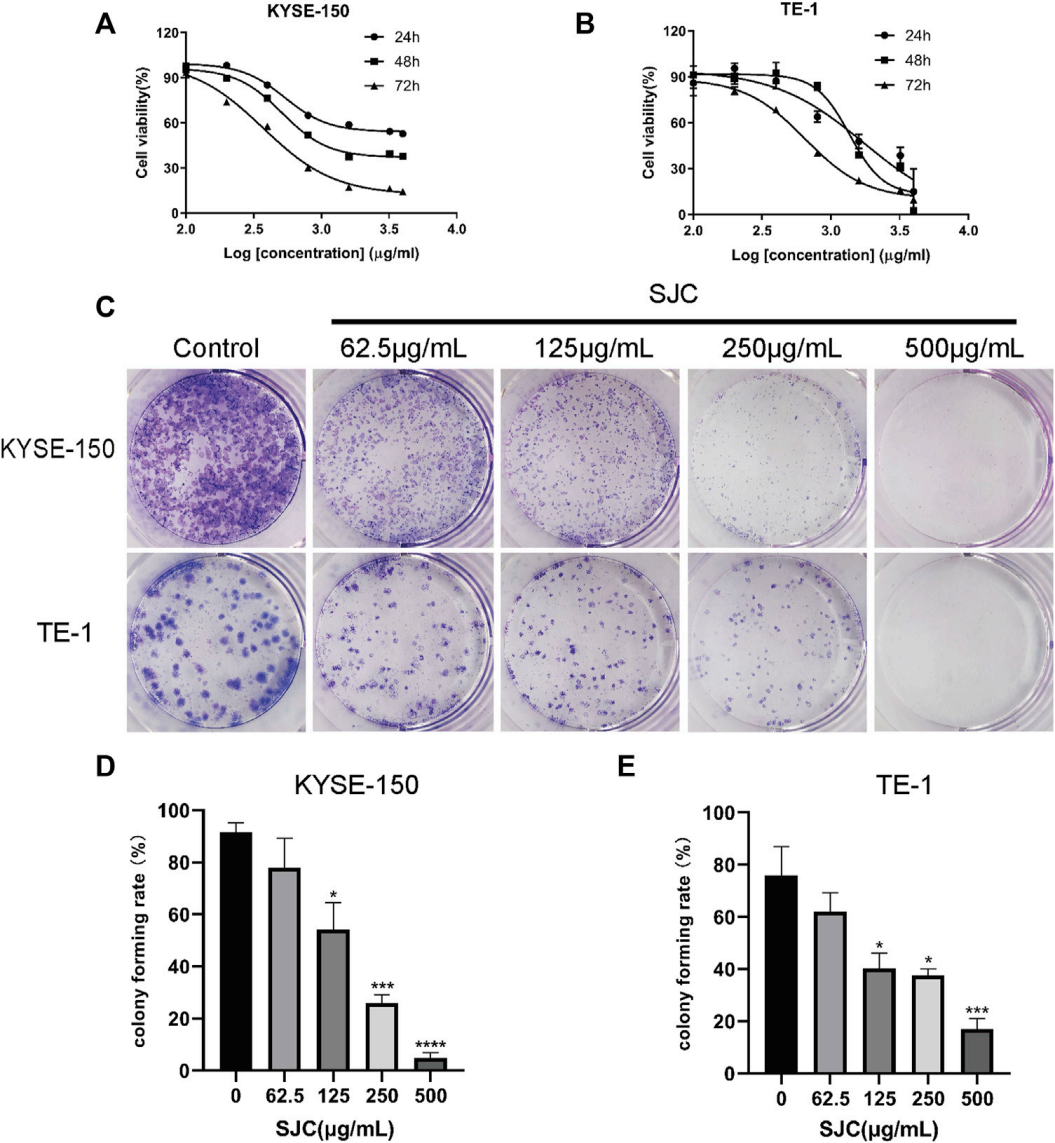
The inhibitory effect of SJC on esophageal cancer KYSE-150 cells and TE-1 cells. **(A)** CCK-8 assay was used to determine the sensitivity of esophageal carcinoma KYSE-150 cells to SJC. **(B)** CCK-8 assay was used to determine the sensitivity of esophageal carcinoma TE-1 cells to SJC. **(C)** Clonogenesis assay was used to detect the effect of SJC on the clonogenesis ability of KYSE-150 cells and TE-1 cells. **(D)** Histogram of quantitative analysis of relative clonal cell numbers of KYSE-150 cells in esophageal carcinoma. **(E)** Histogram of quantitative analysis of relative clonal cell numbers of TE-1 cells in esophageal carcinoma. Data are shown as mean ± SEM. *p < 0.05, **p < 0.01, ***p < 0.001, ****p < 0.0001 vs. control.

KYSE-150 cells and TE-1 cells were treated with different concentrations of SJC medium, and the data showed that the growth of both cell lines were inhibited in a dose-dependent manner. The IC50 values of KYSE-150 cells at 24, 48, and 72 h were 538.5, 512.5, and 371.4 μg/ml, respectively. The IC50 values of TE-1 cells at 24, 48, and 72 h were 1747, 1368, and 649.1 μg/ml, respectively (Figure 2A). In addition, the proliferation ability of KYSE-150 cells and TE-1 cells was evaluated using the plate clonogenesis assay, which showed that SJC effectively inhibited the clonogenesis ability of esophageal cancer cells in a dose-dependent manner. The inhibitory effect became more obvious with the increase of drug concentration (Figure 2B). According to the IC50 value, KYSE-150 cells with low differentiation degree were more sensitive to the effect of SJC than TE-1 cells with high differentiation degree. Therefore, KYSE-150 cells were selected for subsequent experiments. Functional test showed that SJC could inhibit the proliferation of esophageal cancer cells.

### Proteomics analysis of SJC on esophageal carcinoma KYSE-150 cells

**FIGURE 3:**
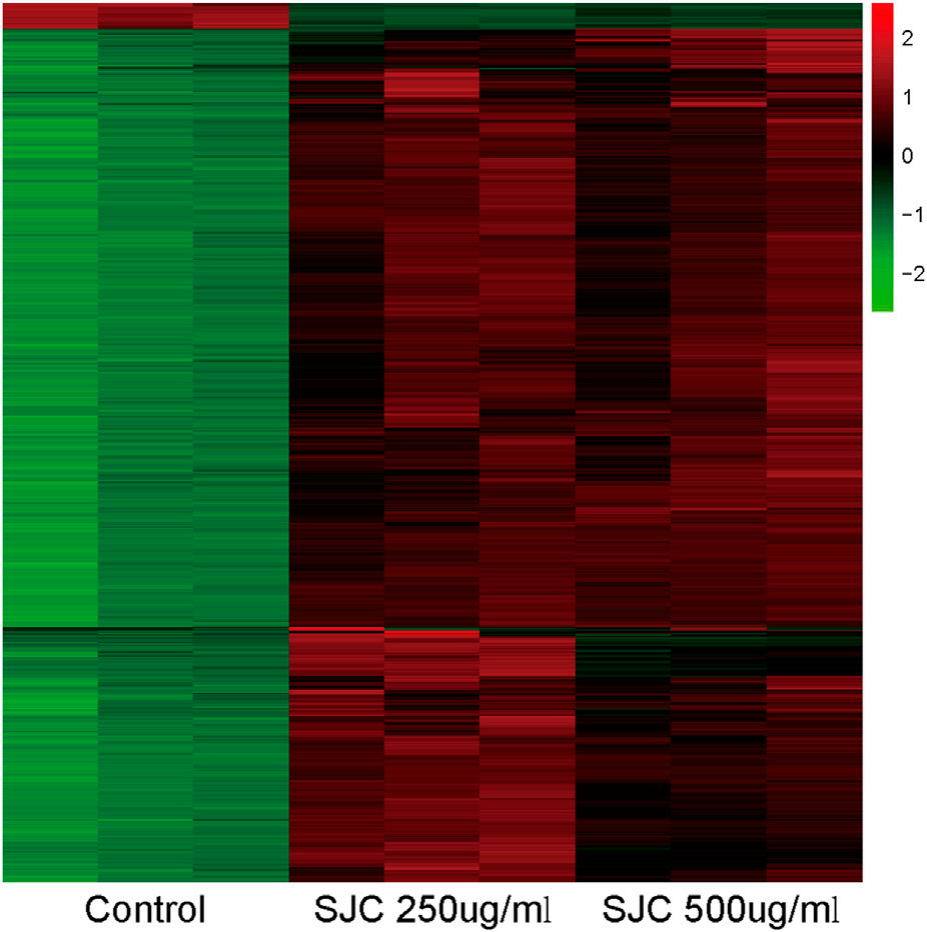
Heat maps of differential protein expressions in the control group, SJC 250 μg/ml, and SJC 500 μg/ml medication group. Up- and down-regulated genes are indicated in red and green, respectively.

To elucidate the inhibitory mechanism of SJC on esophageal carcinoma KYSE-150 cells, the protein content of KYSE-150 cells treated with SJC at the concentration of 0, 250, and 500 μg/ml for 48 h was detected by mass spectrometry. We used TMT labeling quantitative methods to perform high-resolution LC-MS/MS analysis. A total of 3872 proteins were identified with thresholds greater than 1.2 times and less than 0.83 times, *p* < 0.05, of which 2421 were up-regulated and 1451 were down-regulated. Log values of the expression levels of significantly differentially expressed proteins are shown in different colors in the heat map (Figure 3). Proteomic results showed that SJC could cause a large number of protein changes in KYSE-150 cells.

### KEGG enrichment analysis was performed for differential genes

**FIGURE 4:**
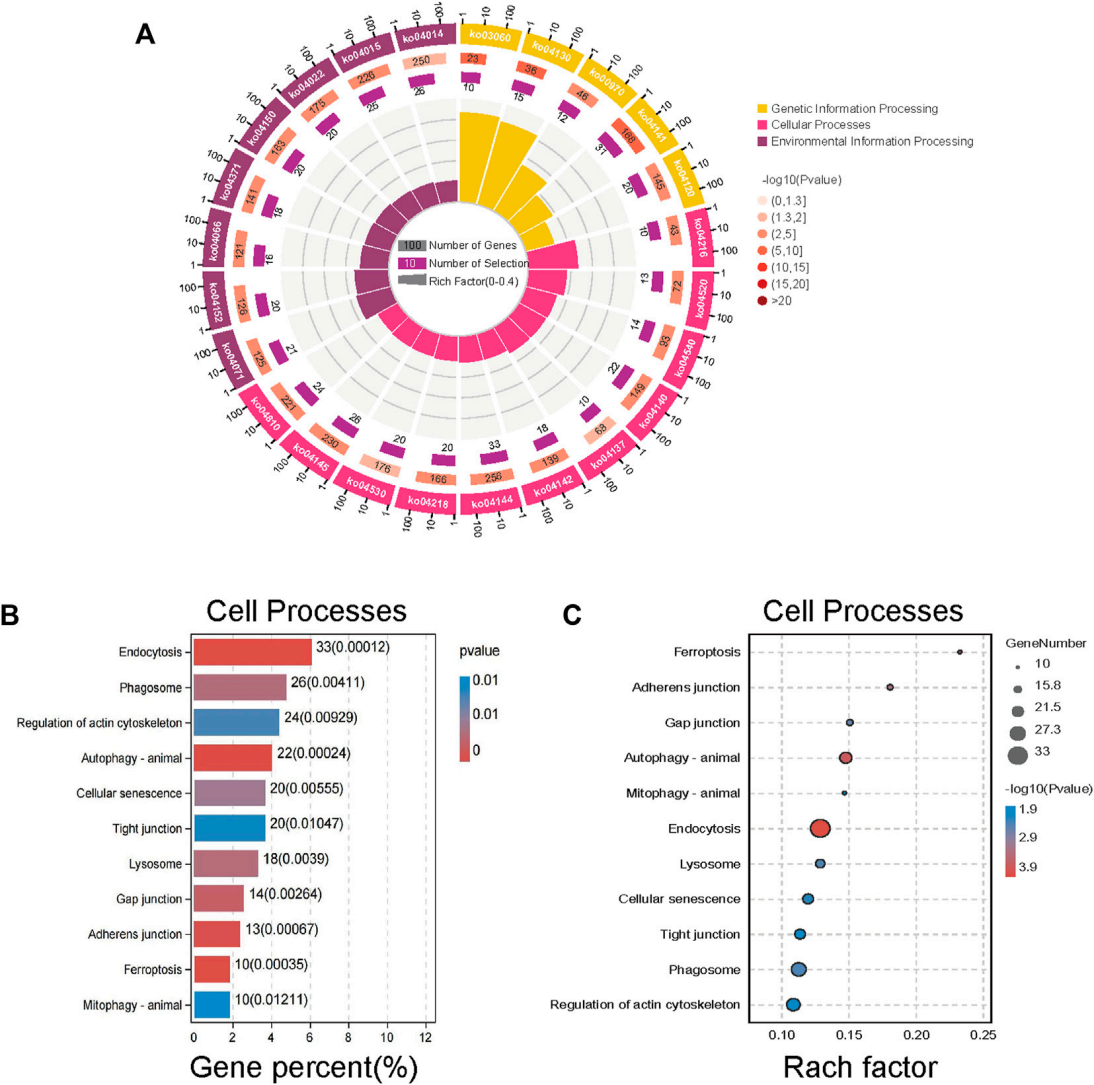
Kyoto Encyclopedia of Genes and Genomes (KEGG) enrichment analysis of different proteins expression. **(A)** An enrichment circle diagram shows the different classifications of KEGG enrichment analysis. **(B)** The bar chart of Cellular Processes, which are sorted by Gene percent,show the first 11 pathways of Cellular Processes. **(C)** The bubble chart of Cellular Processes, which are sorted by Rach factor,show the first 11 pathways of Cellular Processes. The more red the color, the lower the *p* value and the higher the confidence.

After screening, KEGG enrichment analysis was conducted using the online Omic Share Tools with the standard of *p* < 0.05 and Q < 0.05 to study whether there is a significant enrichment trend of differentially expressed proteins in certain functional types. The proportion of genetic information processing (including five pathways), environmental information processing (including eight pathways), and cellular processes (including 11 pathways) in the three categories related to this study, as well as the number of up-regulated genes in each category, are shown in the enrichment circle diagram (Figure 4A).

According to cell process classification (Figures 4B,C), the color of the graph indicates the significance of KEGG pathway enrichment, and the closer to red, the smaller the *p* value is, and the higher the KEGG pathway enrichment degree is. The analysis results showed that autophagy changed significantly in various related mechanisms, whether in terms of the degree of gene enrichment or *p*-value sequencing. Therefore, we evaluated on the anticancer mechanism of SJC on autophagy.

**FIGURE 5:**
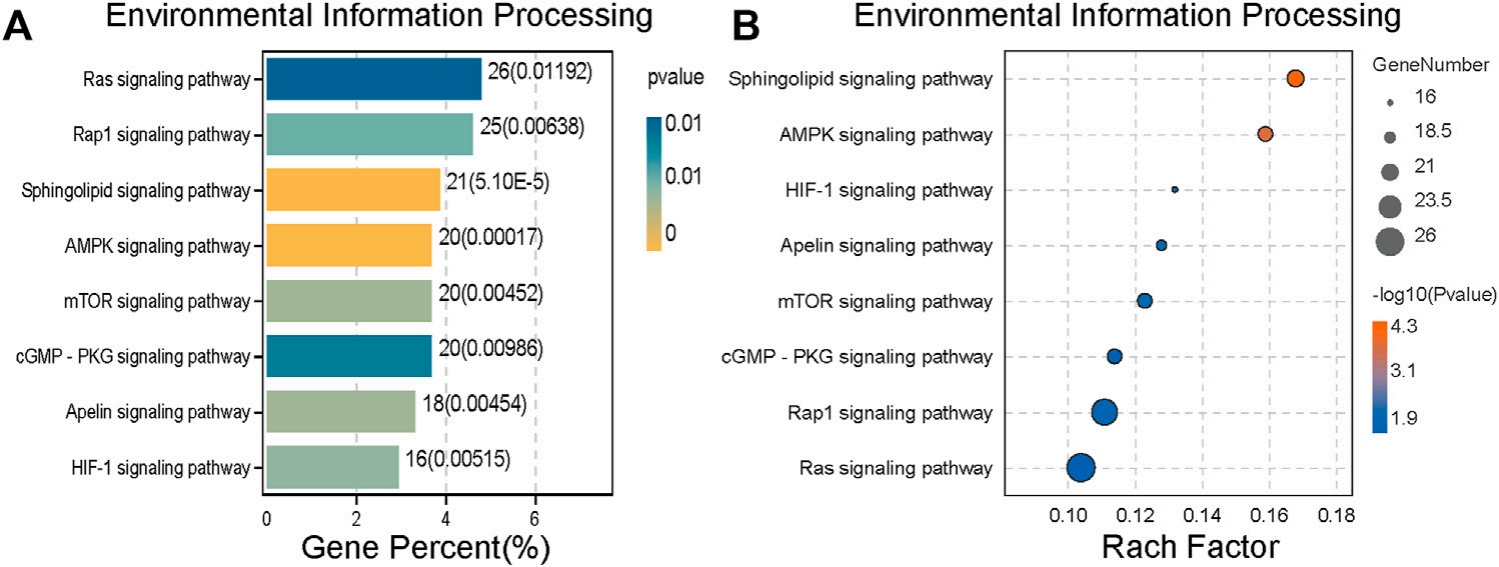
Environmental information processing of differential protein expression. **(A)** Histogram of Environmental Information Processing ranked by Gene Percent. **(B)** The bubble chart of Environmental Information Processing is sorted by the Rach factor. The bar chart and bubble chart show the top eight channels of Environmental Information Processing. The more yellow the color, the lower the *p* value and the higher the credibility.

The classification results of environmental information processing (Figure 5) show that AMPK signaling pathway has high relevance. AMPK is considered the “energy regulator” of cells, which is closely related to the occurrence of autophagy. It can directly activate the promoter protein ULK1 of autophagy and phosphorylate it to induce autophagy (Kim et al., 2011). Therefore, we hypothesized that SJC might activate autophagy in KYSE-150 cells through the AMPK/ULK1 signaling pathway.

### Autophagy-related proteins detected by WB

**FIGURE 6:**
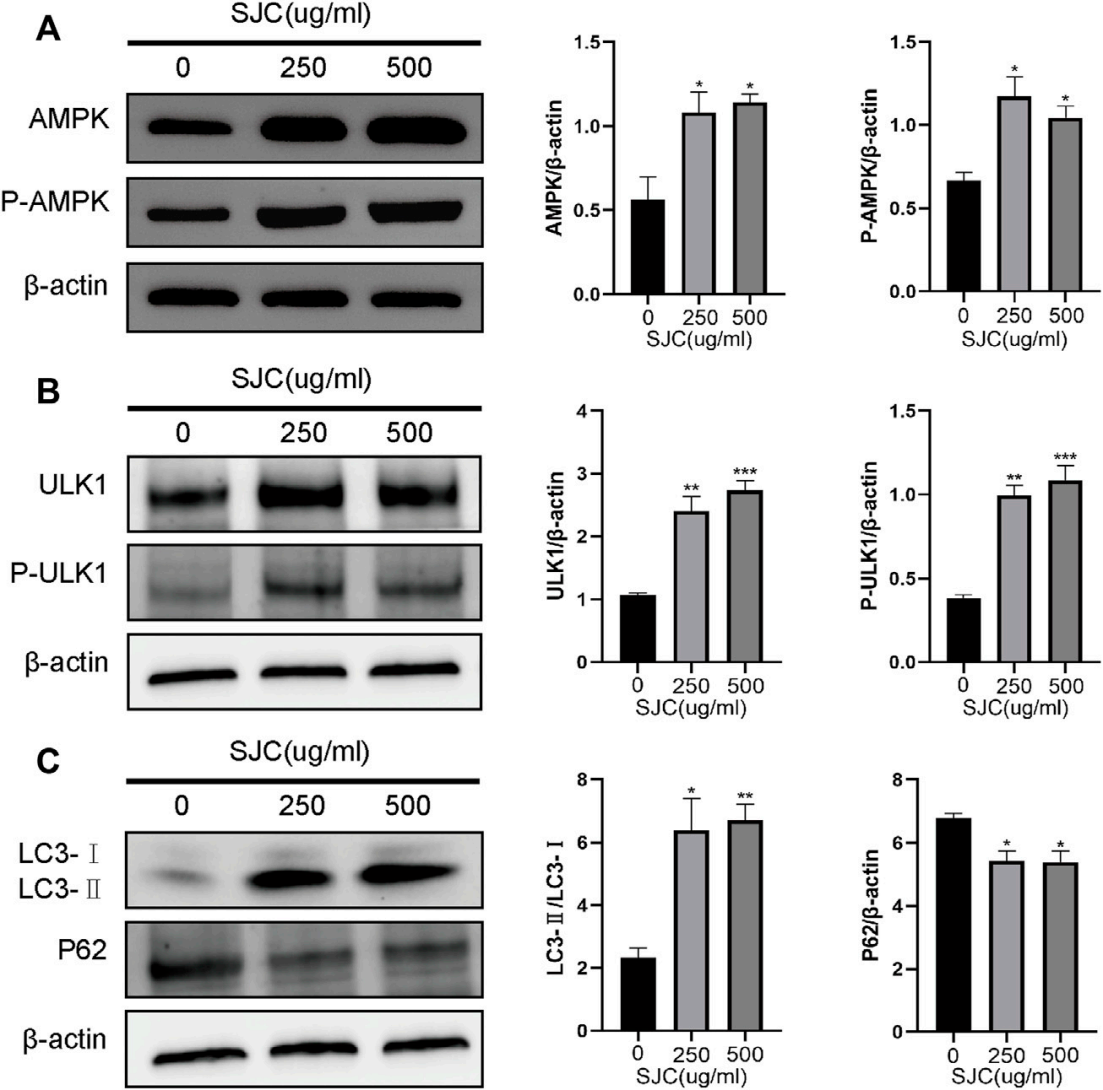
Differences in expression of AMPK-ULK1 signaling pathway and key proteins in autophagy in the control group, and the 250 and 500 μg/ml SJC groups detected by Western blotting. **(A)** The expression of AMPK and P-AMPK increased after SJC treatment. **(B)** The expression of ULK1 and P-ULK1 increased after SJC treatment. **(C)** The ratio of LC3-Ⅰ to LC3-Ⅱ was enhanced after SJC treatment. ImageJ software was used to calculate the band intensity. The experiments were repeated three times independently. The data were expressed as mean + SEM,*p < 0.05, **p < 0.01, ***p < 0.001, ****p < 0.0001 vs. control.

WB results showed that the expression of proteins AMPK and ULK1, that are the key proteins in autophagy, increased, and the expression of P-AMPK and P-ULK1 was also significantly increased after the treatment of SJC (Figures 6A,B). The transformation of LC3-ⅰ to LC3-ⅱ was used as a marker of autophagy. Compared to the control group, the transformation of LC3 was significantly enhanced in the SJC group (Figure 6C). P62 is a substrate of autophagy, and it can also bind with LC3 to form the p62-LC3 complex to mediate the degradation of ubiquitinated protein by autophagy. Its expression level can reflect autophagy activity, and WB results showed that the expression level of P62 decreased (Figure 6C).WB results verify the bioinformatics analysis results again.

### Confocal microscope observation of stably expressed sensGFP-stubRFP-LC3 protein

**FIGURE 7:**
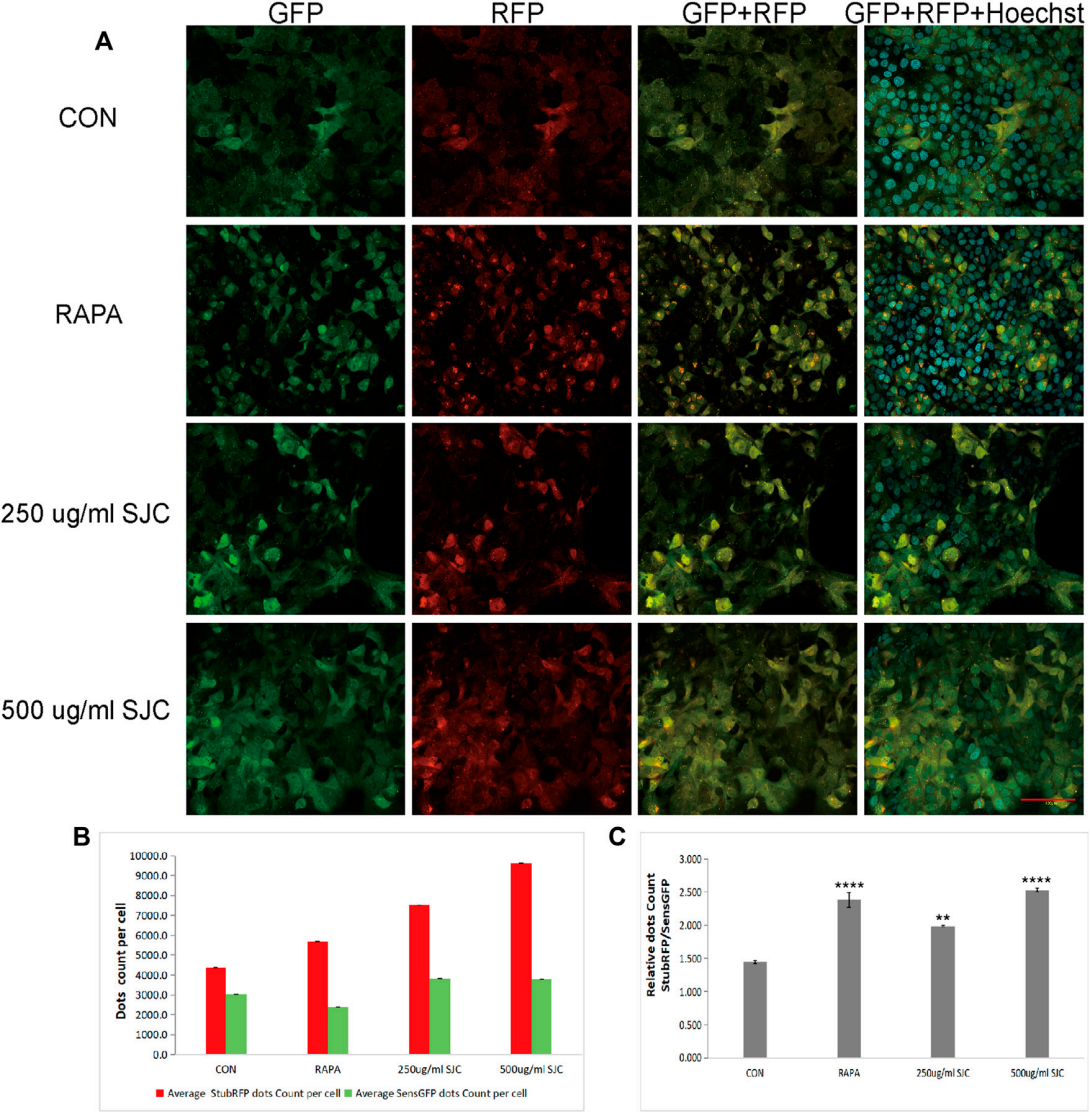
Esophageal carcinoma cells with stable expression of sensGFP-stubRFP-LC3 protein were established and observed under a confocal laser microscope. **(A)** Cell morphology and fluorescence expression after treatment by Rapa and SJC with different concentrations observed by confocal laser microscope. **(B)** Histogram of StubRFP and SensGFP expression of LC3 in each group. **(C)** Histogram of StubRFP/SensGFP ratios. Compared with CON, *p < 0.05, **p < 0.01, ***p < 0.001, ****p < 0.0001.

**FIGURE 8:**
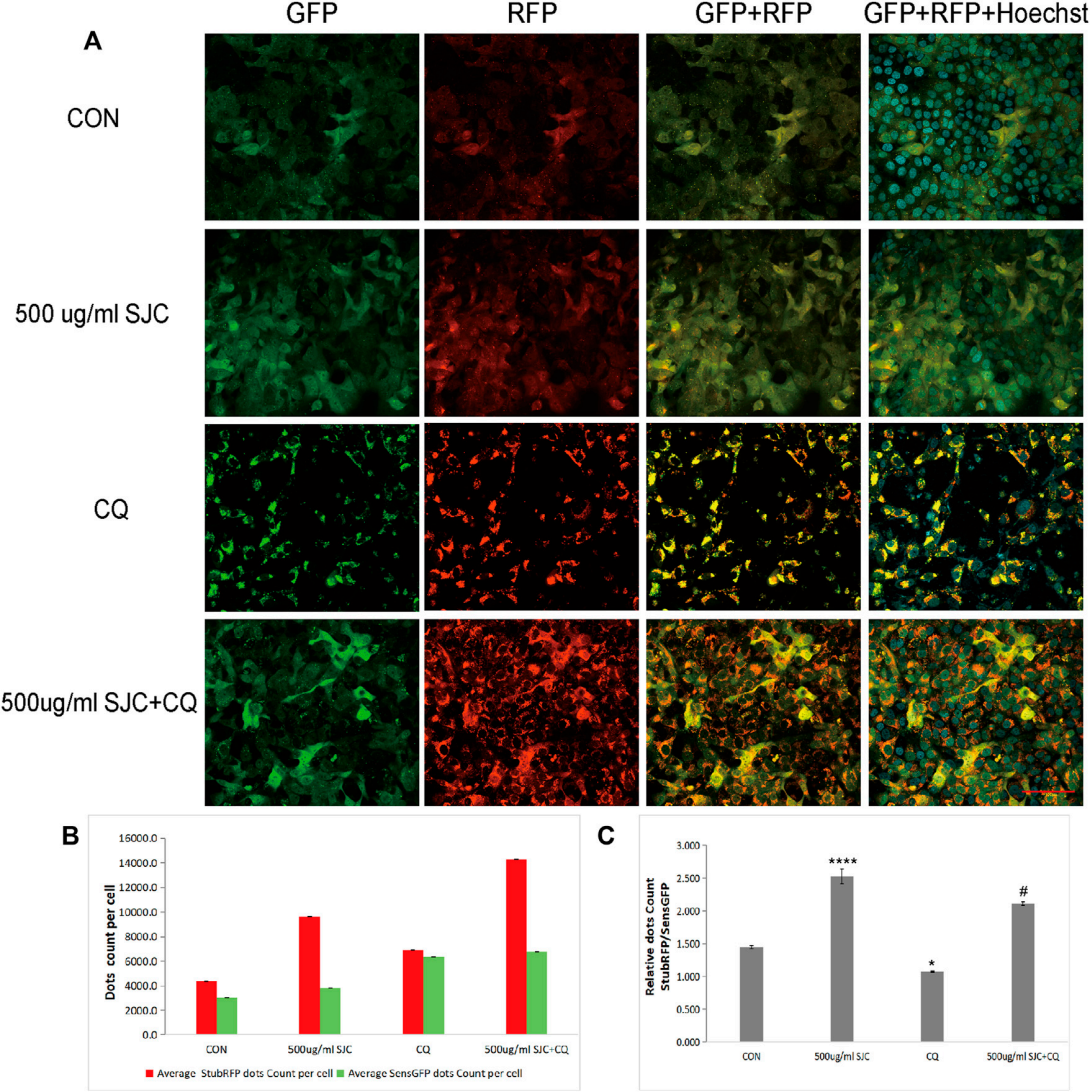
Differences in LC3 fluorescence expression in the SJC group before and after chloroquine treatment. **(A)** Cell morphology and fluorescence expression of the SJC group and the control group in the presence and absence of chloroquine were observed with confocal laser microscopy. **(B)** Histogram of StubRFP and SensGFP expression of LC3 in each group. **(C)** Histogram of StubRFP/SensGFP ratios. Compared with CON, *p < 0.05, **p < 0.01, ***p < 0.001, ****p < 0.0001; Compared with 500 μg/ml SJC, #*p* < 0.05.

To verify whether SJC can promote autophagy in esophageal cancer KYSE-150 cells, we established esophageal cancer cell lines with stable expression of sensGFP-stubRFP-LC3 by lentivirus transfection to evaluate autophagy flux. StubRFP expressed red fluorescence, SensGFP expressed green fluorescence. The number of StubRFP in cells represents the total number of autophagosomes and autophagolysosomes, and the number of SensGFP represents the number of autophagosomes, and the StubRFP/SensGFP ratio represents the extent of autophagy. As an autophagy promoter (Erfan et al., 2021), Rapamycin (Rapa) was set as the positive control group, and the StubRFP/SensGFP ratio of Rapa and different concentrations of SJC treatment were significantly enhanced compared to the control group. Compared to the Rapa group, 500 μg/ml SJC had a stronger autophagy promoting effect on KYSE-150 cells, and the effect of SJC on KYSE-150 cells showed a concentration-dependent relationship (Figures 7A–C). After transfection, the stably expressed sensGFP of sensGFP-StubrfP-LC3 protein can be quenched by lysosomes in acidic environment, while chloroquine can increase the pH value of lysosomes and ultimately inhibit the fusion of autophagosomes and lysosomes. This prevents the autophagosomes from maturing into autophagolysosomes and blocking the later stage of autophagy (Cai et al., 2019; Yu et al., 2021). After chloroquine treatment, the StubRFP and SensGFP increased slightly, but the StubRFP/SensGFP ratio was relatively low. However, when chloroquine was combined with the 500 μg/ml SJC group, both StubRFP and SensGFP were increased compared with the 500 μg/ml SJC group, but the StubRFP/SensGFP ratio was significantly decreased (Figures 8A–C), indicating that LC3 degradation was reduced and the fusion stage of autophagosome and lysosome was partially blocked. The increase of autophagy protein LC3 transformation was verified by cell fluorescence experiment.

### Observation of autophagosomes by electron microscopy

**FIGURE 9:**
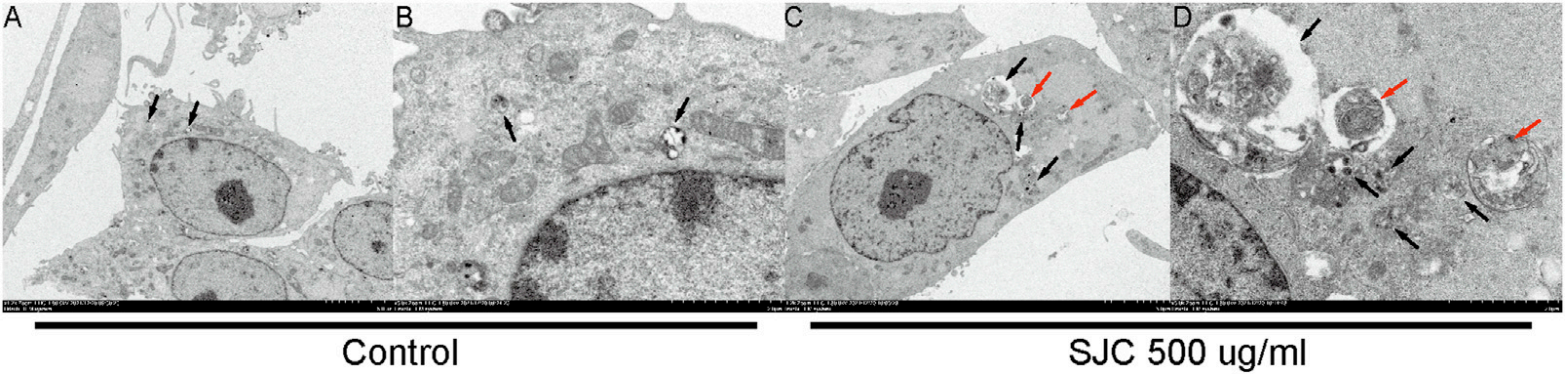
Autophagosome were observed under a transmission electron microscope. **(A**,**B)** were the normal control group, and there were some autophagolysosomes in early digestion stage. **(C**,**D)** show cells from the 500 μg/ml SJC treatment group, with many autophagosomes, mid-late autophagolysosomes, double-layer membrane structures, and no obvious organelle structures inside the cell. Autolysosomes are represented by black arrows and autophagosomes are represented by red arrows.

To verify our results from the proteomic analysis, we used electron microscopy to capture autophagosomes. Electron microscopy is the gold standard for the detection of autophagy. Autophagosomes are characterized by double-membranous vacuoles surrounding cytoplasmic organelles, such as mitochondria, which fused lysosomes. The lysosomes degrade the organelles in the vacuoles and form black granular monolayer membranous vacuoles. The cytoplasm of esophageal carcinoma KYSE-150 cells was characterized by double membrane vacuoles and autophagosomes with cytoplasmic components. As Figure 9 shows (Figure 9), compared with normal cells, the cells in the treatment group had more formation of autophagic vacuoles, and some organelles were encapsulated and degraded.

## Discussion

*Salvia chinensia* Benth, also known as SJC, is a common anti-cancer drug in traditional Chinese medicine. Studies have shown that SJC can inhibit the expression of the WT1 oncogene in liver cancer cells through the Wnt/β-catenin signaling pathway (Wang et al., 2017). However, as a commonly used drug for the treatment of esophageal cancer, the mechanism of action of SJC is limited. In this paper, the differential proteins were screened by large throughput proteomics method, and it was found that SJC could effectively promote autophagy in esophageal cancer cells, and the mechanism of action was verified in cells.

In this study, Q-Orbitrap high-resolution liquid chromatography-mass spectrometry was used to identify the main chemical constituents of SJC. The effects of SJC on the viability of esophageal cancer KYSE-150 cells and TE-1 cells were detected. From the experimental results, we can see SJC has a concentration-dependent inhibitory effect on both cells. TE-1 cells were derived from patients with more differentiated esophageal squamous cell carcinoma, while KYSE-150 is poorly differentiated. Compared to the IC50 of SJC, the inhibiting effect is more pronounced on KYSE-150 cells, so the follow-up experiments were completed with KYSE-150 cells. The results of the colony formation experiment showed that the ability of esophageal cancer cells to form cell aggregation in the drug group was inhibited, indicating that SJC can indeed inhibit the growth of esophageal cancer cells. On this basis, we conducted further mechanistic research on SJC.

The combined application of TMT technology and LC-MS/MS technology is the main method of proteomics research. This study was based on quantitative proteomic technology of TMT to compare the protein differences in SJC-treated cells and control cells. We identified 3,872 differentially expressed proteins, of which 2,421 were up-regulated and 1,451 were down-regulated. KEGG enrichment analysis showed that autophagy and AMPK signaling pathways were significantly up-regulated. AMPK is a key energy sensor and regulator for maintaining energy homeostasis in eukaryotic cells and can be activated by various factors, including environmental stress, starvation, and hypoxia (Hardie et al., 2012; Wang et al., 2022). As a positive regulator of autophagy, AMPK activates and stimulates autophagy by directly activating ULK1, thereby initiating the process of autophagy (Kim et al., 2011). Studies have shown that due to cellular and environmental stress, such as nutrient deprivation, external stimuli increase the AMP/ATP ratio, activating AMPK (Xie et al., 2014). Aspergillus induces autophagy in triple-negative breast cancer cells through the AMPK/ULK1 signaling axis (Cao et al., 2018). By activating the AMPK/ULK1 signaling pathway, aconitine can induce autophagy in H9c2 cardiomyocytes mediated by DNA oxidative damage (Wang et al., 2022). AMPK directly promotes the occurrence of autophagy by phosphorylating and activating ULK1, inducing the initiation of autophagy (Fan et al., 2015). In addition, branched-chain amino acids can activate AMPK, thereby increasing the phosphorylation of ULK1 and promoting cardiomyocyte autophagy (Jiang et al., 2021). Therefore, we speculate that SJC promotes autophagy in esophageal cancer cells through the AMPK/ULK1 signaling pathway. The WB results of this study showed that the expression levels of AMPK, P-AMPK, ULK1 and P-ULK1 were up-regulated after SJC treatment, indicating that SJC activated the AMPK/ULK1 signaling pathway.

Microtubule-associated protein 1 light chain 3 (LC3) is a key protein in the formation of autophagosomes, and it has two forms: LC3-I and LC3-II (Schlafli et al., 2016). LC3-I can bind to phosphatidylethanolamine (PE) to form LC3-II on the autophagic membrane, which redistributes from diffuse cytoplasmic localization to a distinctive speckled cytoplasmic pattern, revealing that LC3 is recruited to the autophagic vesicles (Kabeya, 2000; Zhou et al., 2013; Zheng et al., 2017). The punctate accumulation of LC3II in cells indicates autophagosome formation and elevated autophagy. However, LC3II can be degraded by autophagy, and the amount of LC3 at a particular time point does not fully indicate the autophagic flux (Mizushima and Yoshimori, 2014). Blocking the degradation of LC3 is conducive to a clearer understanding of the degree of autophagy promoted by SJC in esophageal cancer KYSE-150 cells. Stably expressed sensGFP-STUbrfP-LC3 protein carries SensGFP and StubRFP. SensGFP is sensitive to pH changes of autophagosome and lysosome fusion, while StubRFP is stable (Cai et al., 2019). Therefore, chloroquine was used in this study to block autophagosome and lysosome fusion. Confocal microscopy showed that marked LC3 fluorescence protein aggregation occurred after SJC treatment, and the number was significantly increased compared to the control group and Rapa group. The number of LC3 fluorescence in the combination of the SJC and chloroquine treatment group was higher than that of the combination of SJC treatment group, indicating that the combination of drugs partially inhibited the degradation of autophagosomes, and further documenting that SJC promotes autophagy in esophageal cancer KYSE-150 cells. P62 protein acts as an adaptor protein in the signal transduction pathway, mediates various biological functions of cells, and plays a central role in selective autophagy (Wang et al., 2021). The p62 protein binds to ubiquitinated protein aggregates, interacts with LC3 through the LC3-recognition sequence (LRS), and delivers the substrate to the autophagosome by forming the LC3-p62 complex. Finally, the substrate is degraded through autophagolysosome (Ichimura et al., 2014; Adams et al., 2016). Therefore, the level of p62 is often used as a marker for detecting autophagy (Wang et al., 2021), as an increase in autophagy activity will lead to a decrease in p62. The results of WB showed that SJC could significantly up-regulate the expression of LC3 protein and decrease the expression of P62. These results indicated that after SJC treatment, intracellular autophagosomes and autophagolysosome were significantly increased, accompanied by the degradation of cellular contents. Autophagy flux analysis demonstrated that SJC-induced autophagy in esophageal cancer KYSE-150 cells.

Autophagy is a self-degrading process that promotes cellular survival responses to nutrient starvation or stress conditions by removing damaged proteins and organelles (Xie et al., 2014). Excessive autophagy can degrade normal protein and organelle functions in tumor cells, destroy cells, and even cause programmed cell death in non-apoptotic pathways (Sun et al., 2022). The formation of autophagosomes is a hallmark of autophagy (Zhang L et al., 2022). The electron microscope experiments in this study observed that esophageal cancer KYSE-150 cells underwent robust autophagy, which indicated that SJC could effectively promote autophagy in esophageal cancer KYSE-150 cells.

Targeting autophagy is considered a new therapeutic strategy (Allaire et al., 2019). Our findings suggest that SJC can inhibit the growth of esophageal tumor cells and achieve antitumor effects by promoting autophagy. However, it is worth noting that the clinical application of SJC is often the whole herb used as a prescription. This study only clarifies its main chemical components. In future research, we will screen the composition of SJC and clarify its active ingredients. In addition, in the cloning test, SJC has different inhibitory effects on esophageal cancer cells with varying degrees of differentiation, which is also worthy of further research, or can guide the clinical application of SJC to patients with different degrees of differentiation of esophageal cancer.

## Data Availability

The original contributions presented in the study are included in the article/Supplementary Material. The mass spectrometry proteomics data presented in the study are deposited in the ProteomeXchange repository, accession number PXD035823. Further inquiries can be directed to the corresponding author.
