# Supplementary material for: Salvia chinensia Benth induces autophagy in esophageal cancer cells via AMPK/ULK1 signaling pathway
Source: Front Pharmacol. 2022 Sep 2;13:995344. doi: 10.3389/fphar.2022.995344 (PMC9478658; doi:10.3389/fphar.2022.995344)
Supplement: Supplementary file 2 [file DataSheet7.docx]

**https://www.jianguoyun.com/p/DW0LrBUQwonaChjJ_8YEIAA**

**（Analysis of the components of SJC）**

**https://www.jianguoyun.com/p/DXtTekIQwonaChjK_8YEIAA**

**（original data of CCK-8）**

**https://www.jianguoyun.com/p/DWqlruUQwonaChjL_8YEIAA**

**（original data of Plate colony formation method）**

**https://www.jianguoyun.com/p/DYGaPocQwonaChjP_8YEIAA**

**（original data of proteomics）**

**https://www.jianguoyun.com/p/DQF6CqwQwonaChjQ_8YEIAA （original data of WB）**

**https://www.jianguoyun.com/p/Da6DOZAQwonaChjU_8YEIAA**

**https://www.jianguoyun.com/p/DR2gHb8QwonaChj6z8sEIAA**

**（original data of sensGFP-stubRFP-LC3）**

**https://www.jianguoyun.com/p/DeEfXXoQwonaChjV_8YEIAA**

**（original data of transmission electron microscope）**
