# Supplementary material for: Salvia chinensia Benth induces autophagy in esophageal cancer cells via AMPK/ULK1 signaling pathway
Source: Front Pharmacol. 2022 Sep 2;13:995344. doi: 10.3389/fphar.2022.995344 (PMC9478658; doi:10.3389/fphar.2022.995344)
Supplement: Supplementary file 4 [file DataSheet1.ZIP › Analysis of the components of SJC/Total ion flow diagram.docx]

Note: Black shows the total ion flow chart in column 1, and red shows the total ion flow chart in column 2
