# Supplementary figures and images for: Salvia chinensia Benth induces autophagy in esophageal cancer cells via AMPK/ULK1 signaling pathway
Source: Front Pharmacol. 2022 Sep 2;13:995344. doi: 10.3389/fphar.2022.995344 (PMC9478658; doi:10.3389/fphar.2022.995344)

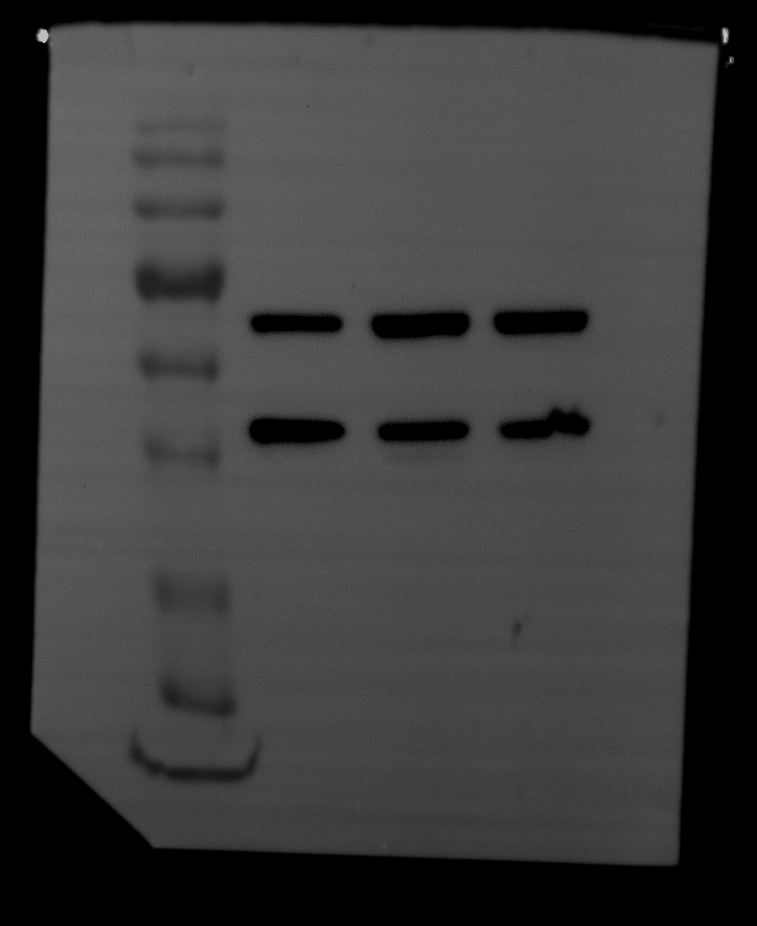

Supplement: Supplementary file 5 [file DataSheet6.ZIP › original data of WB/AMPK/AMPKí¬í¬1.tif]

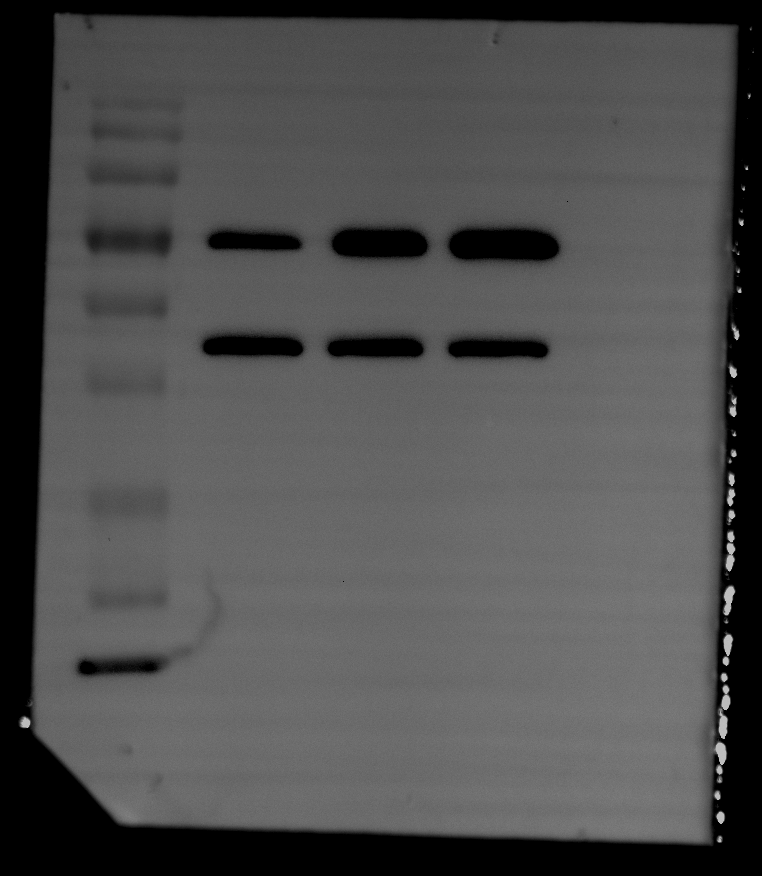

Supplement: Supplementary file 5 [file DataSheet6.ZIP › original data of WB/AMPK/AMPKí¬í¬2.tif]

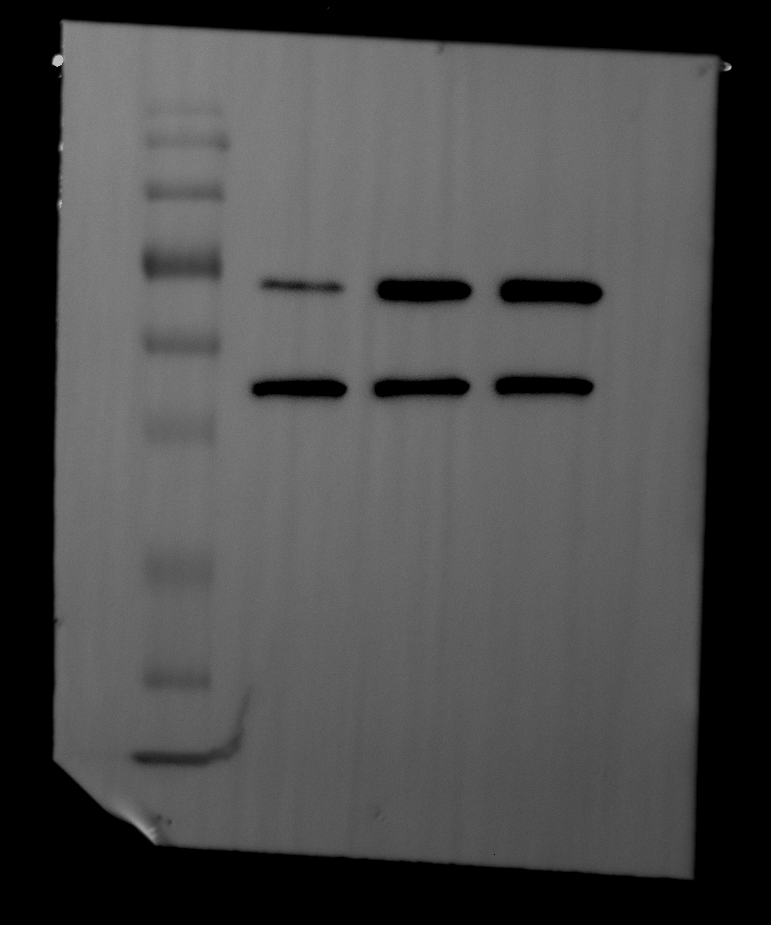

Supplement: Supplementary file 5 [file DataSheet6.ZIP › original data of WB/AMPK/AMPKí¬í¬3.tif]

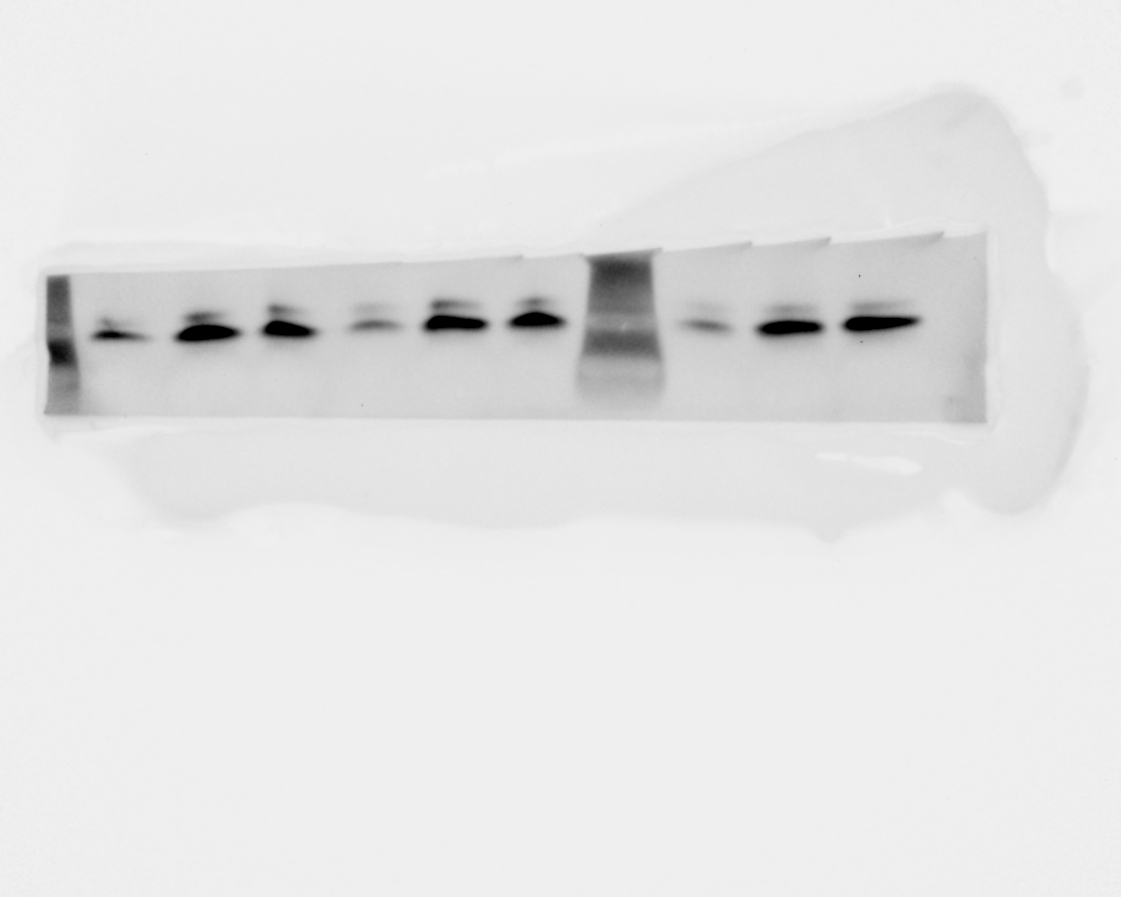

Supplement: Supplementary file 5 [file DataSheet6.ZIP › original data of WB/LC3/LC3.jpg]

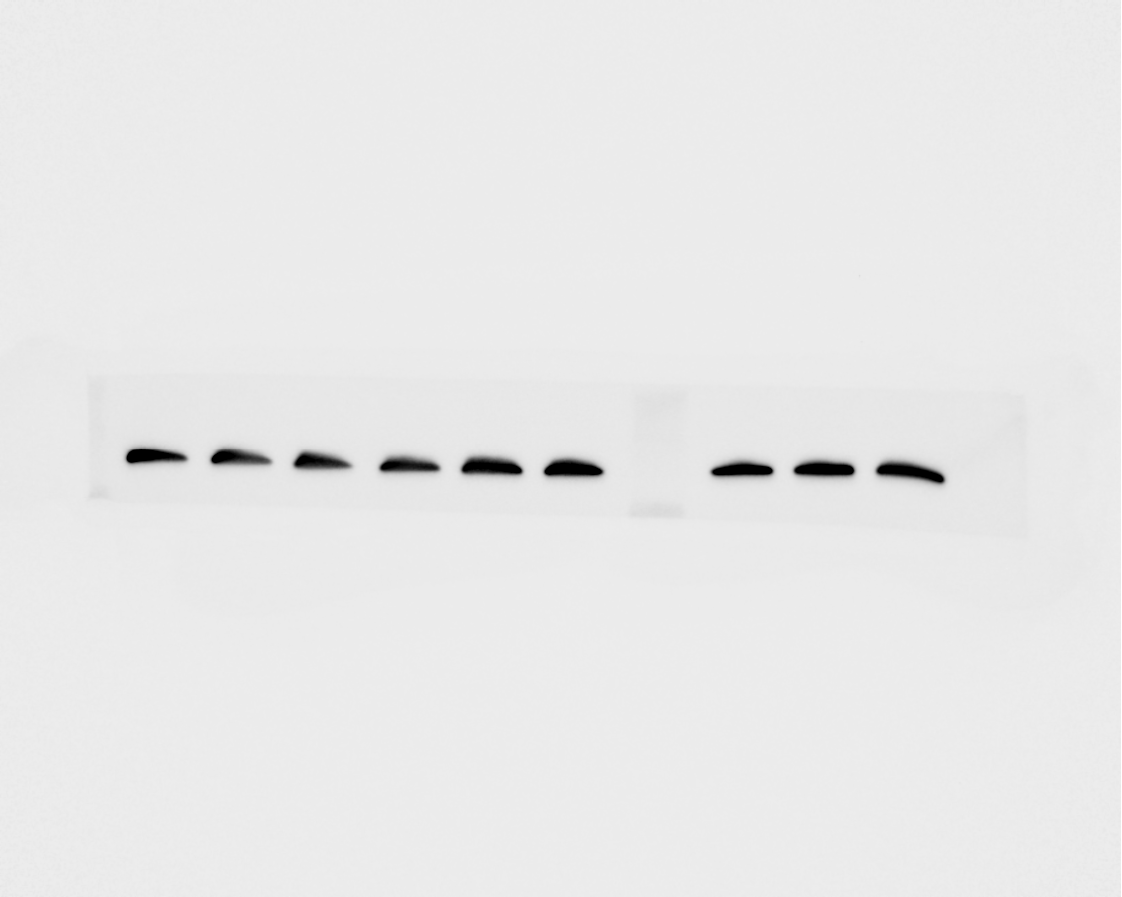

Supplement: Supplementary file 5 [file DataSheet6.ZIP › original data of WB/LC3/a┬-actin.tif]

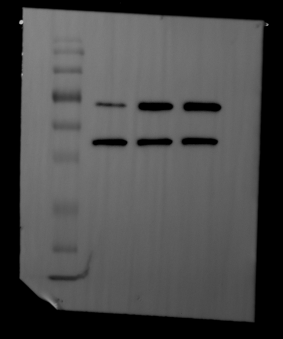

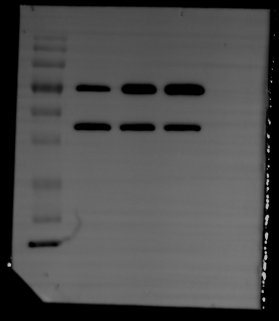

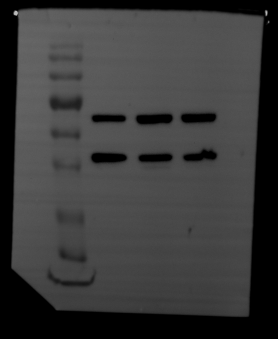
AMPK


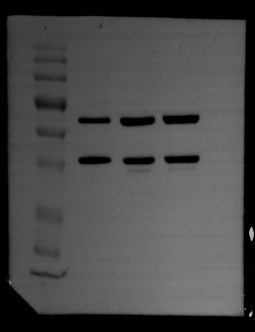

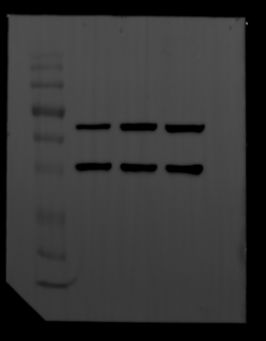

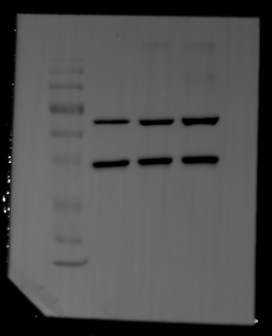
P-AMPK


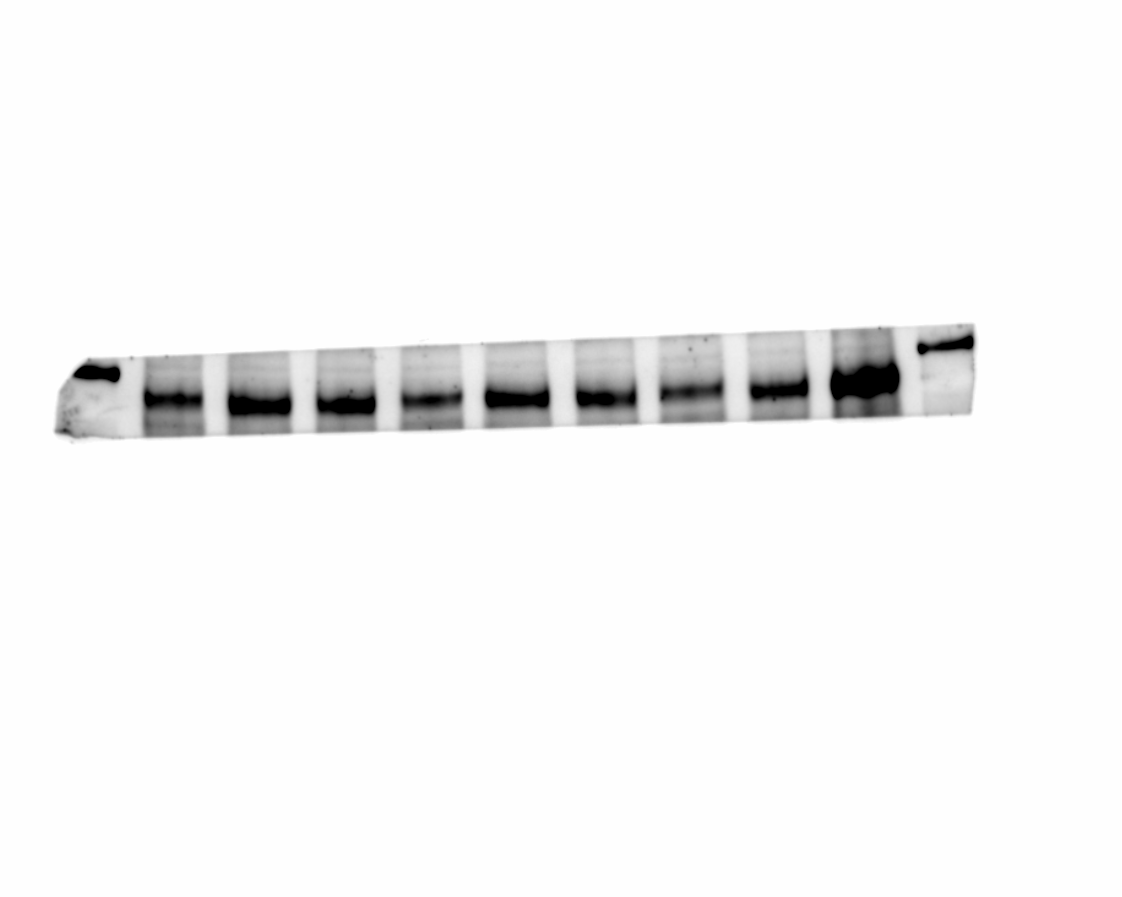

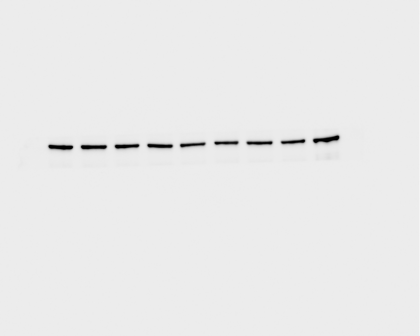

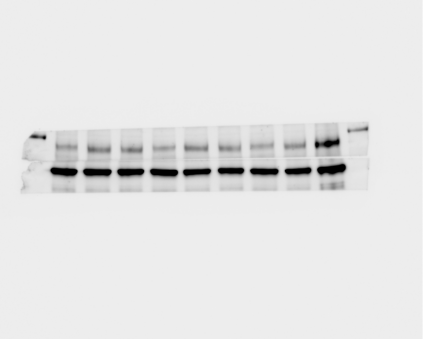


ULK1+β-actin

β-actin

ULK1


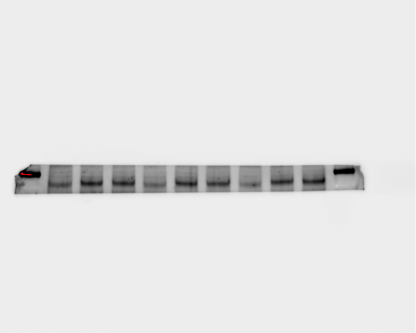

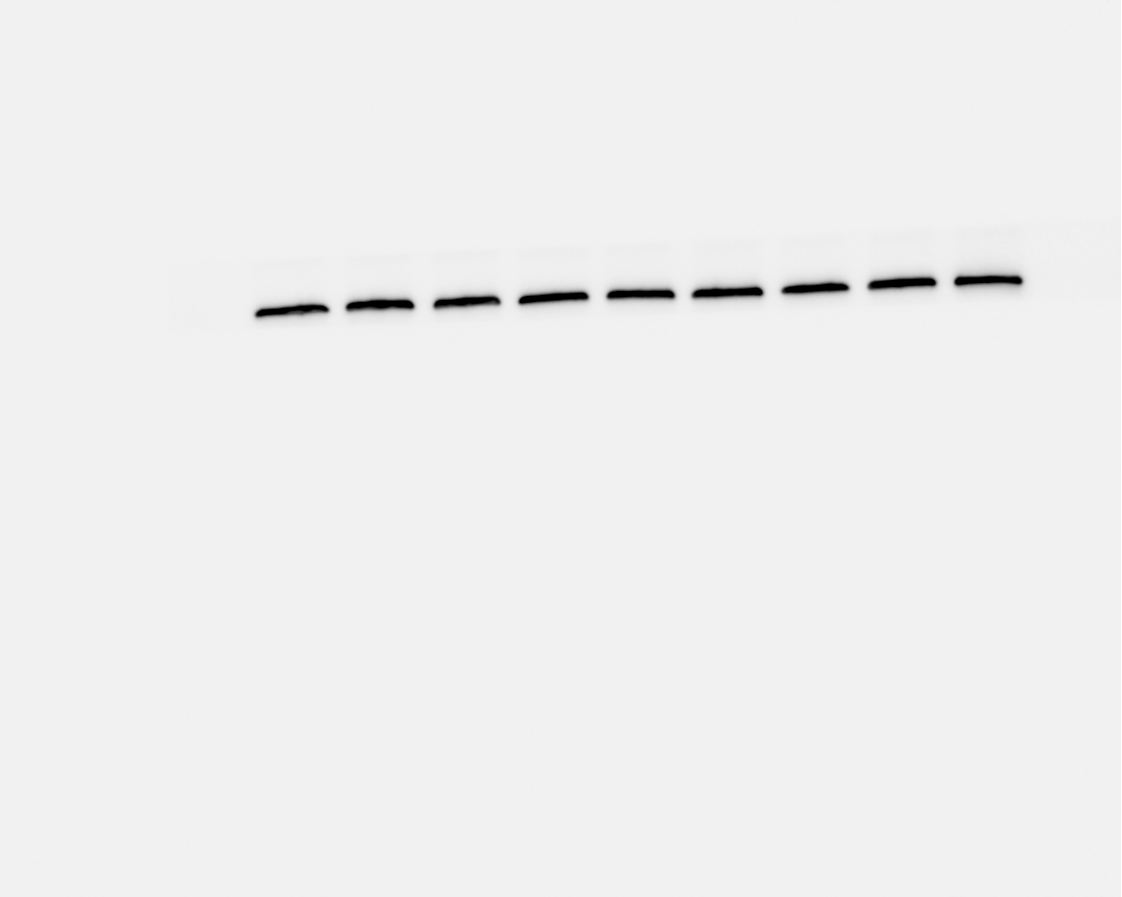

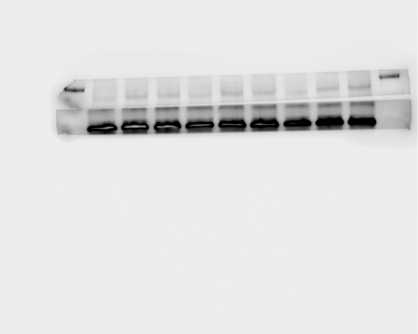


P-ULK1+β-actin

β-actin

P-ULK1


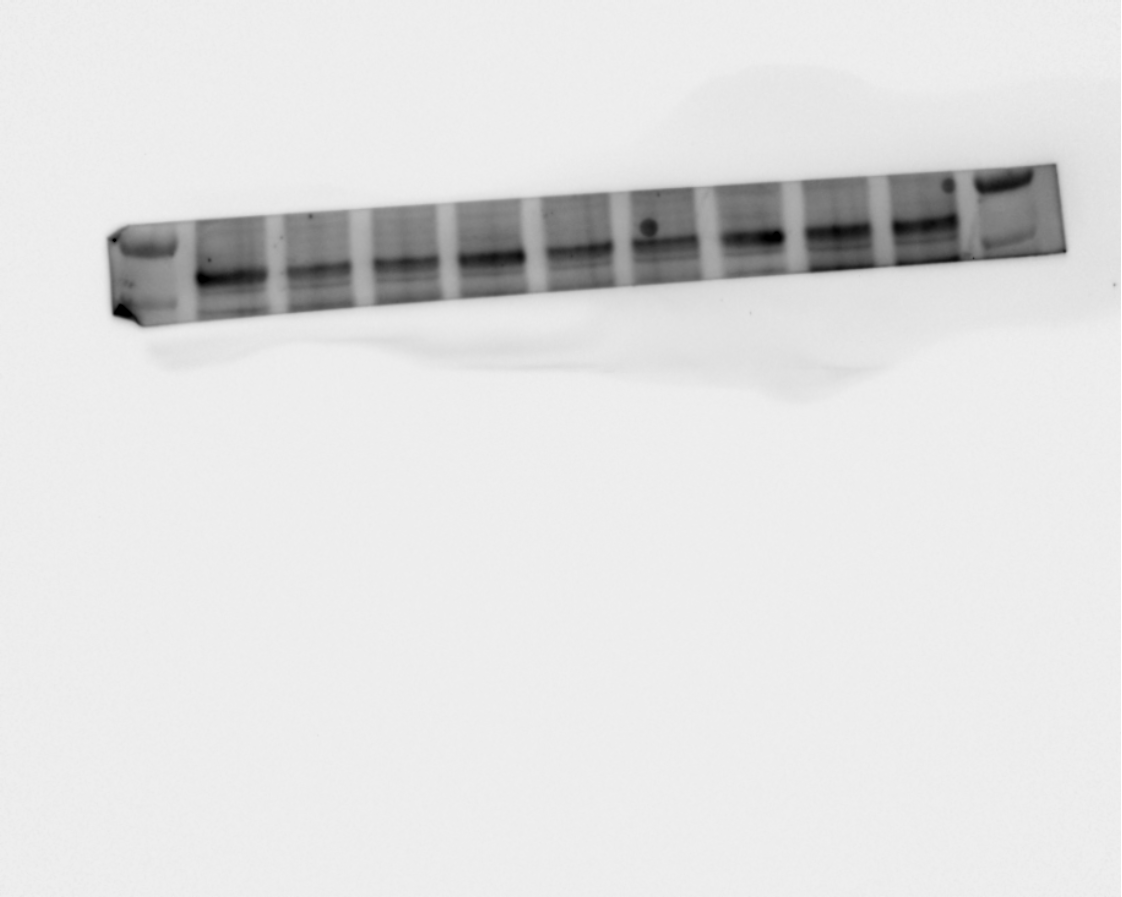

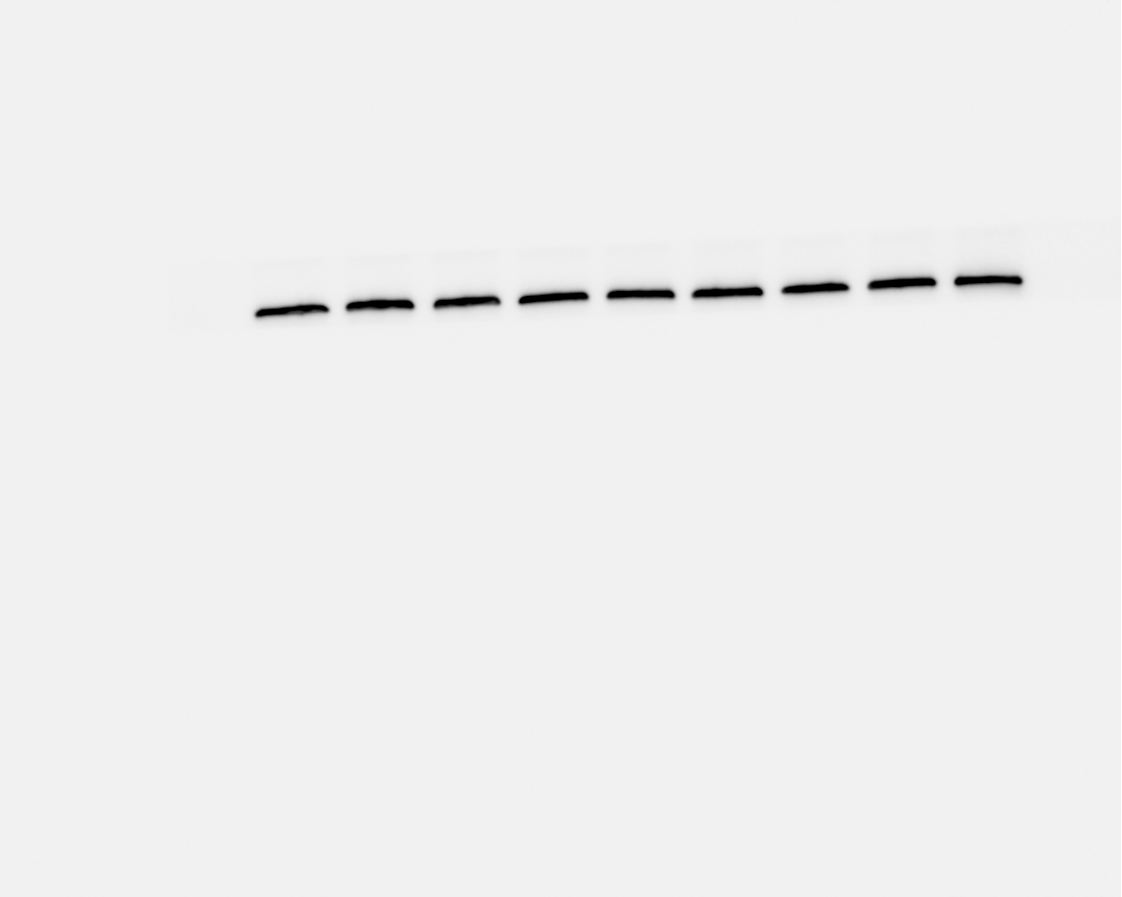

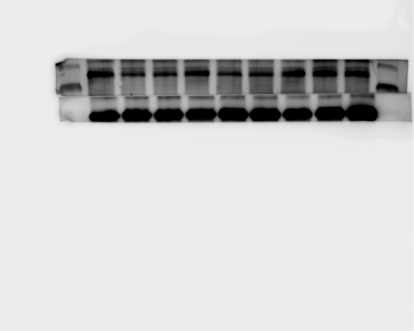


β-actin

P62+β-actin

P62


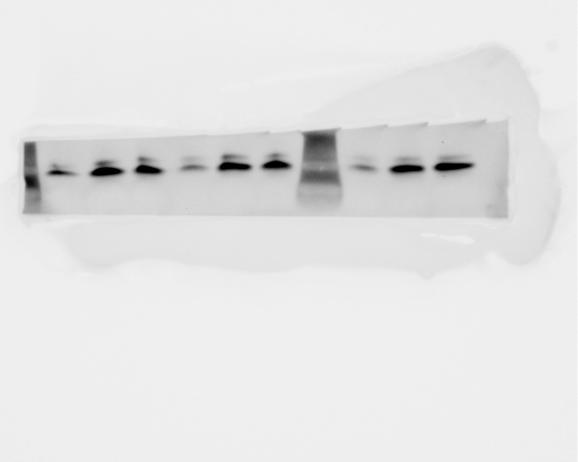

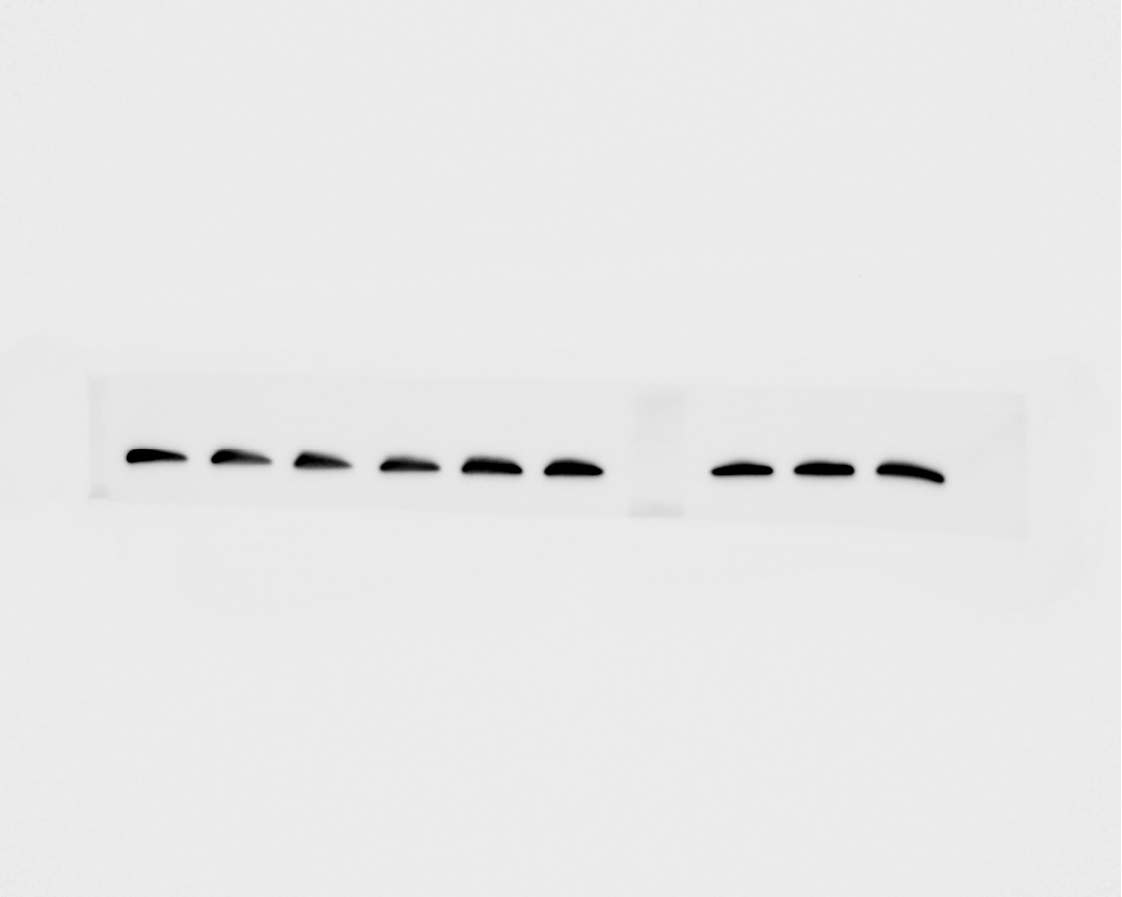


β-actin

LC3

Supplement: Supplementary file 5 [file DataSheet6.ZIP › original data of WB/original data of WB.docx]

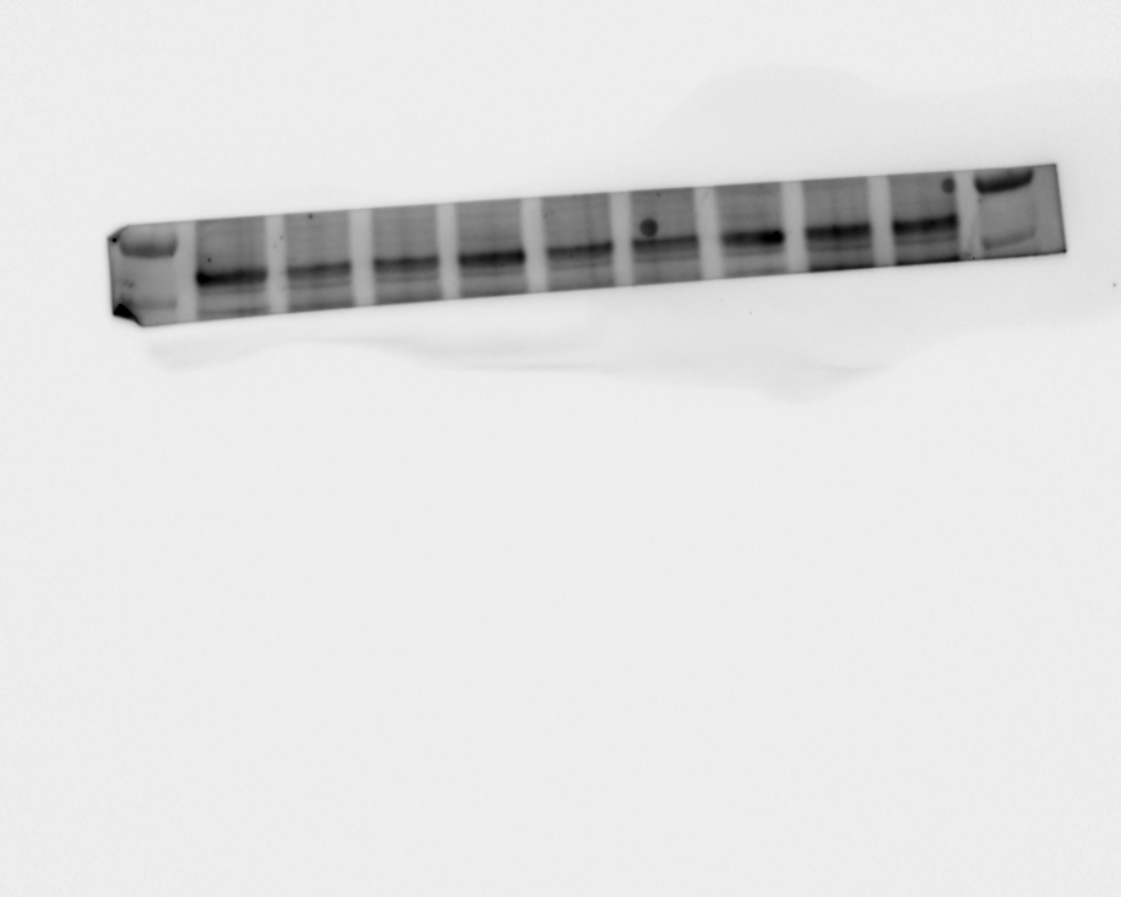

Supplement: Supplementary file 5 [file DataSheet6.ZIP › original data of WB/P62/P62.tif]

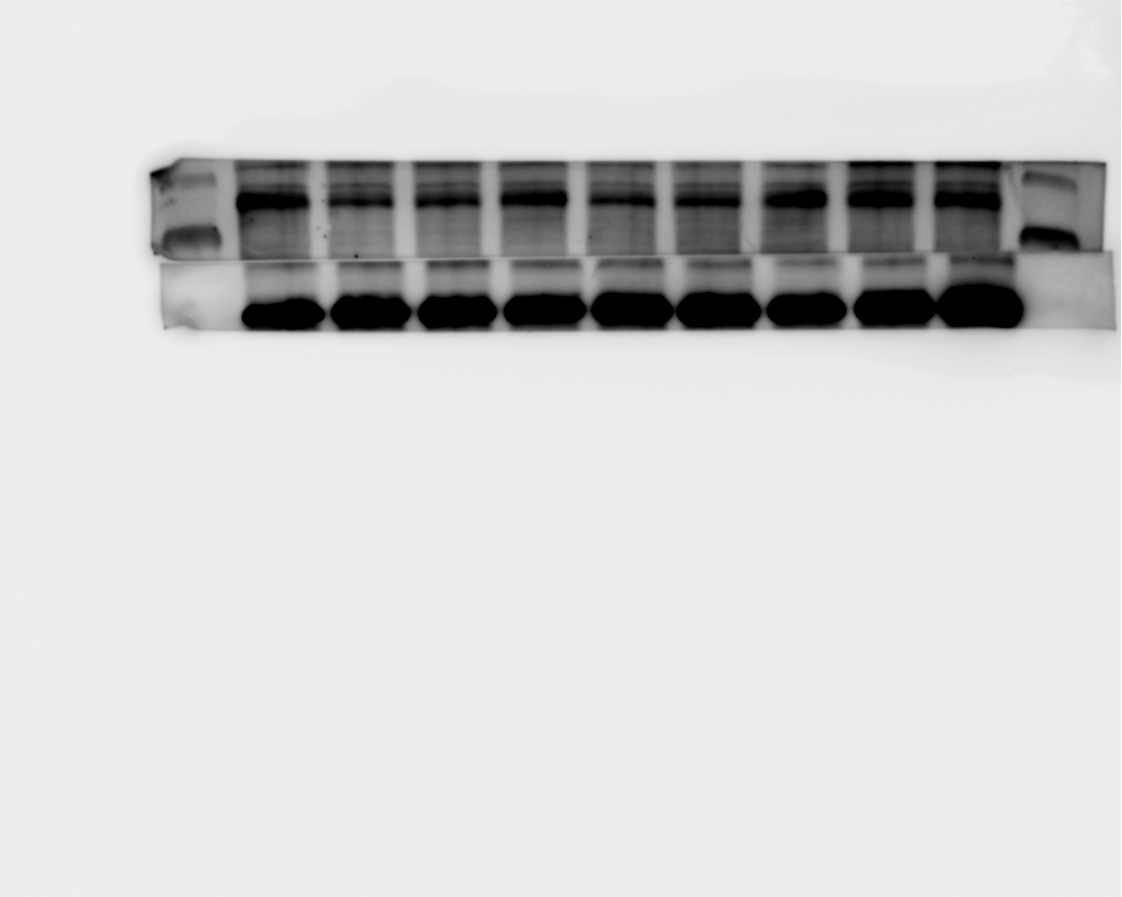

Supplement: Supplementary file 5 [file DataSheet6.ZIP › original data of WB/P62/P62+a┬-actin.tif]

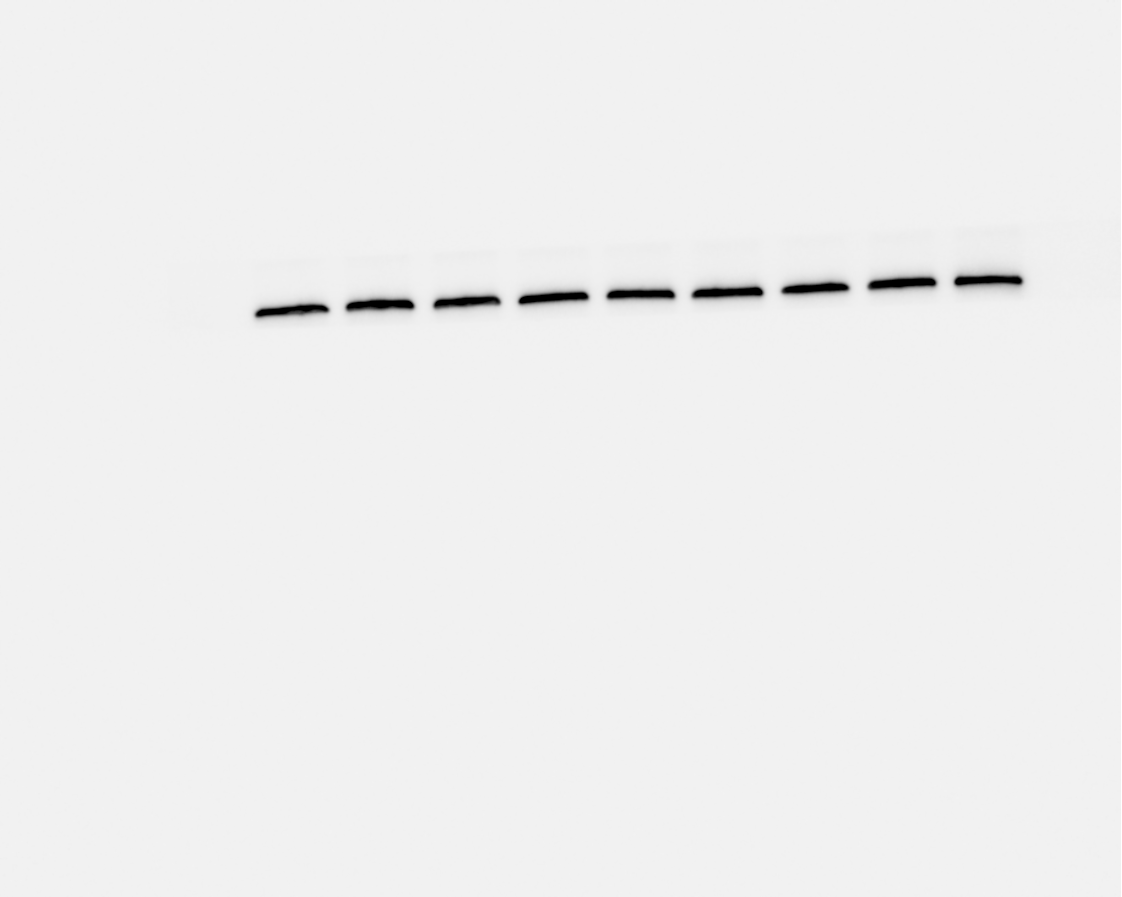

Supplement: Supplementary file 5 [file DataSheet6.ZIP › original data of WB/P62/a┬-actin.tif]

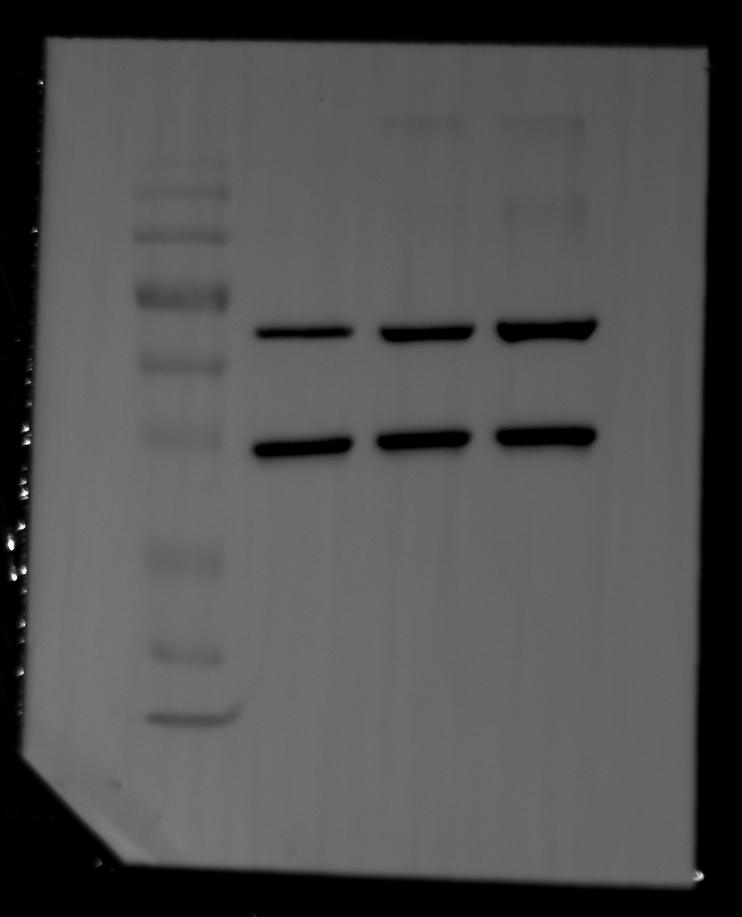

Supplement: Supplementary file 5 [file DataSheet6.ZIP › original data of WB/P-AMPK/P-AMPKí¬í¬1.tif]

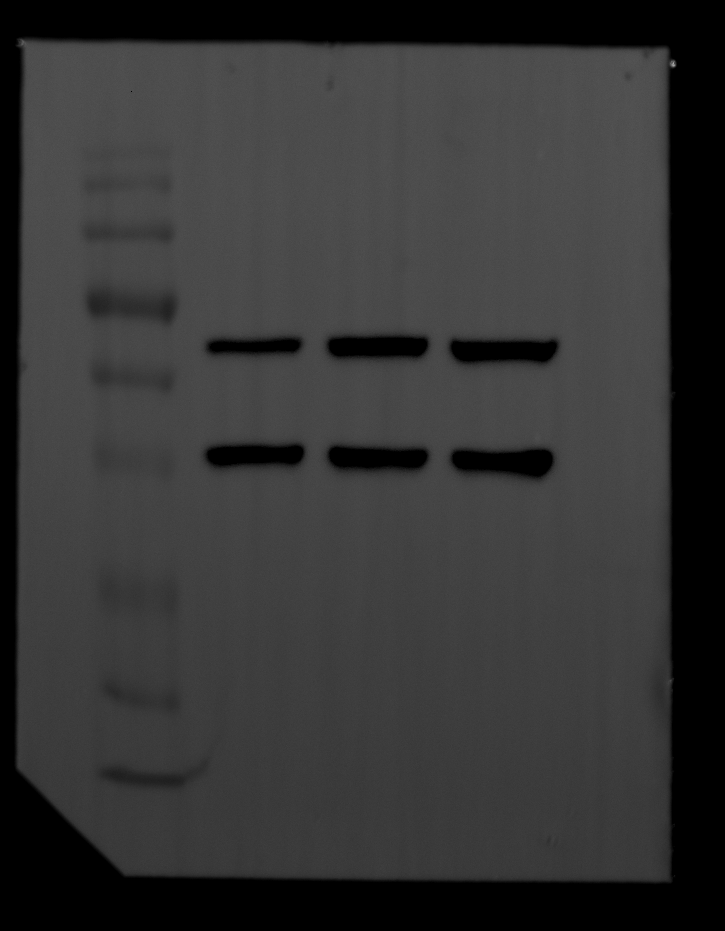

Supplement: Supplementary file 5 [file DataSheet6.ZIP › original data of WB/P-AMPK/P-AMPKí¬í¬2.tif]

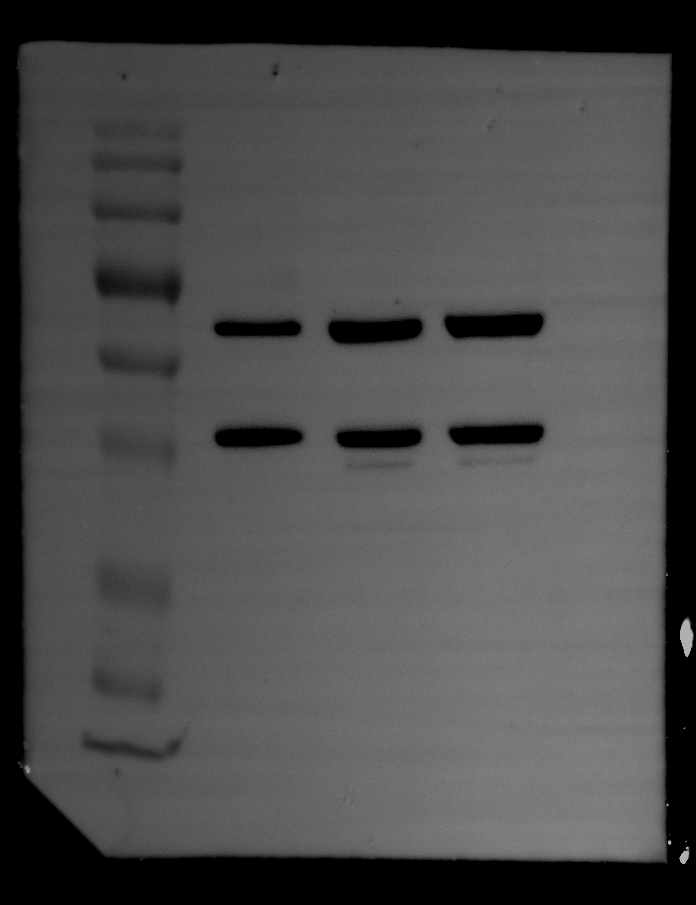

Supplement: Supplementary file 5 [file DataSheet6.ZIP › original data of WB/P-AMPK/P-AMPKí¬í¬3.tif]

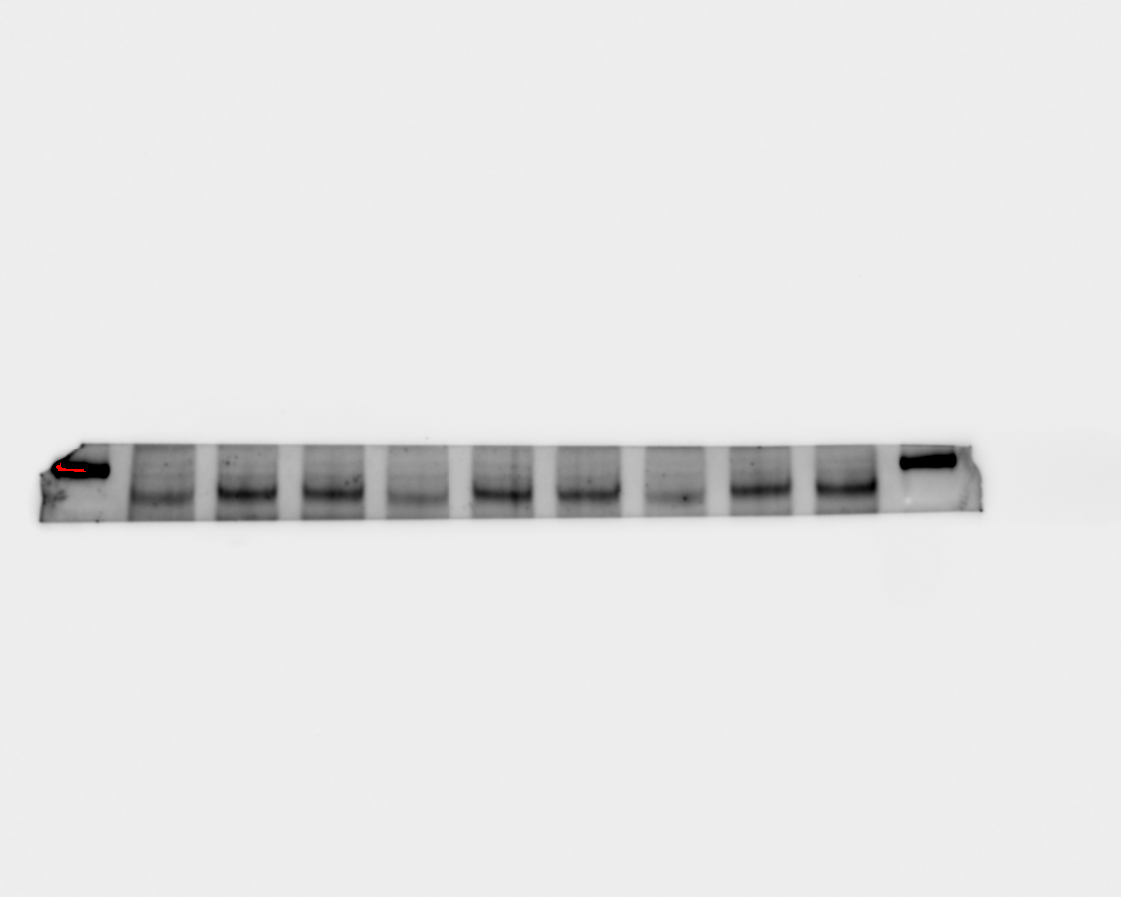

Supplement: Supplementary file 5 [file DataSheet6.ZIP › original data of WB/P-ULK1/P-ULK1.tif]

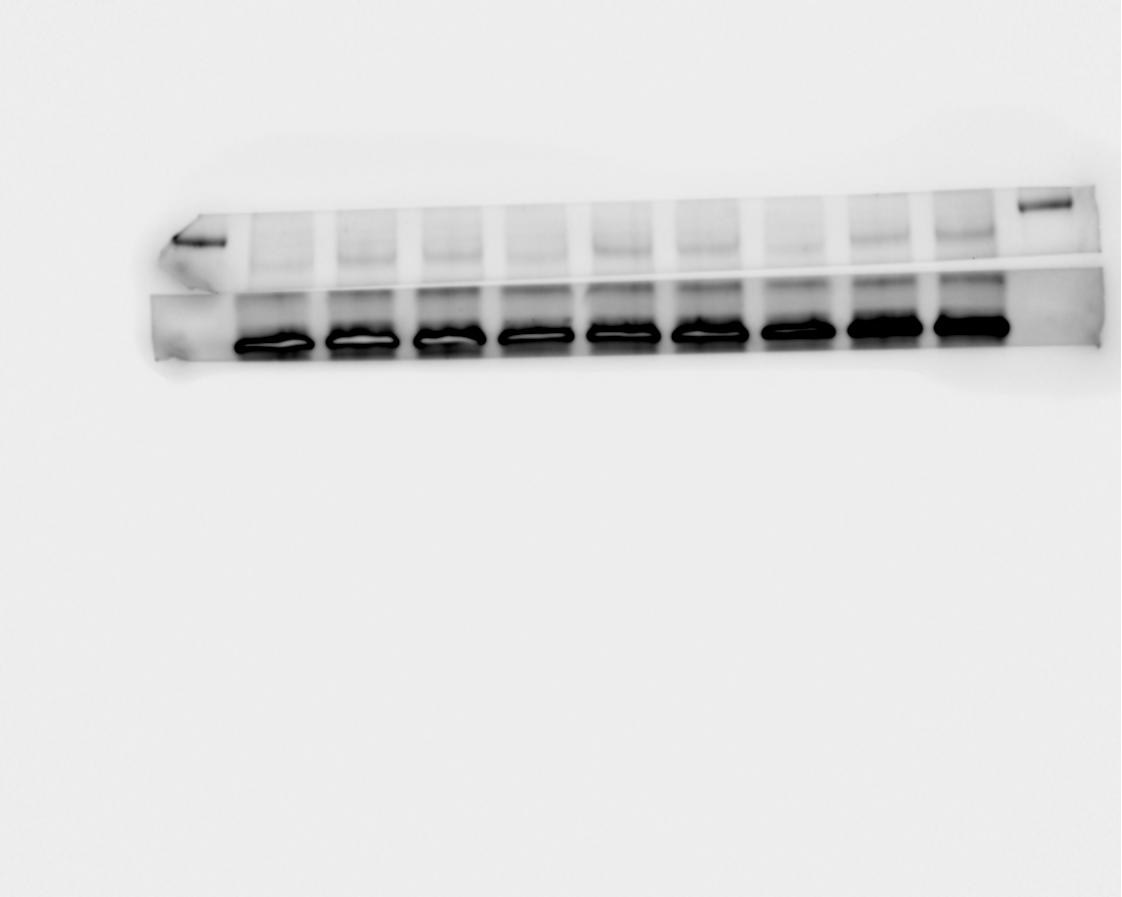

Supplement: Supplementary file 5 [file DataSheet6.ZIP › original data of WB/P-ULK1/P-ULK1+a┬-actin.tif]

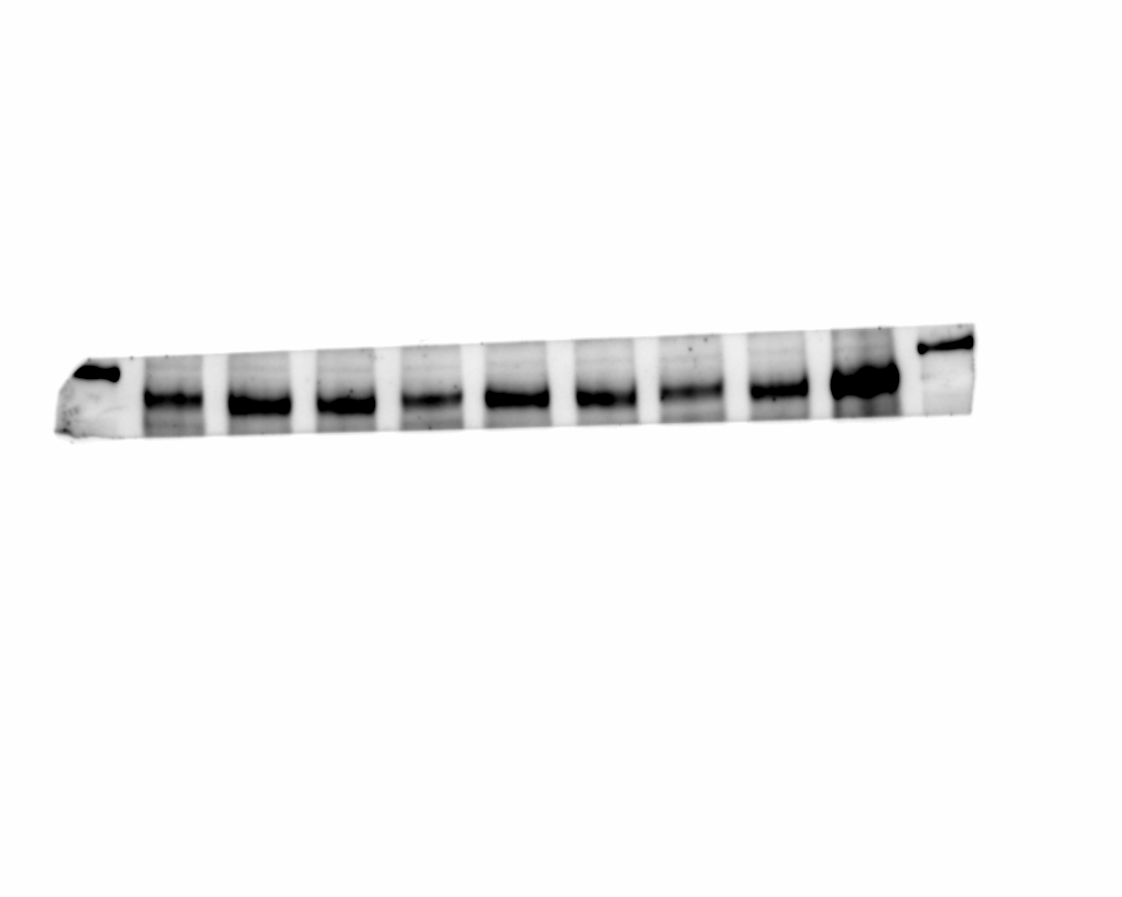

Supplement: Supplementary file 5 [file DataSheet6.ZIP › original data of WB/ULK1/ULK1.tif]

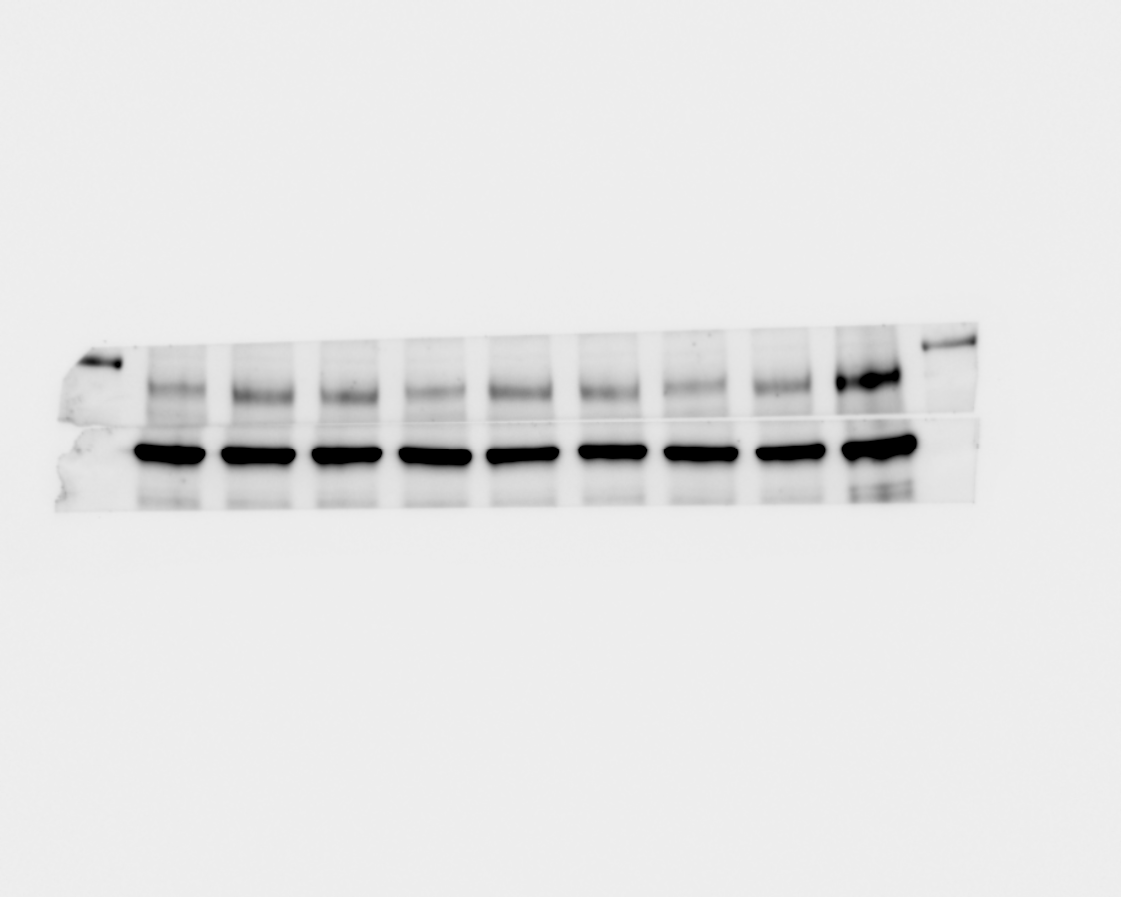

Supplement: Supplementary file 5 [file DataSheet6.ZIP › original data of WB/ULK1/ULK1+a┬-actin.tif]

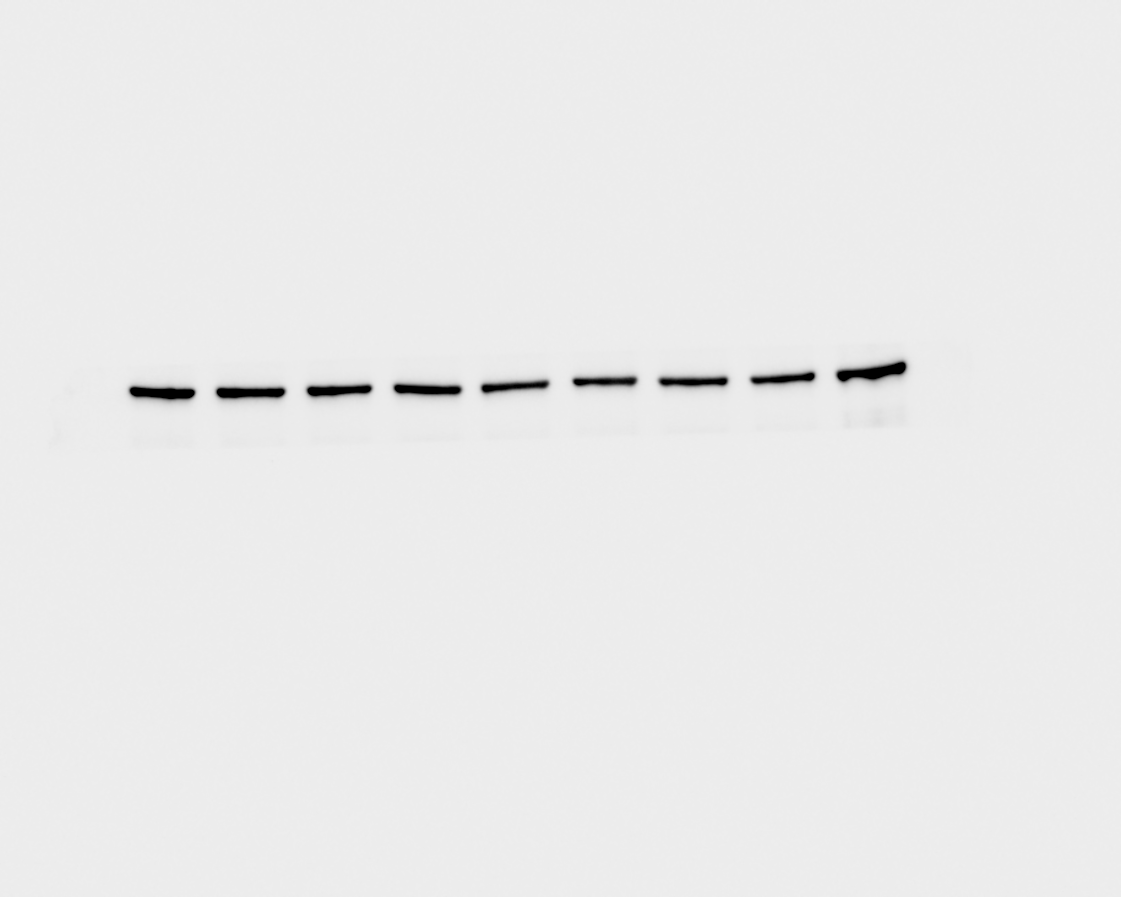

Supplement: Supplementary file 5 [file DataSheet6.ZIP › original data of WB/ULK1/a┬-actin.tif]

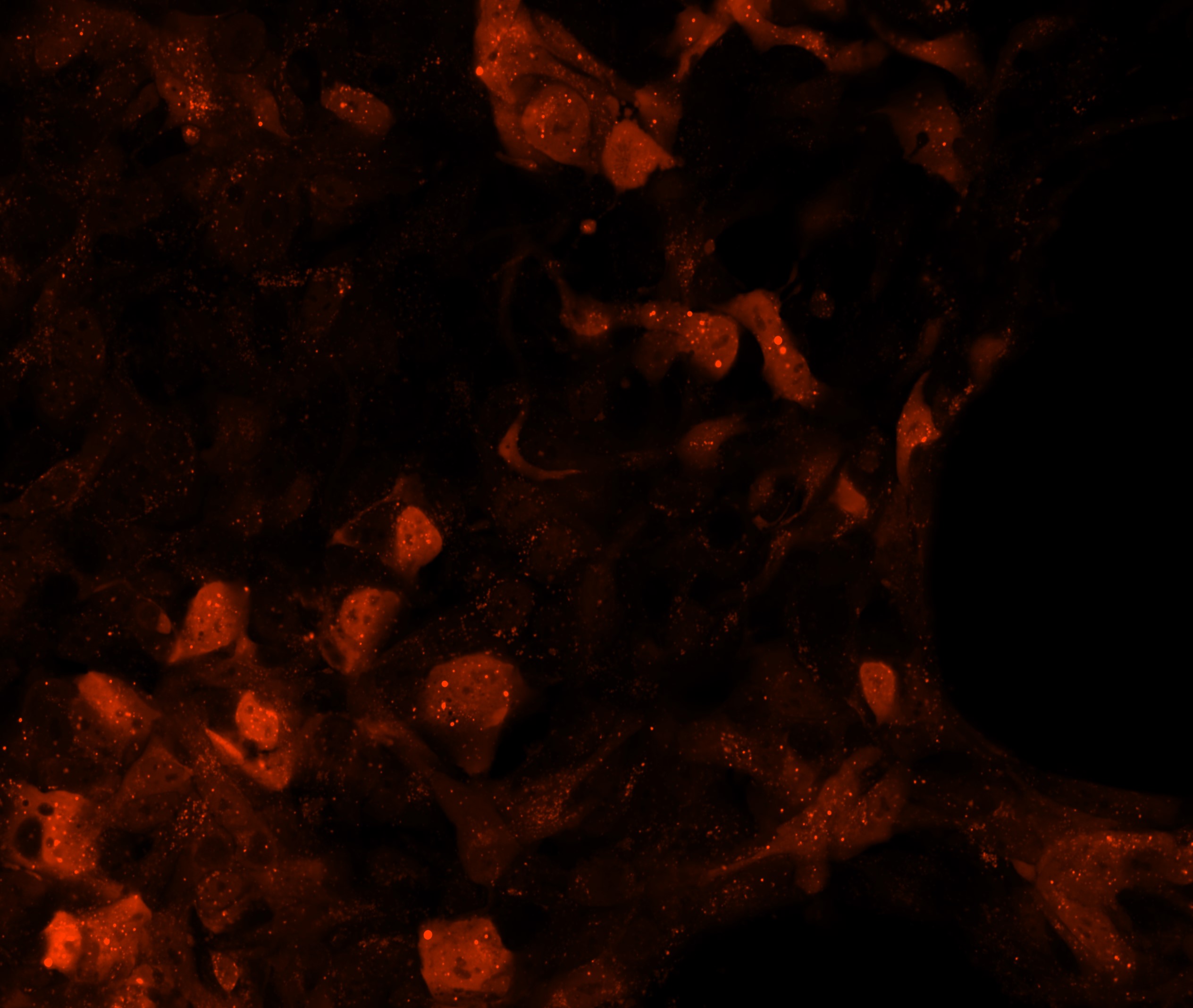

Supplement: Supplementary file 7 [file DataSheet5.ZIP › original data of sensGFP-stubRFP-LC3/FIG.7/250ugml SJC/GFP.jpg]

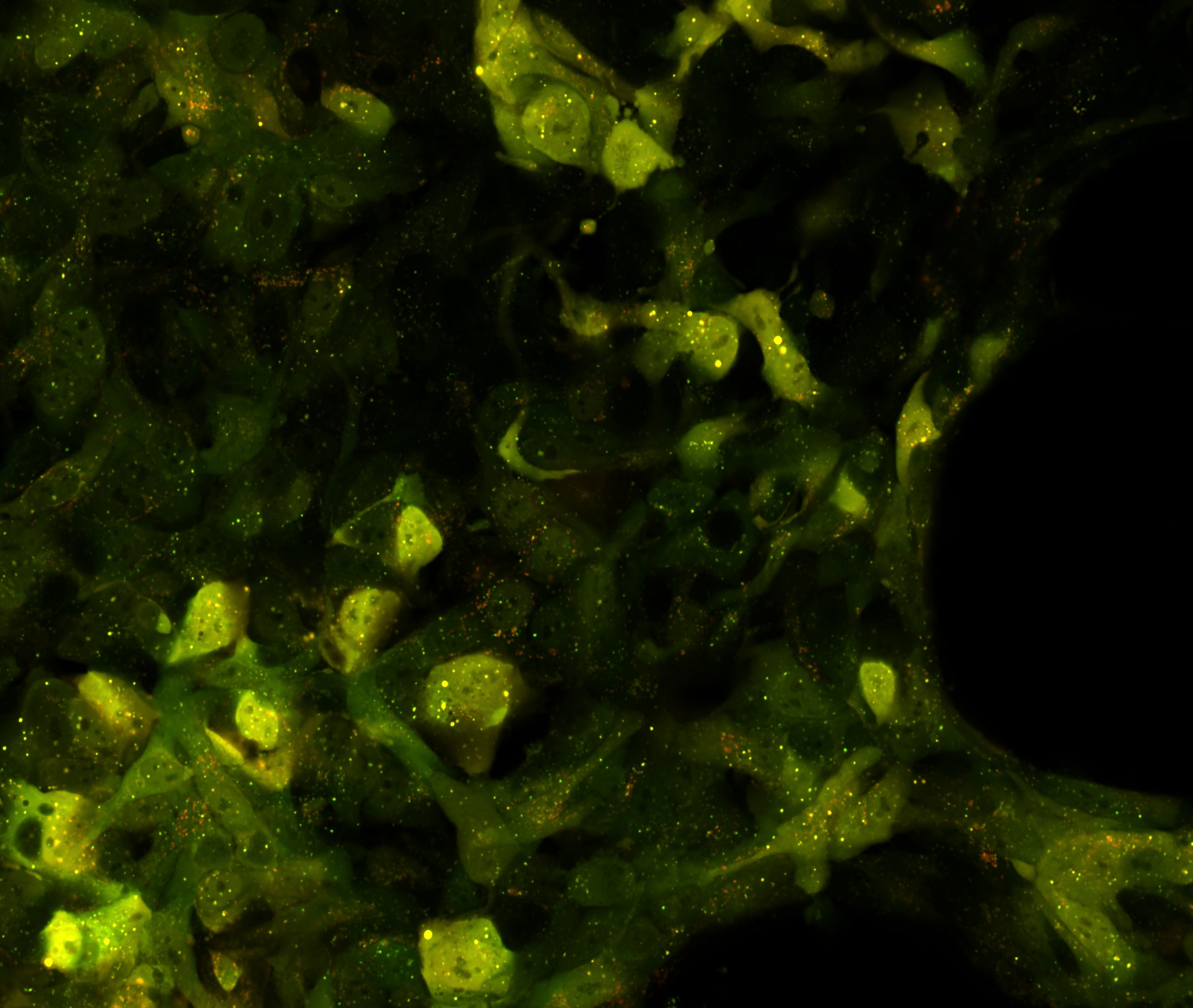

Supplement: Supplementary file 7 [file DataSheet5.ZIP › original data of sensGFP-stubRFP-LC3/FIG.7/250ugml SJC/GFP+RFP.jpg]

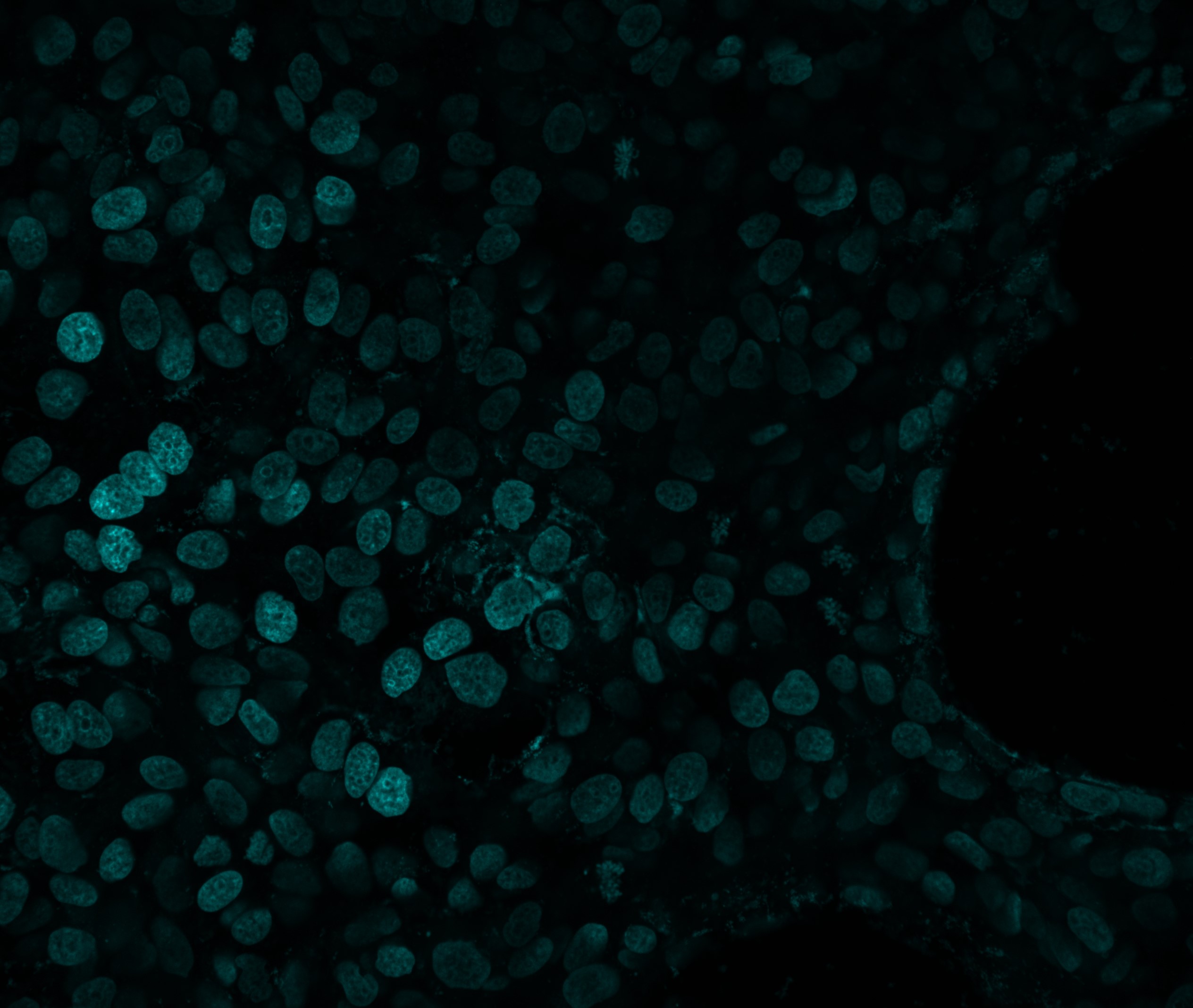

Supplement: Supplementary file 7 [file DataSheet5.ZIP › original data of sensGFP-stubRFP-LC3/FIG.7/250ugml SJC/Hoechst.jpg]

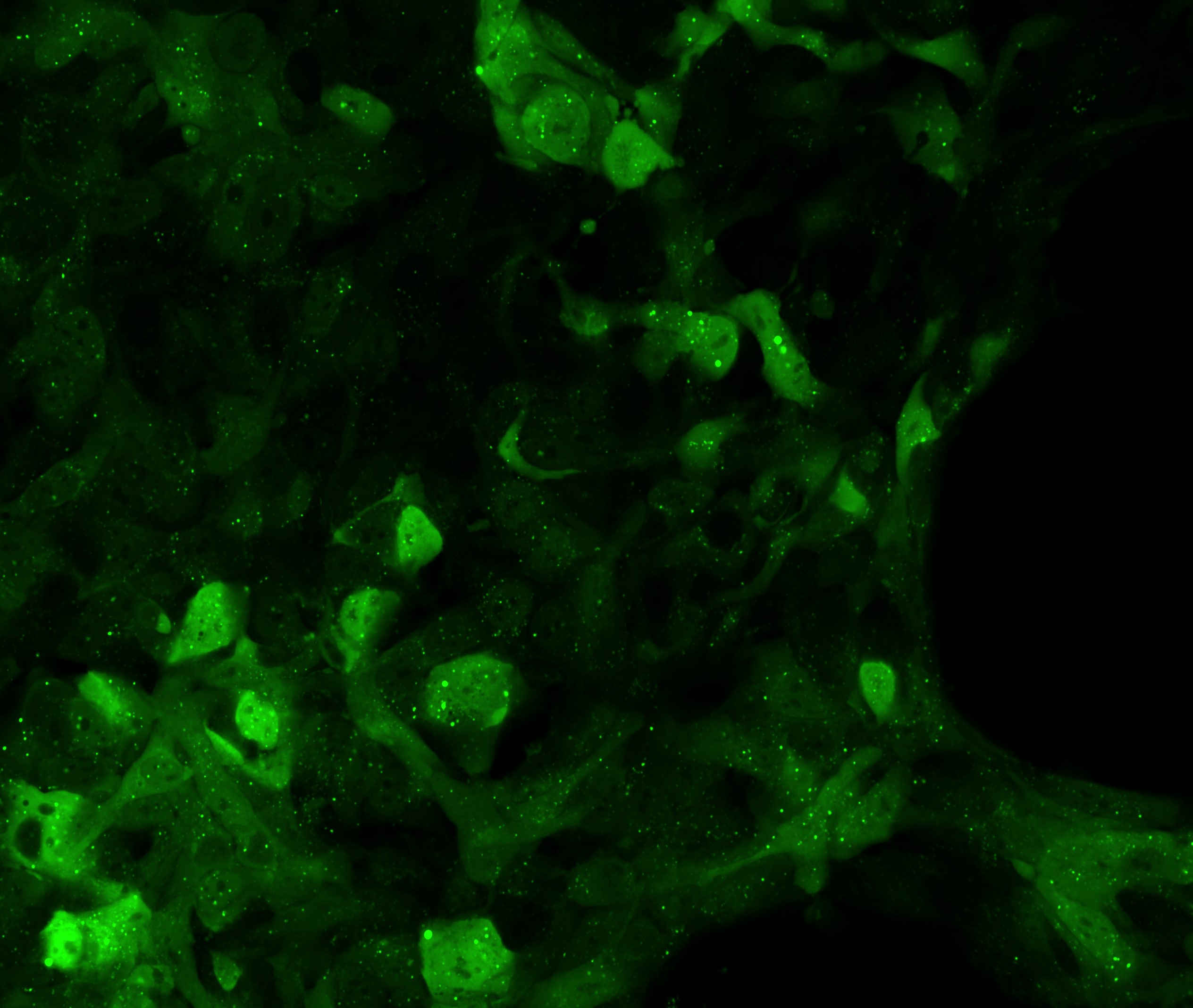

Supplement: Supplementary file 7 [file DataSheet5.ZIP › original data of sensGFP-stubRFP-LC3/FIG.7/250ugml SJC/RFP.jpg]

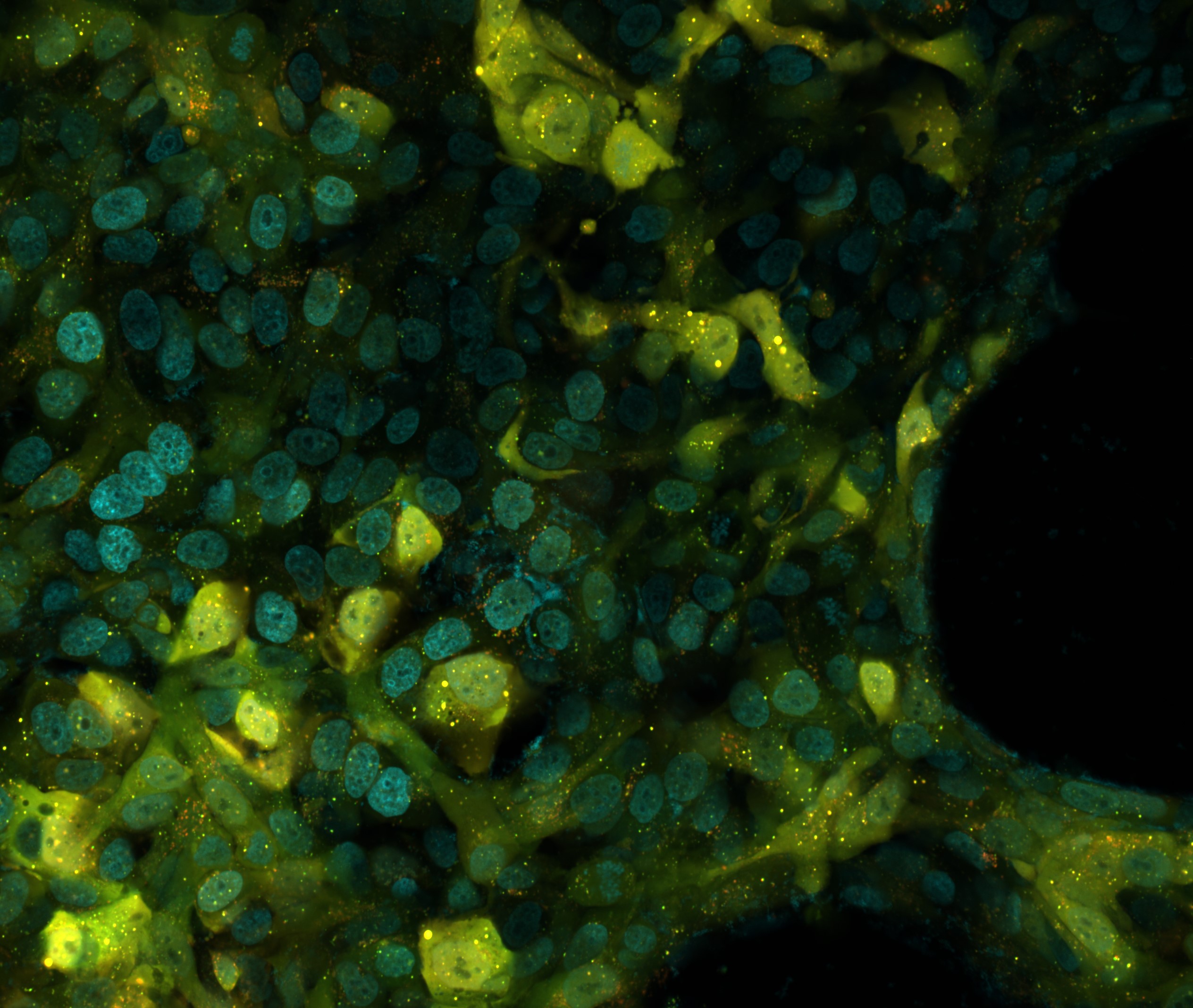

Supplement: Supplementary file 7 [file DataSheet5.ZIP › original data of sensGFP-stubRFP-LC3/FIG.7/250ugml SJC/RFP+GFP+Hoechst.jpg]

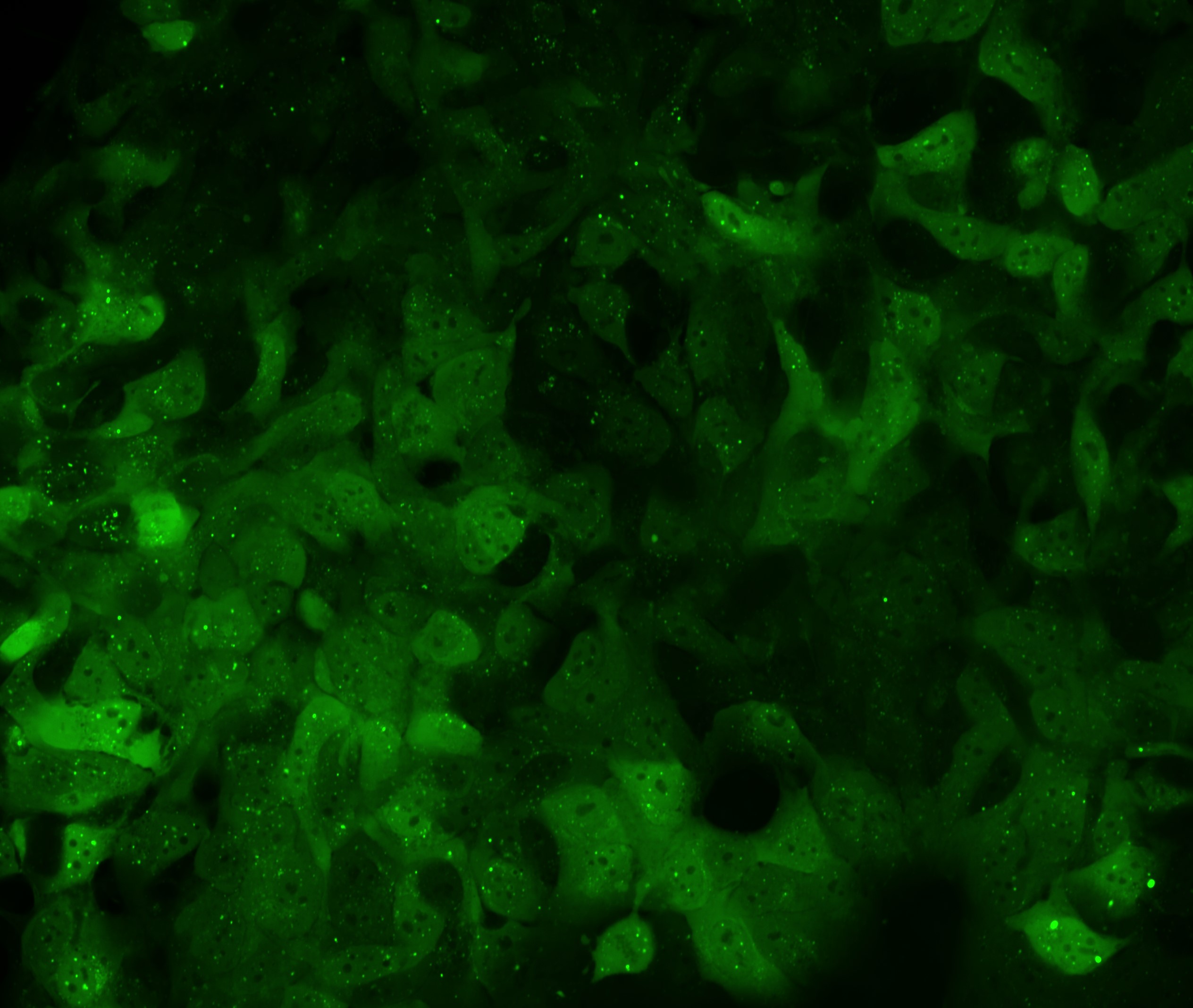

Supplement: Supplementary file 7 [file DataSheet5.ZIP › original data of sensGFP-stubRFP-LC3/FIG.7/500ugml SJC/GFP.jpg]

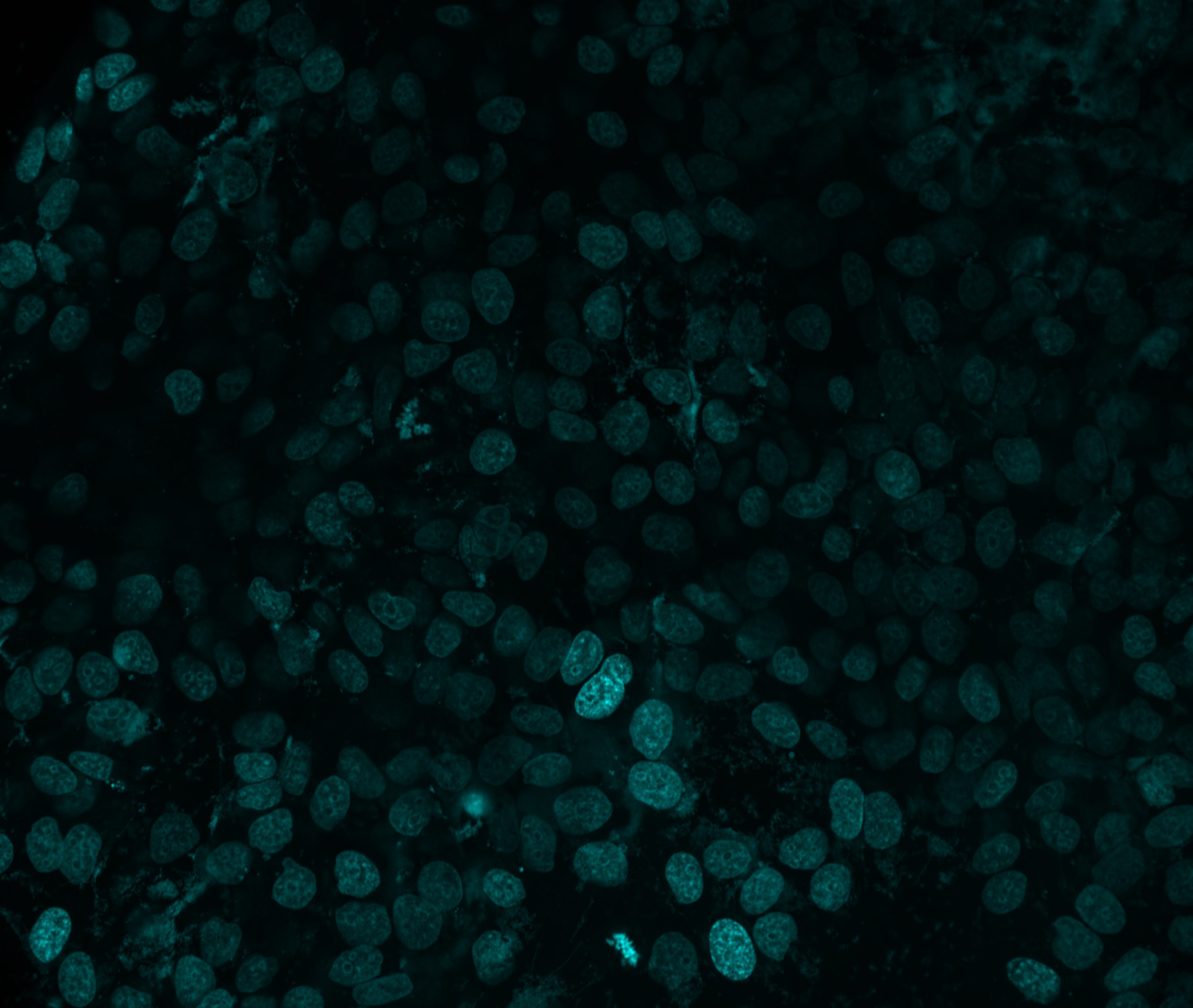

Supplement: Supplementary file 7 [file DataSheet5.ZIP › original data of sensGFP-stubRFP-LC3/FIG.7/500ugml SJC/Hoechst.jpg]

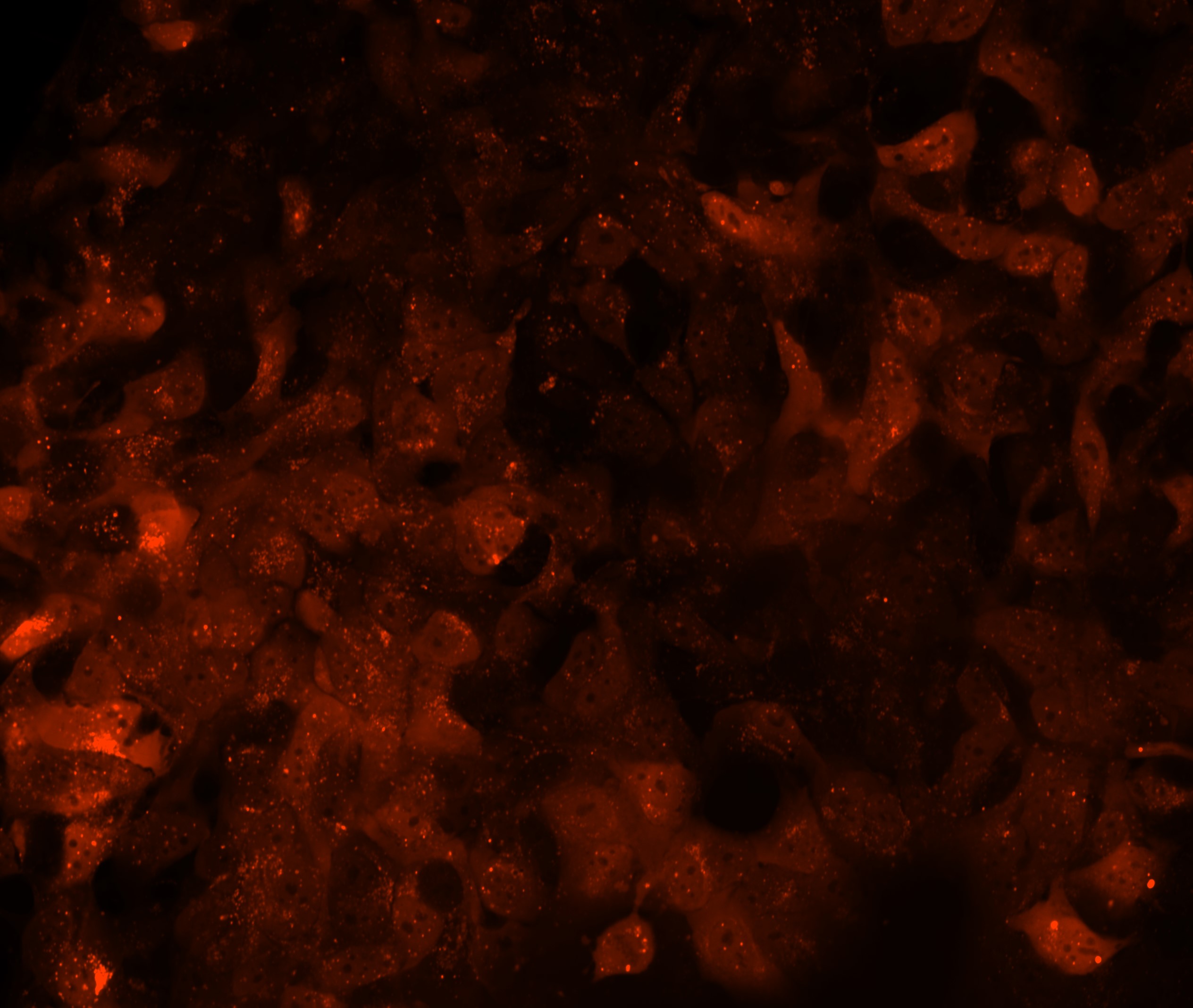

Supplement: Supplementary file 7 [file DataSheet5.ZIP › original data of sensGFP-stubRFP-LC3/FIG.7/500ugml SJC/RFP.jpg]

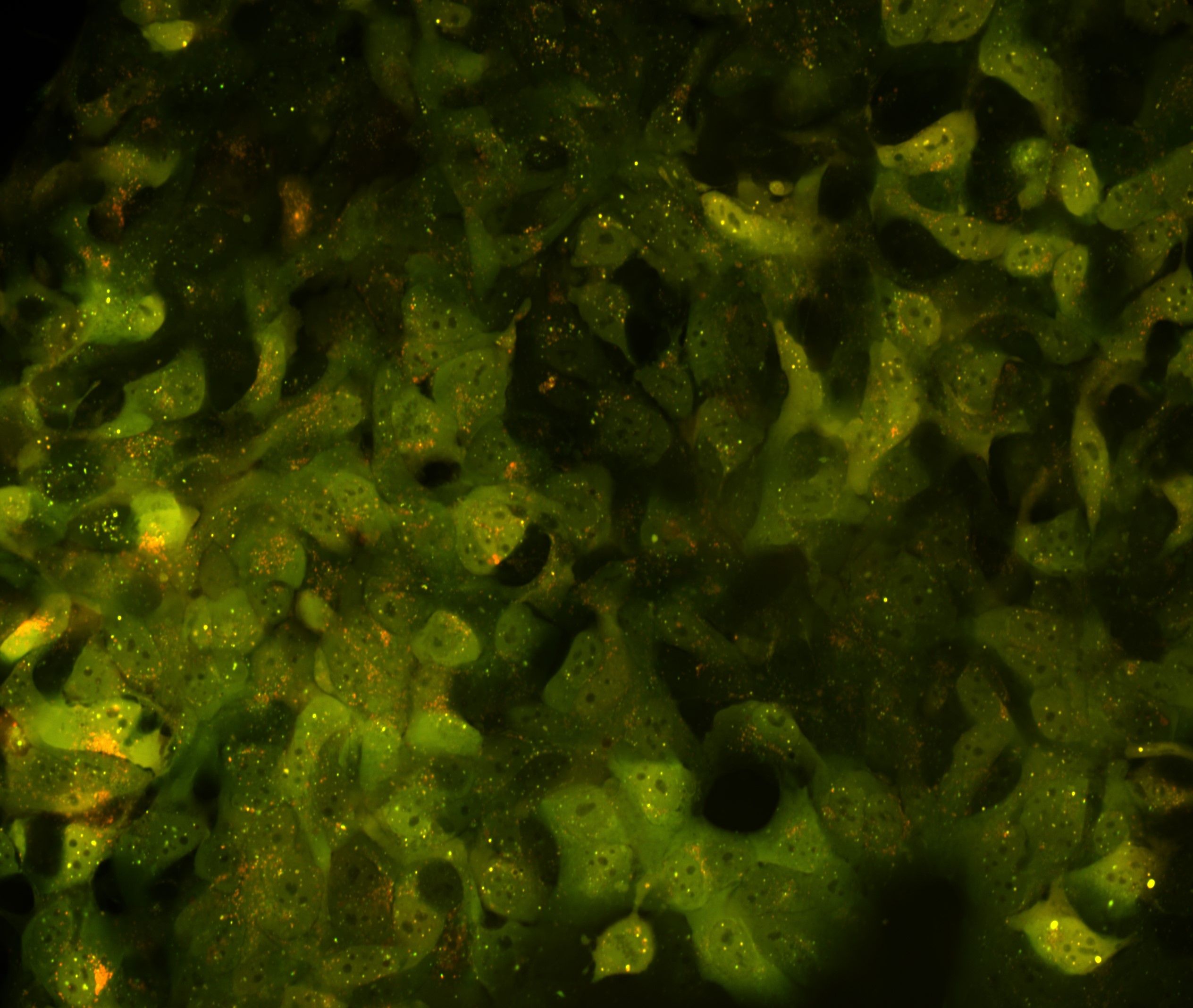

Supplement: Supplementary file 7 [file DataSheet5.ZIP › original data of sensGFP-stubRFP-LC3/FIG.7/500ugml SJC/RFP+GFP.jpg]

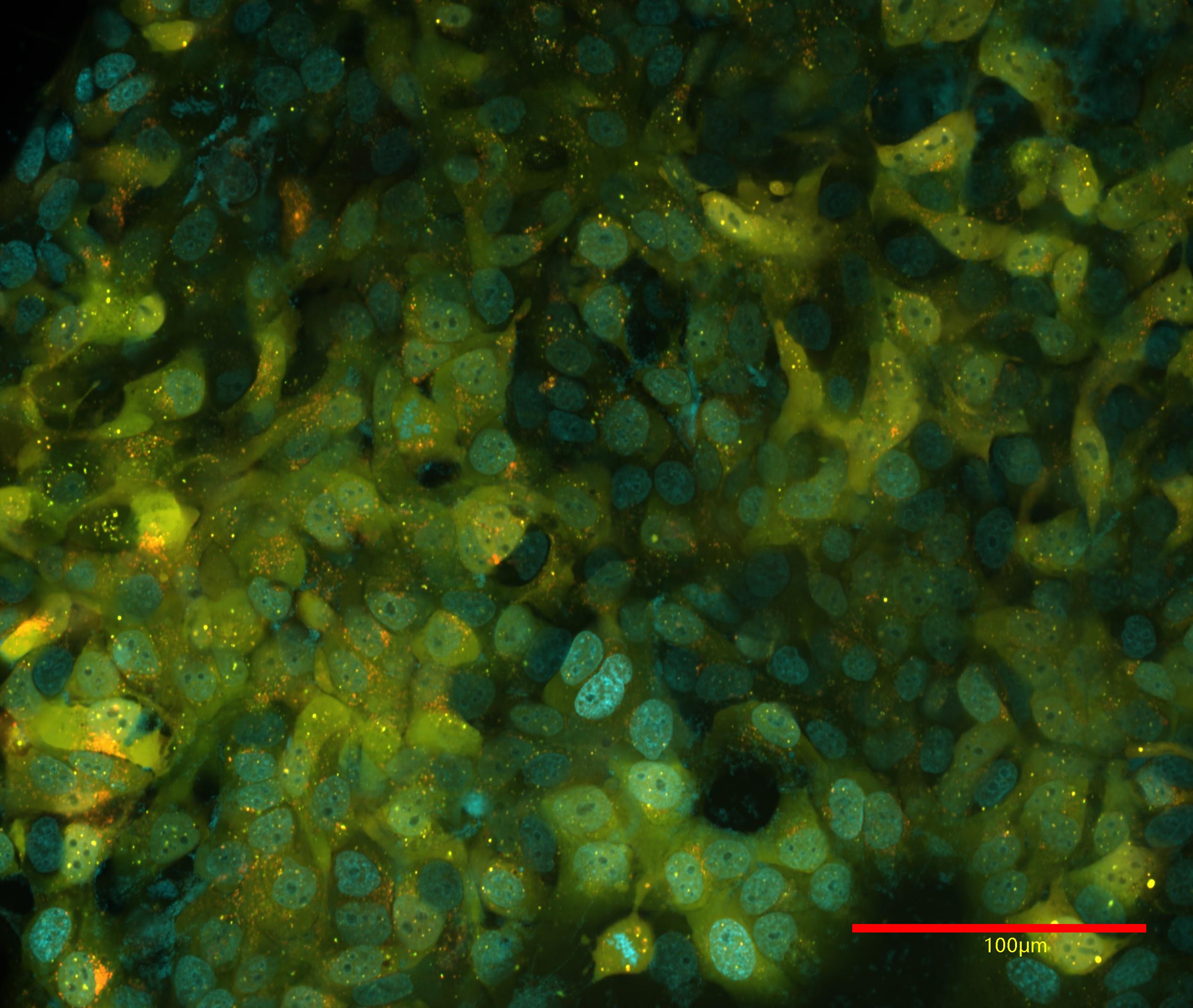

Supplement: Supplementary file 7 [file DataSheet5.ZIP › original data of sensGFP-stubRFP-LC3/FIG.7/500ugml SJC/RFP+GFP+Hoechst.jpg]

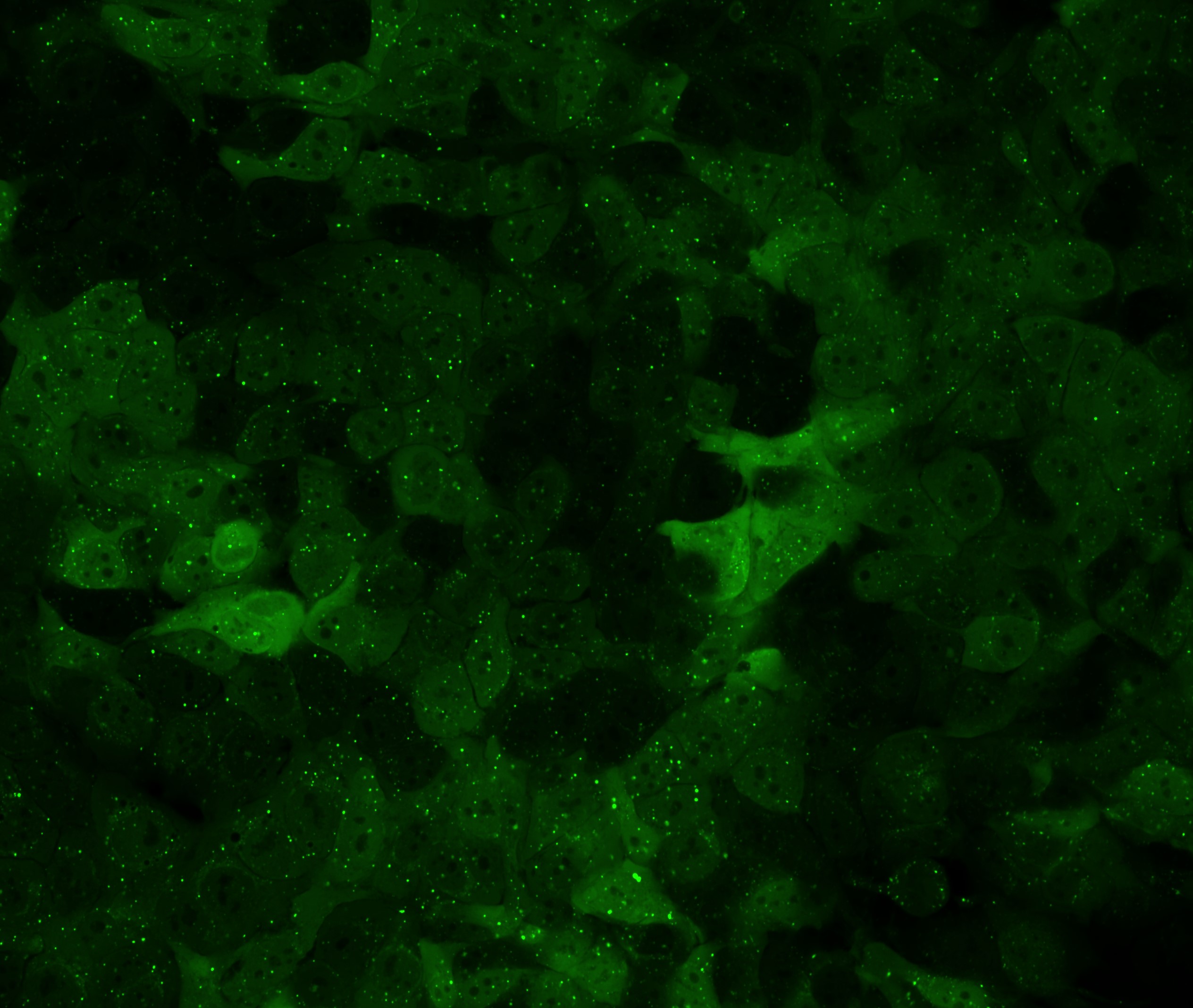

Supplement: Supplementary file 7 [file DataSheet5.ZIP › original data of sensGFP-stubRFP-LC3/FIG.7/control/GFP.jpg]

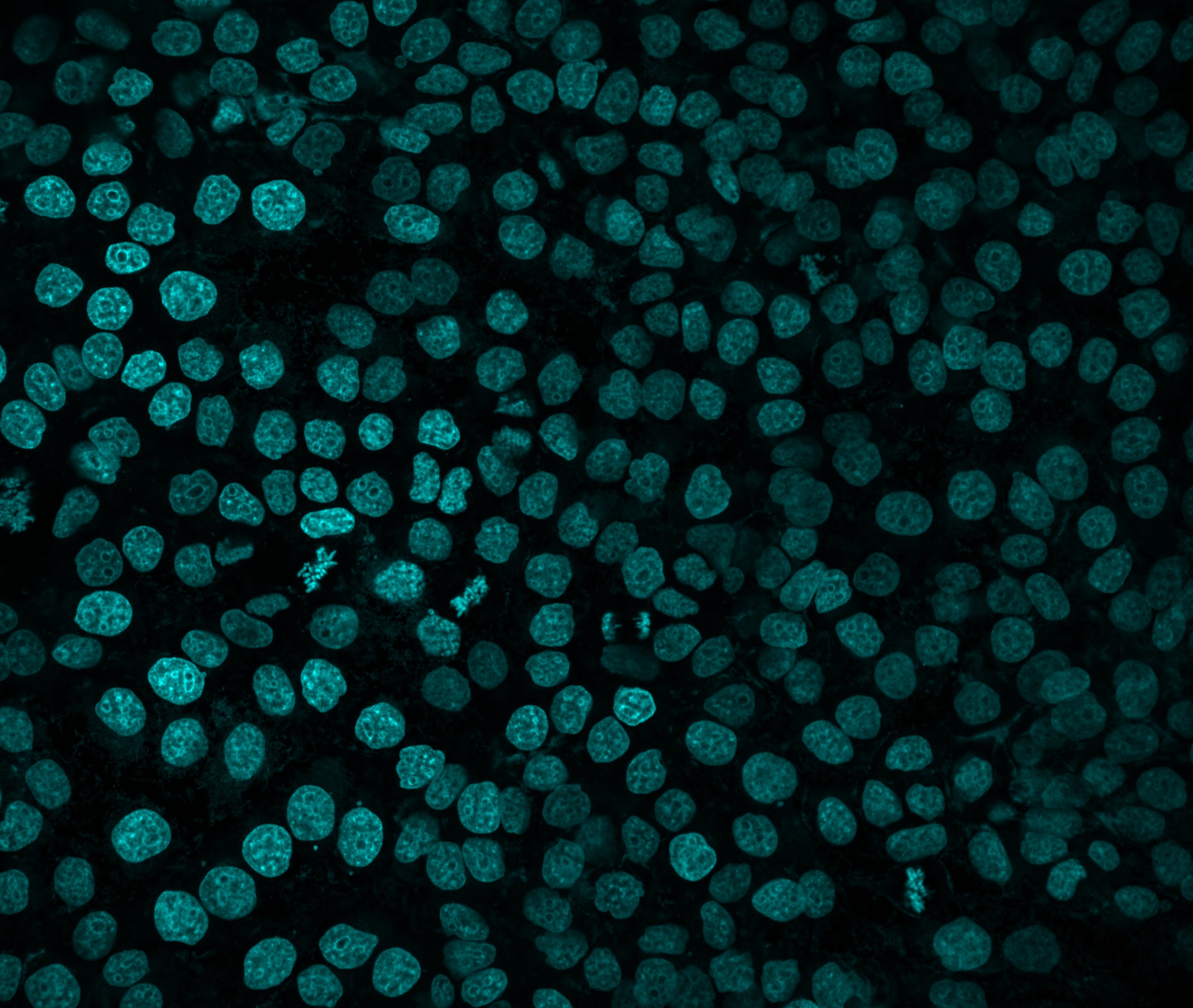

Supplement: Supplementary file 7 [file DataSheet5.ZIP › original data of sensGFP-stubRFP-LC3/FIG.7/control/Hoechst.jpg]

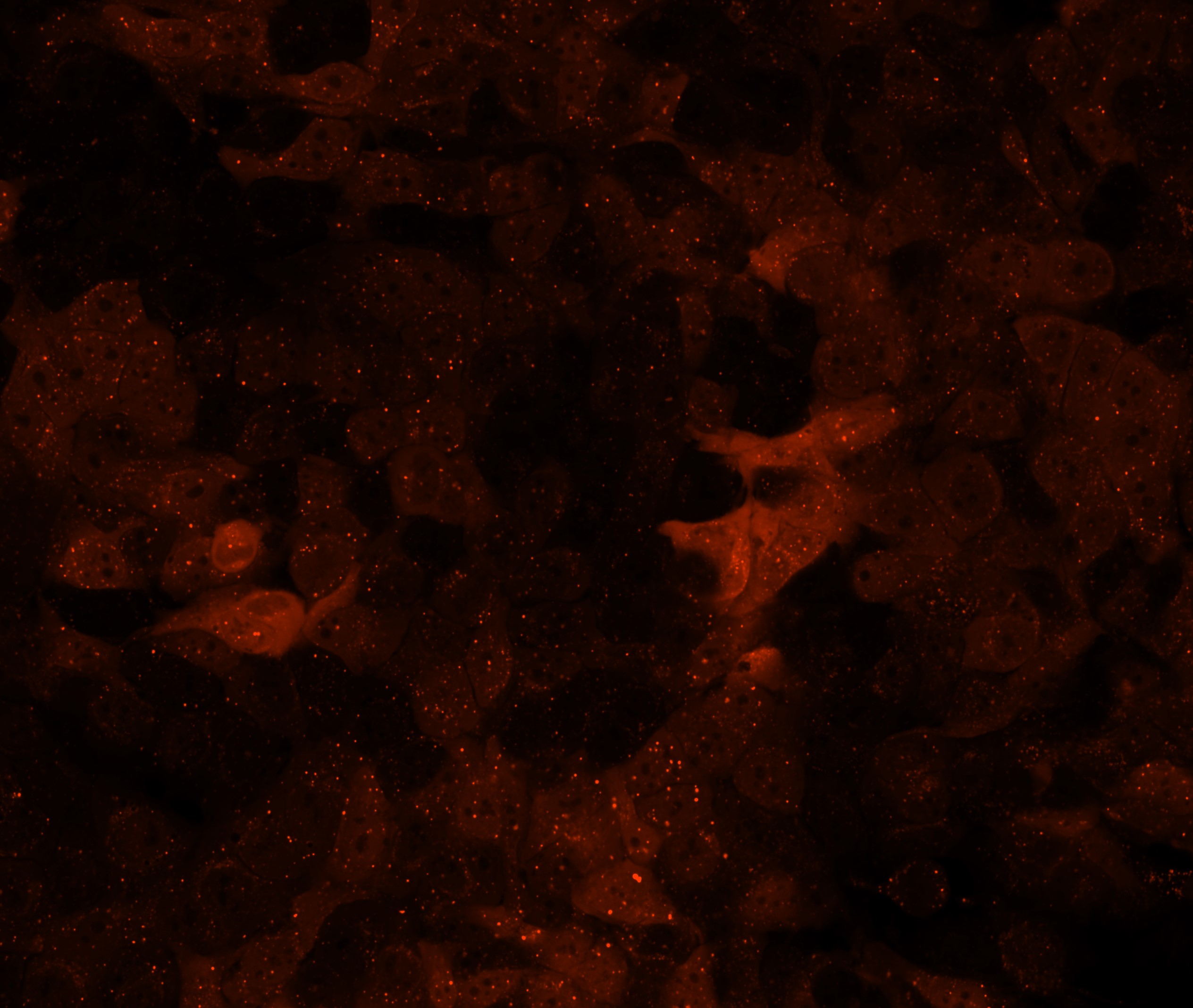

Supplement: Supplementary file 7 [file DataSheet5.ZIP › original data of sensGFP-stubRFP-LC3/FIG.7/control/RFP.jpg]

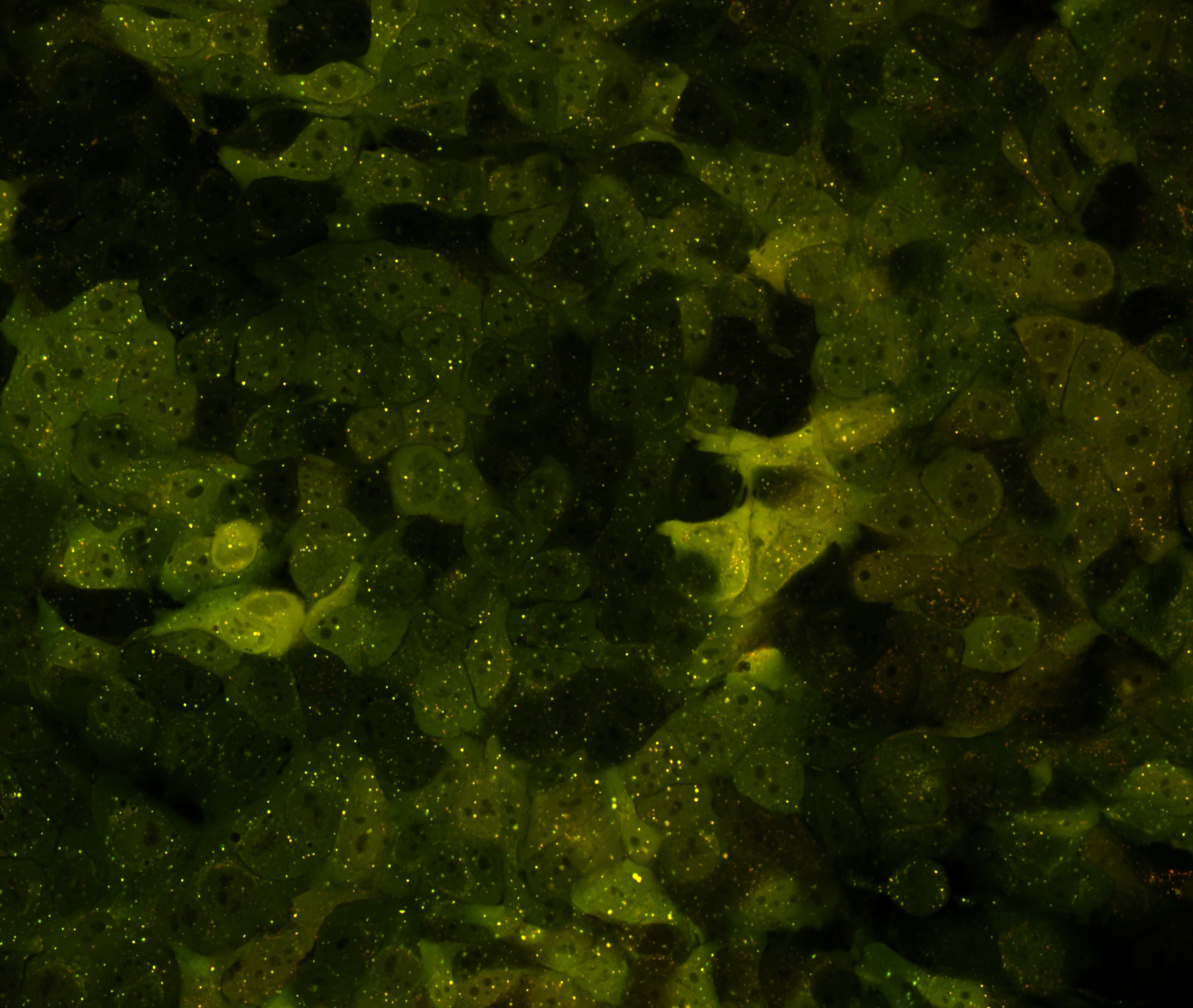

Supplement: Supplementary file 7 [file DataSheet5.ZIP › original data of sensGFP-stubRFP-LC3/FIG.7/control/RFP+GFP.jpg]

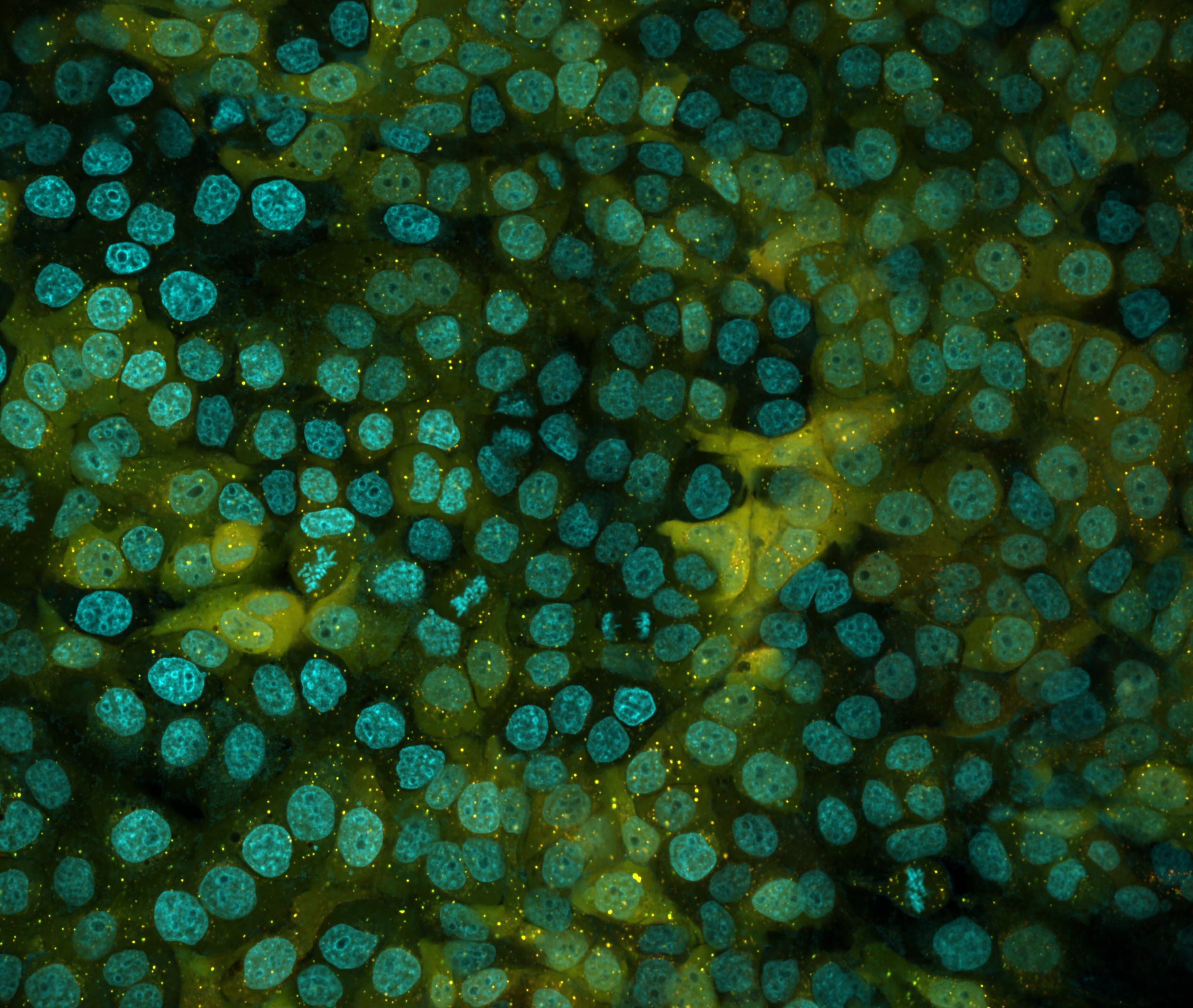

Supplement: Supplementary file 7 [file DataSheet5.ZIP › original data of sensGFP-stubRFP-LC3/FIG.7/control/RFP+GFP+Hoechst.jpg]

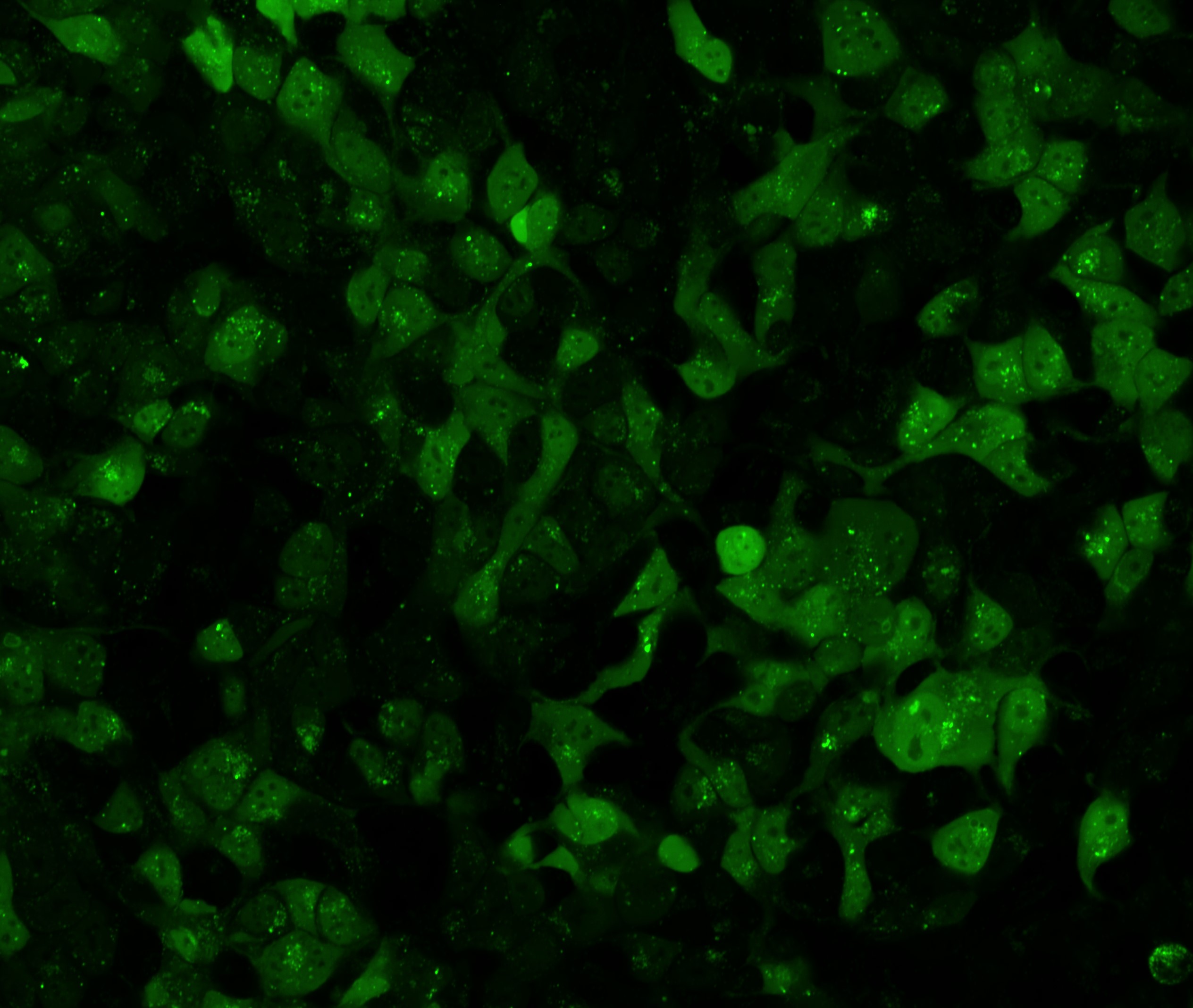

Supplement: Supplementary file 7 [file DataSheet5.ZIP › original data of sensGFP-stubRFP-LC3/FIG.7/Rapa/GFP.jpg]

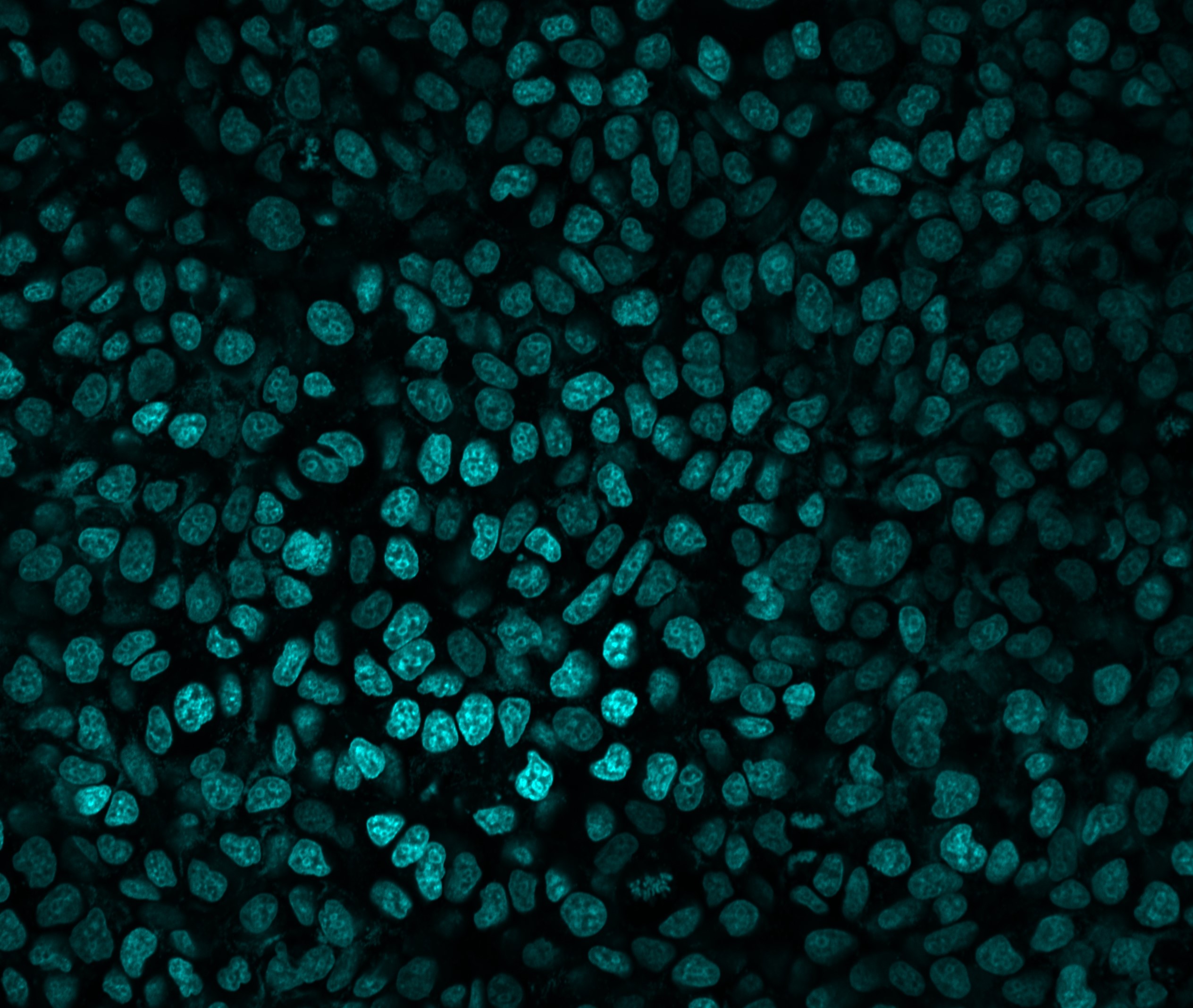

Supplement: Supplementary file 7 [file DataSheet5.ZIP › original data of sensGFP-stubRFP-LC3/FIG.7/Rapa/Hoechst.jpg]

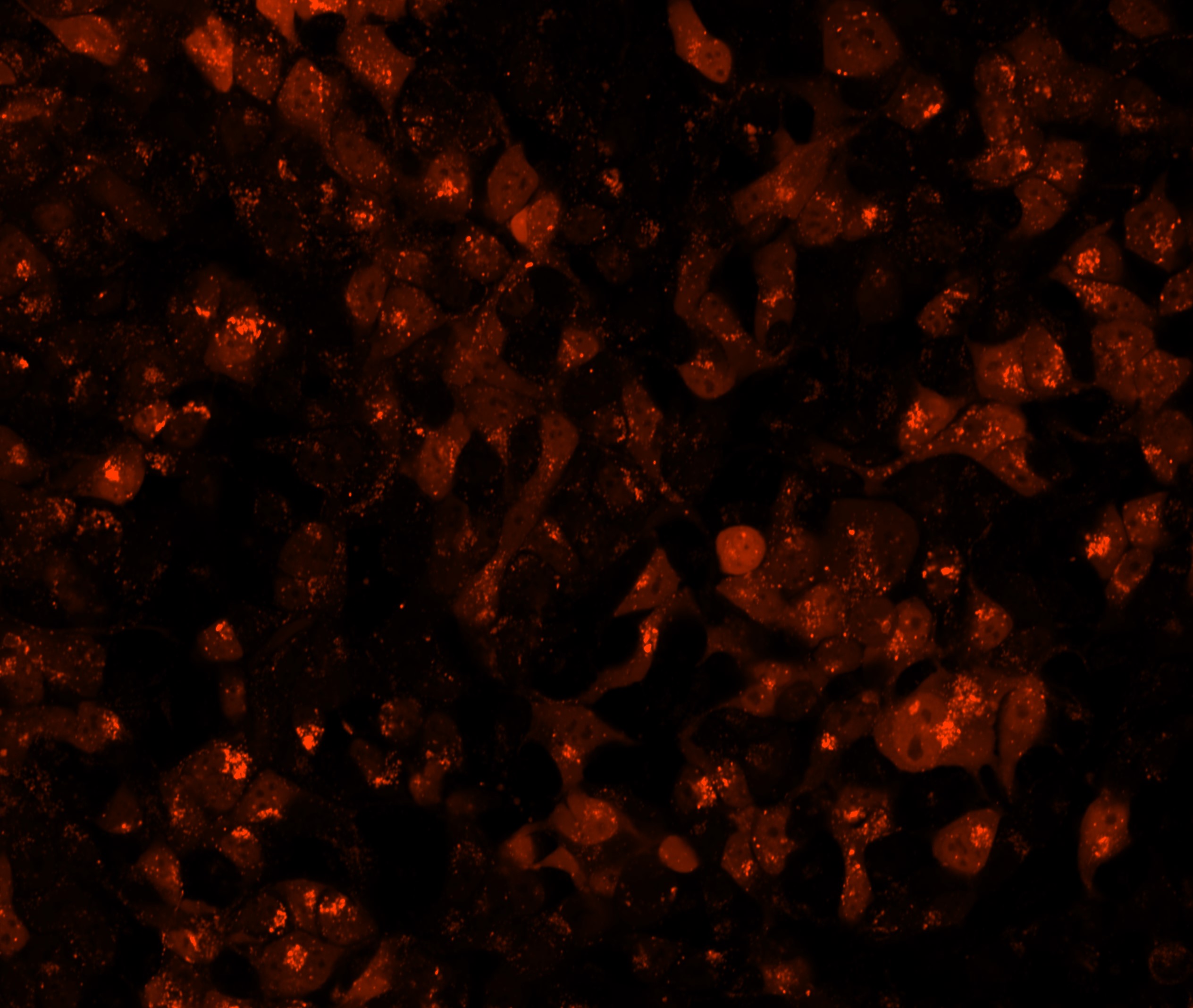

Supplement: Supplementary file 7 [file DataSheet5.ZIP › original data of sensGFP-stubRFP-LC3/FIG.7/Rapa/RFP.jpg]

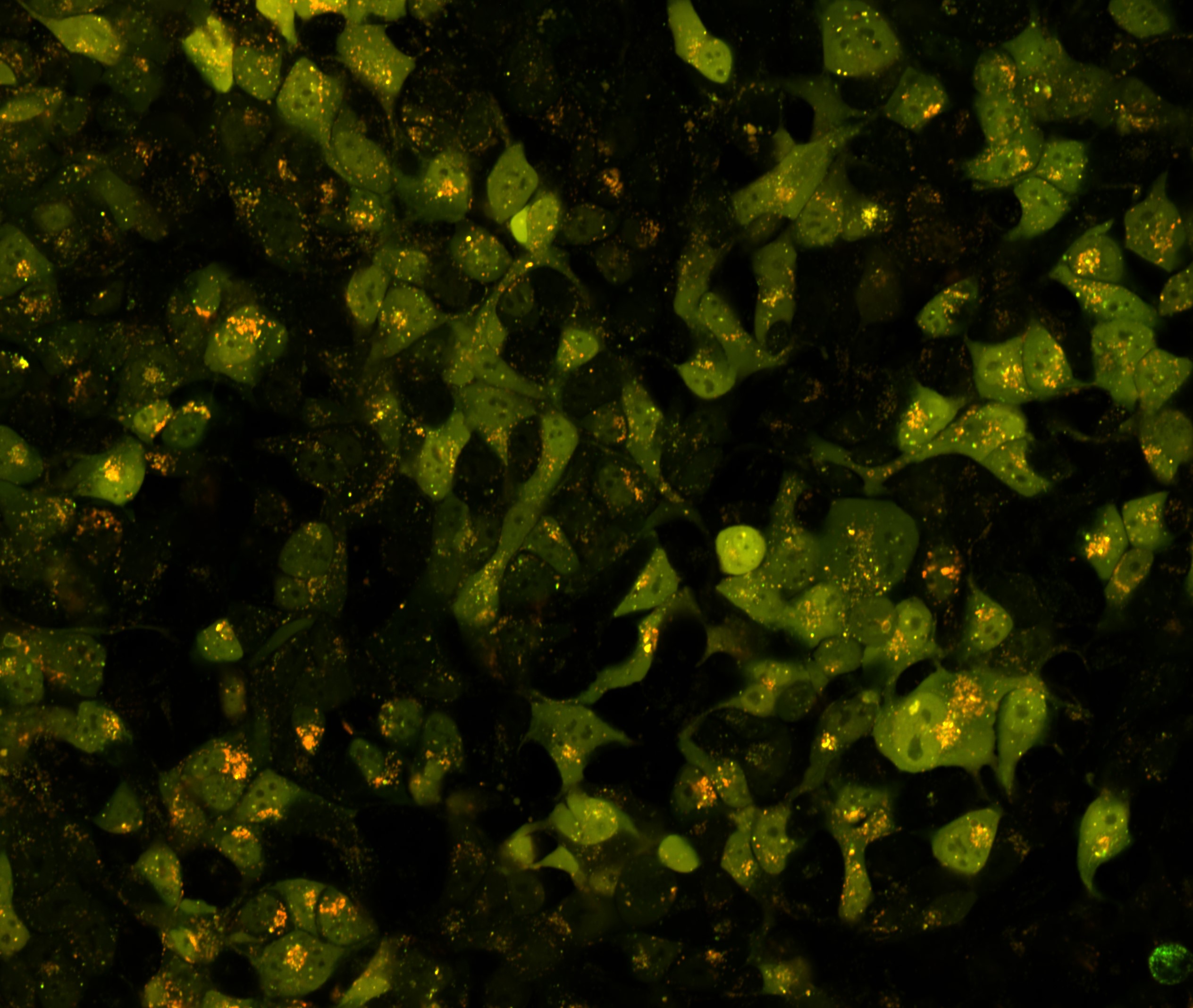

Supplement: Supplementary file 7 [file DataSheet5.ZIP › original data of sensGFP-stubRFP-LC3/FIG.7/Rapa/RFP+GFP.jpg]

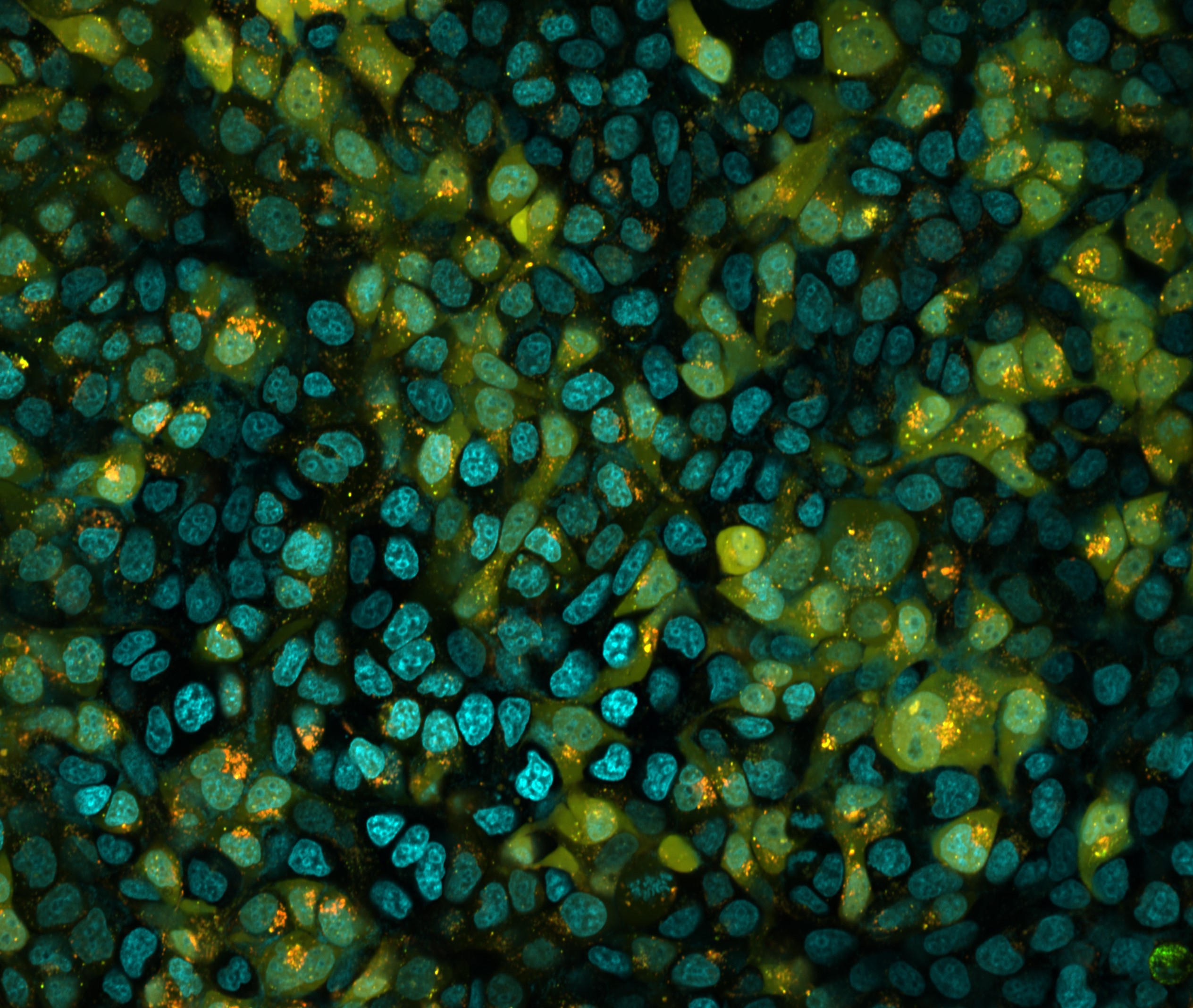

Supplement: Supplementary file 7 [file DataSheet5.ZIP › original data of sensGFP-stubRFP-LC3/FIG.7/Rapa/RFP+GFP+Hoechst.jpg]

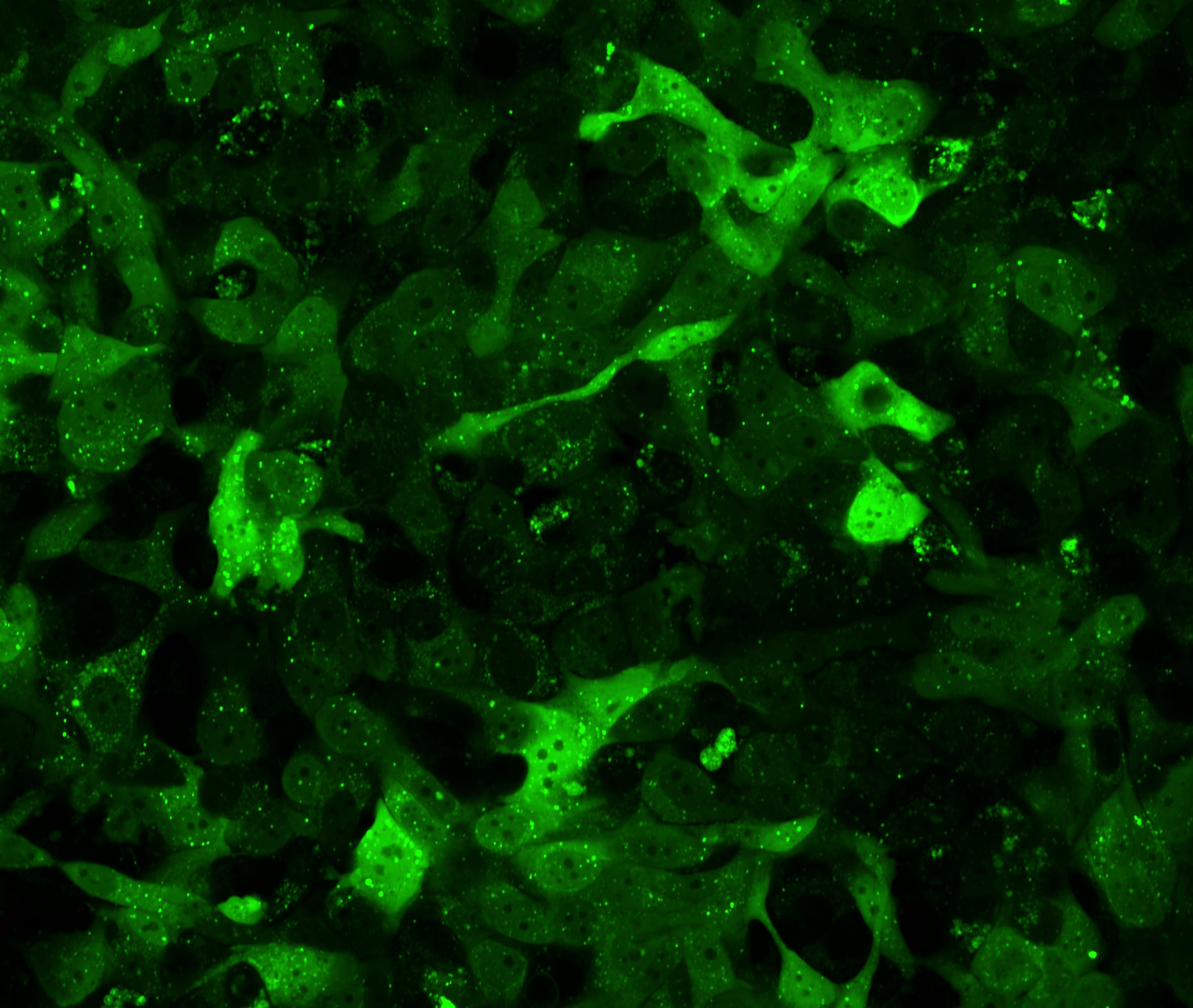

Supplement: Supplementary file 7 [file DataSheet5.ZIP › original data of sensGFP-stubRFP-LC3/FIG.8/500ugml SJC+CQ/GFP.jpg]

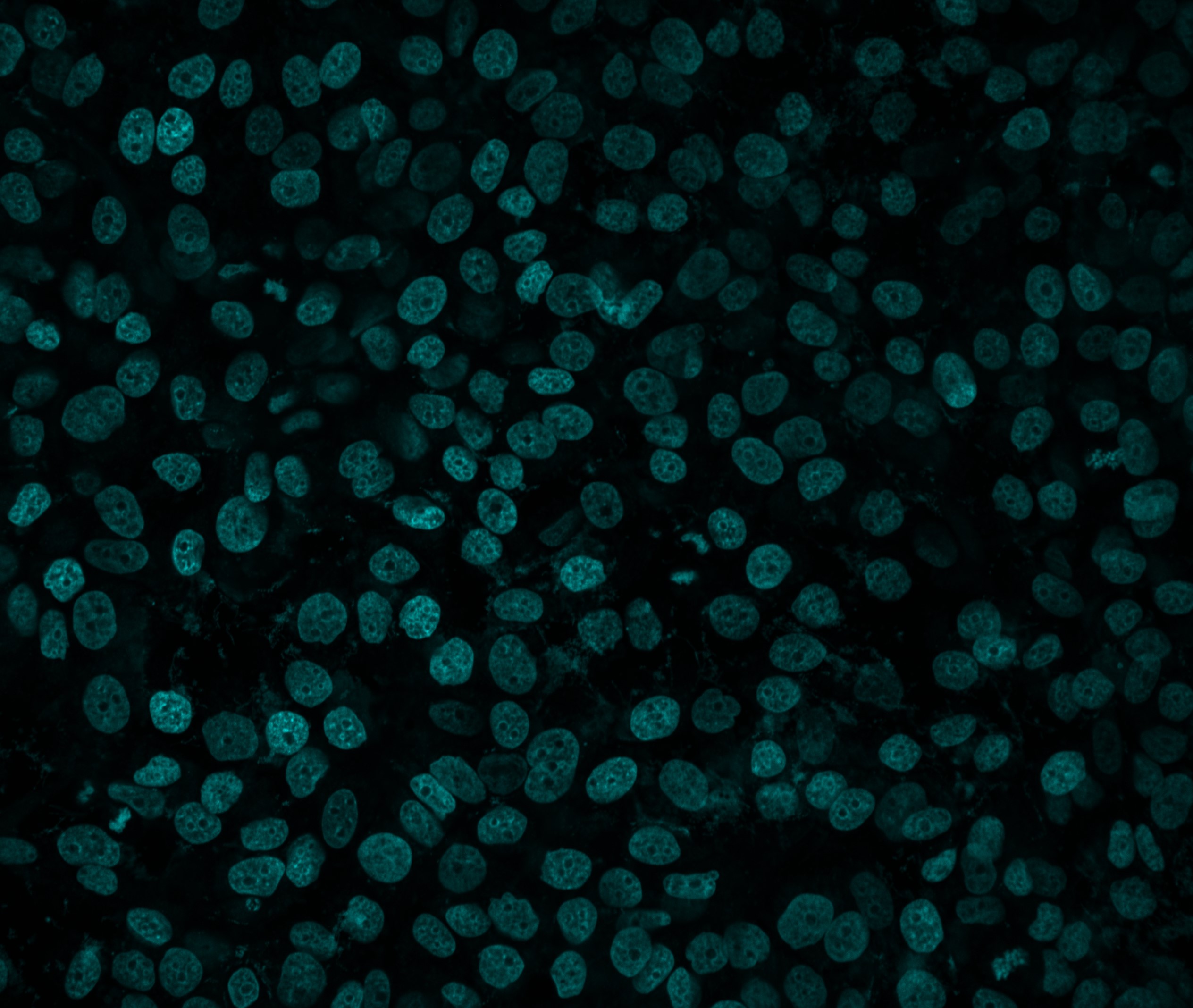

Supplement: Supplementary file 7 [file DataSheet5.ZIP › original data of sensGFP-stubRFP-LC3/FIG.8/500ugml SJC+CQ/Hoechst.jpg]

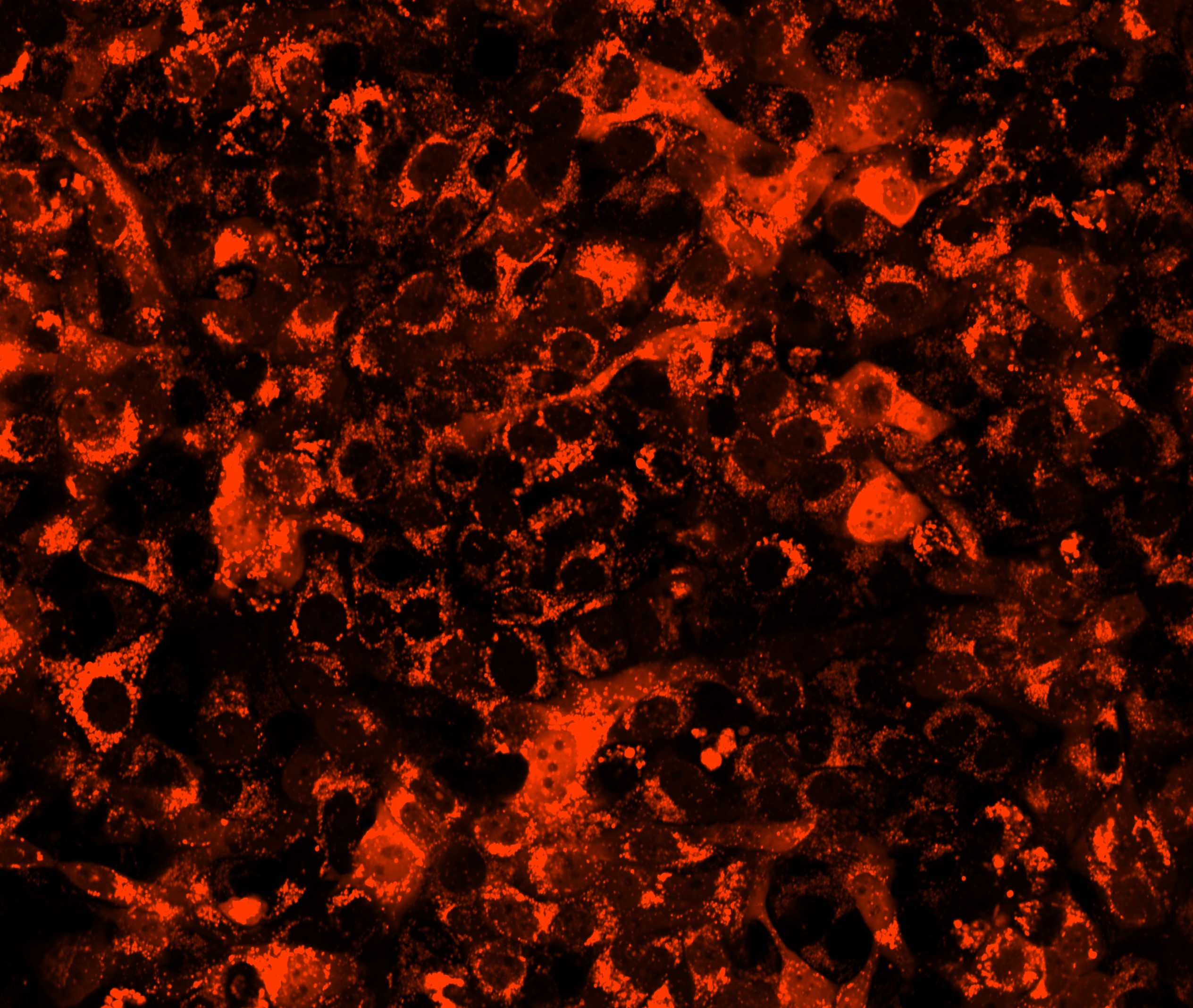

Supplement: Supplementary file 7 [file DataSheet5.ZIP › original data of sensGFP-stubRFP-LC3/FIG.8/500ugml SJC+CQ/RFP.jpg]

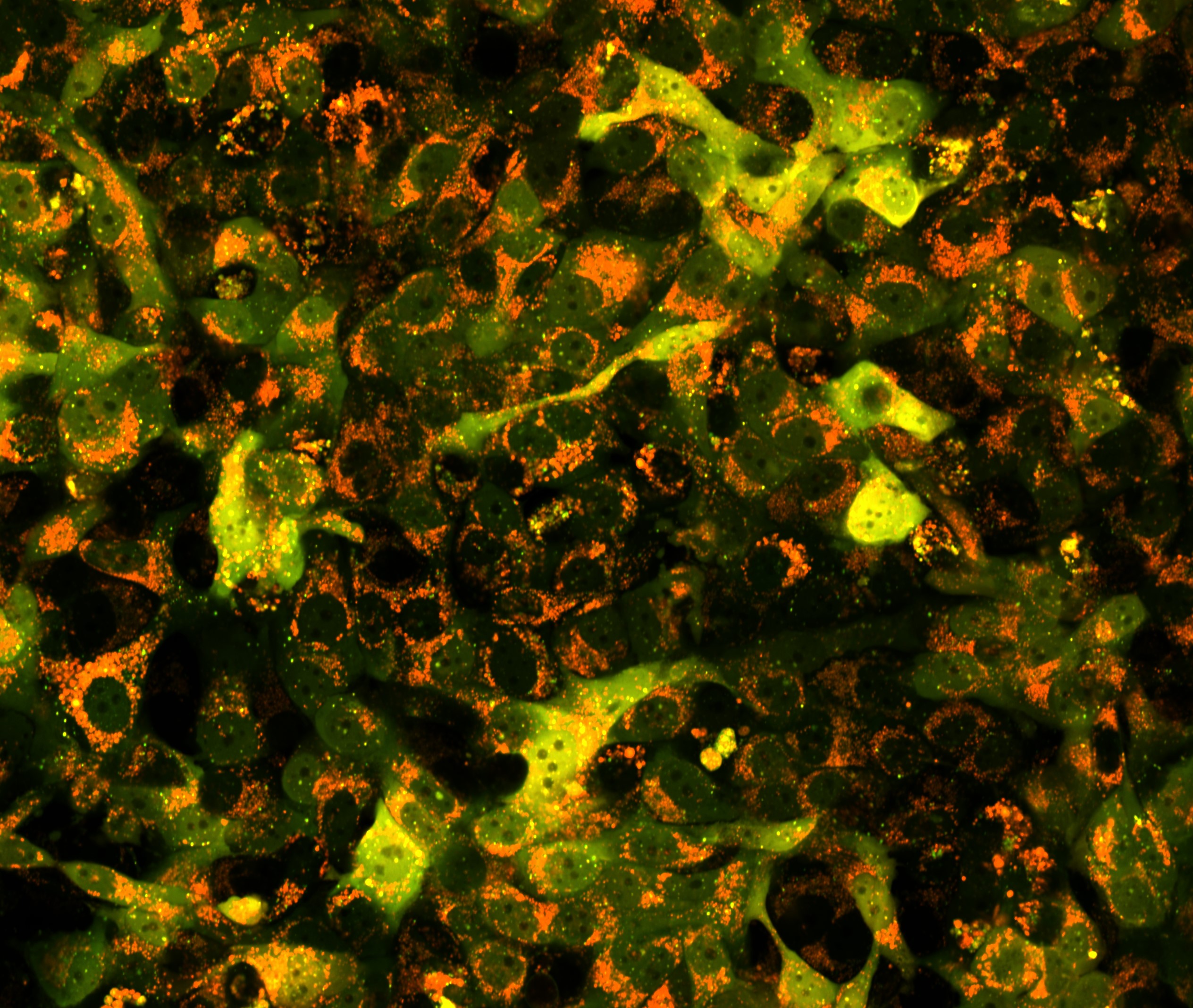

Supplement: Supplementary file 7 [file DataSheet5.ZIP › original data of sensGFP-stubRFP-LC3/FIG.8/500ugml SJC+CQ/RFP+GFP.jpg]

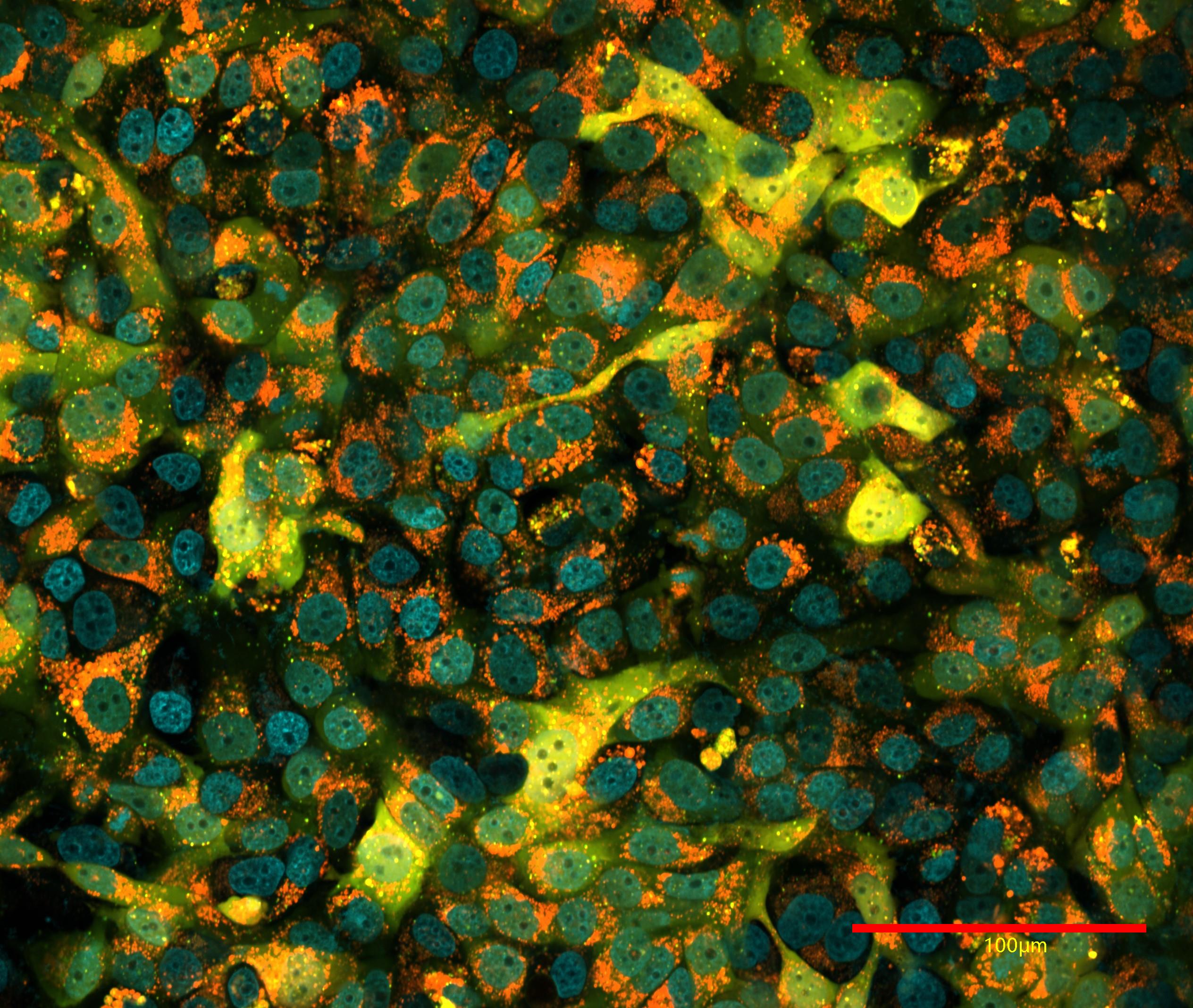

Supplement: Supplementary file 7 [file DataSheet5.ZIP › original data of sensGFP-stubRFP-LC3/FIG.8/500ugml SJC+CQ/RFP+GFP+Hoechst.jpg]

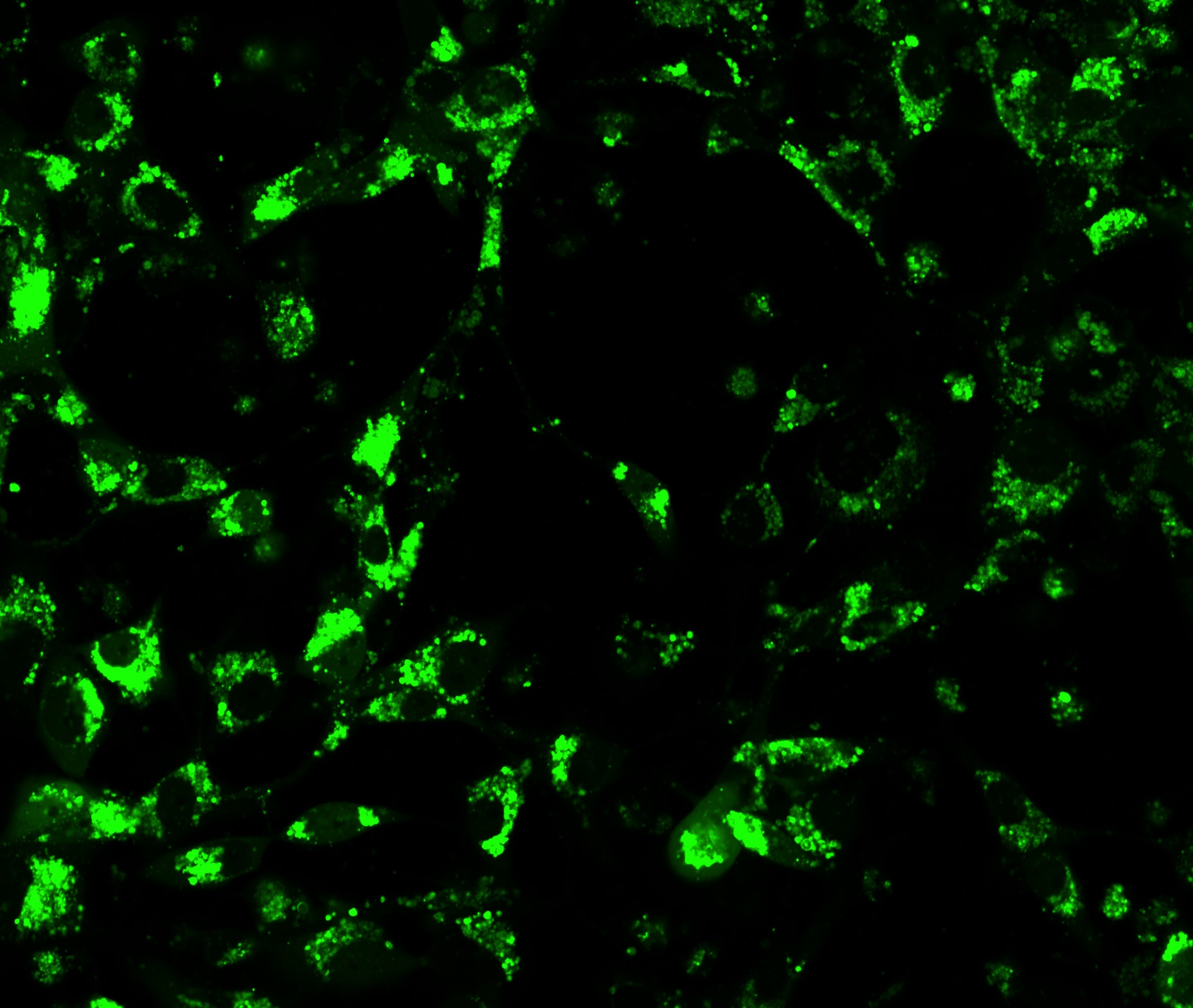

Supplement: Supplementary file 7 [file DataSheet5.ZIP › original data of sensGFP-stubRFP-LC3/FIG.8/CQ/GFP.jpg]

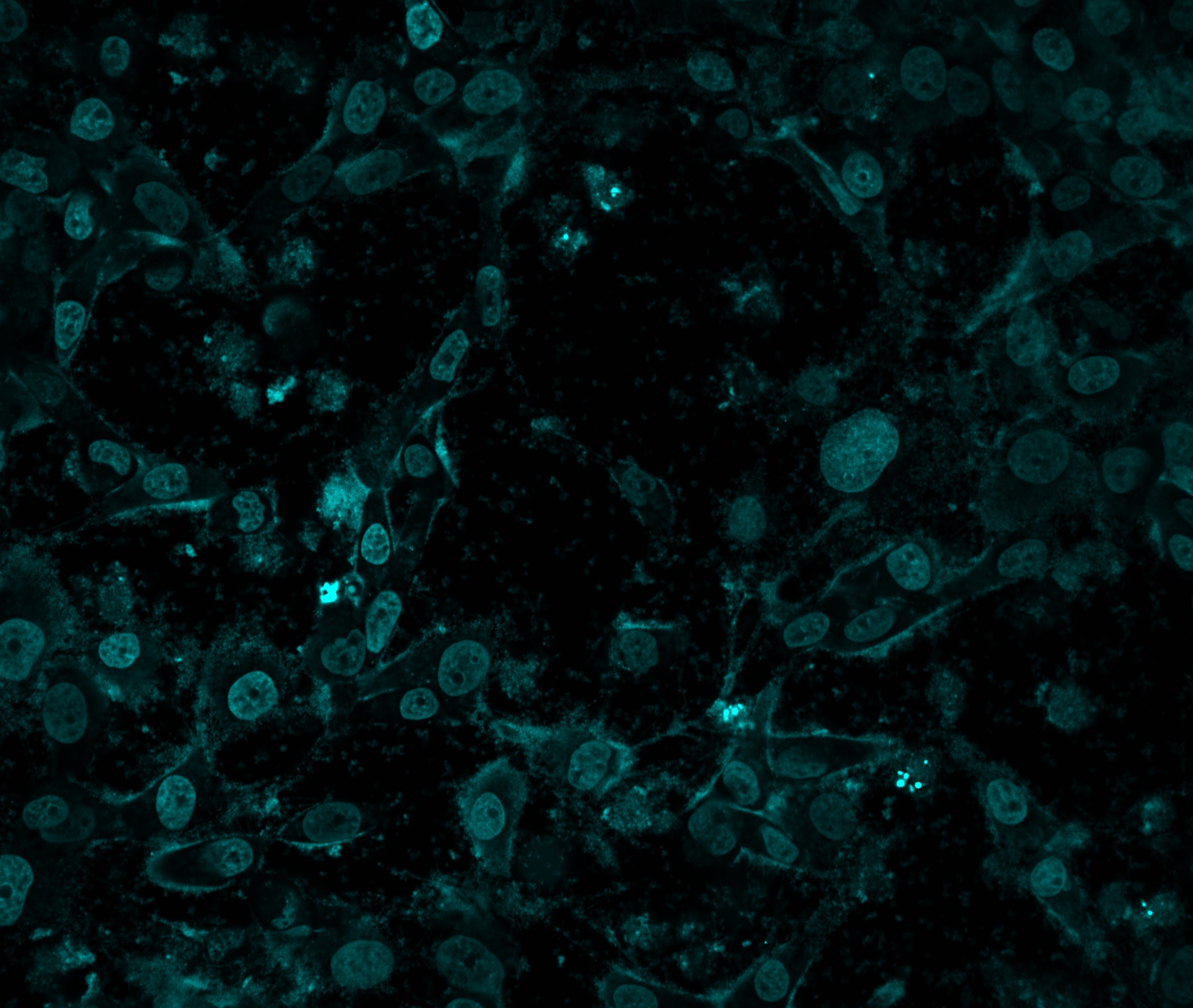

Supplement: Supplementary file 7 [file DataSheet5.ZIP › original data of sensGFP-stubRFP-LC3/FIG.8/CQ/Hoechst.jpg]

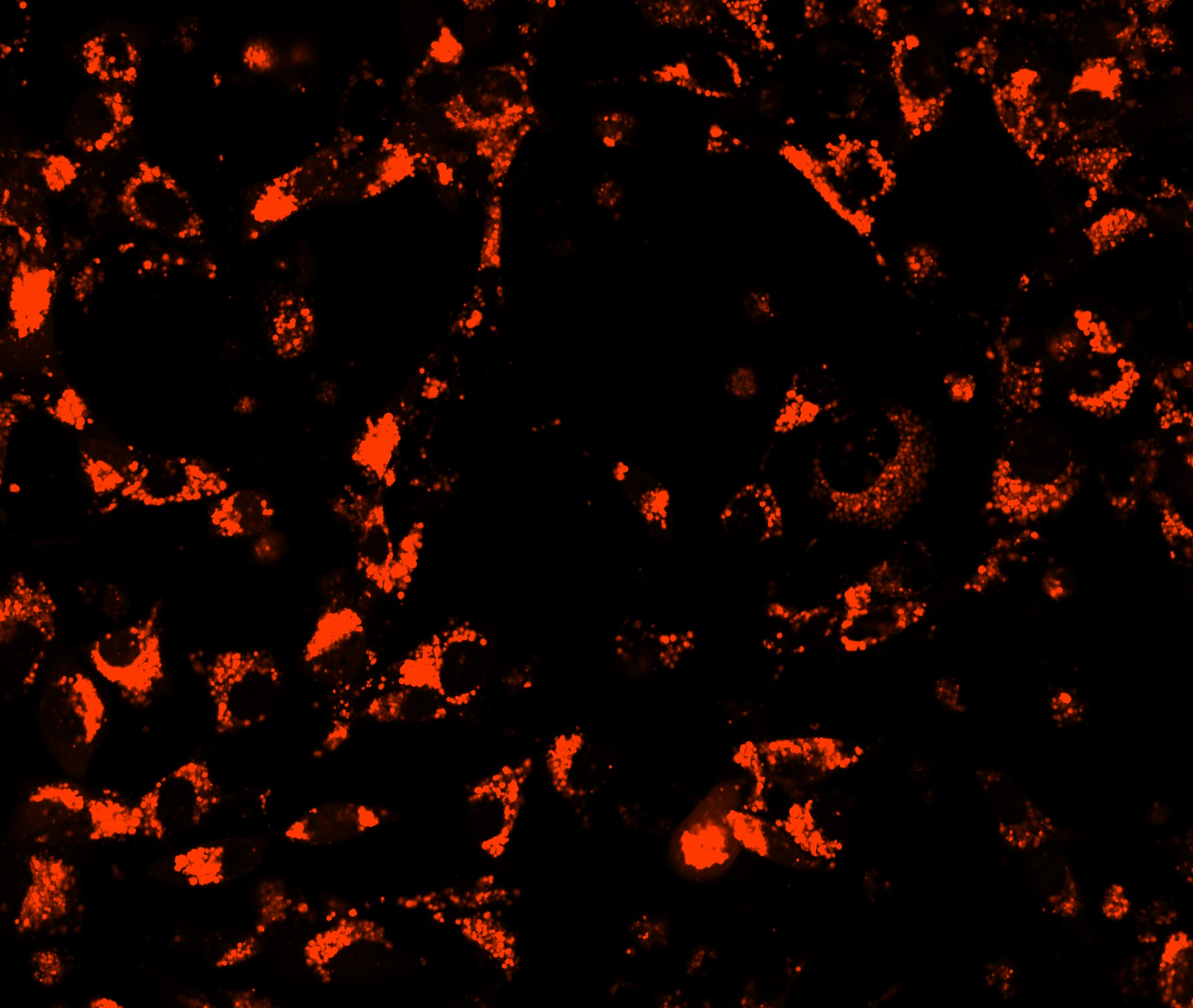

Supplement: Supplementary file 7 [file DataSheet5.ZIP › original data of sensGFP-stubRFP-LC3/FIG.8/CQ/RFP.jpg]

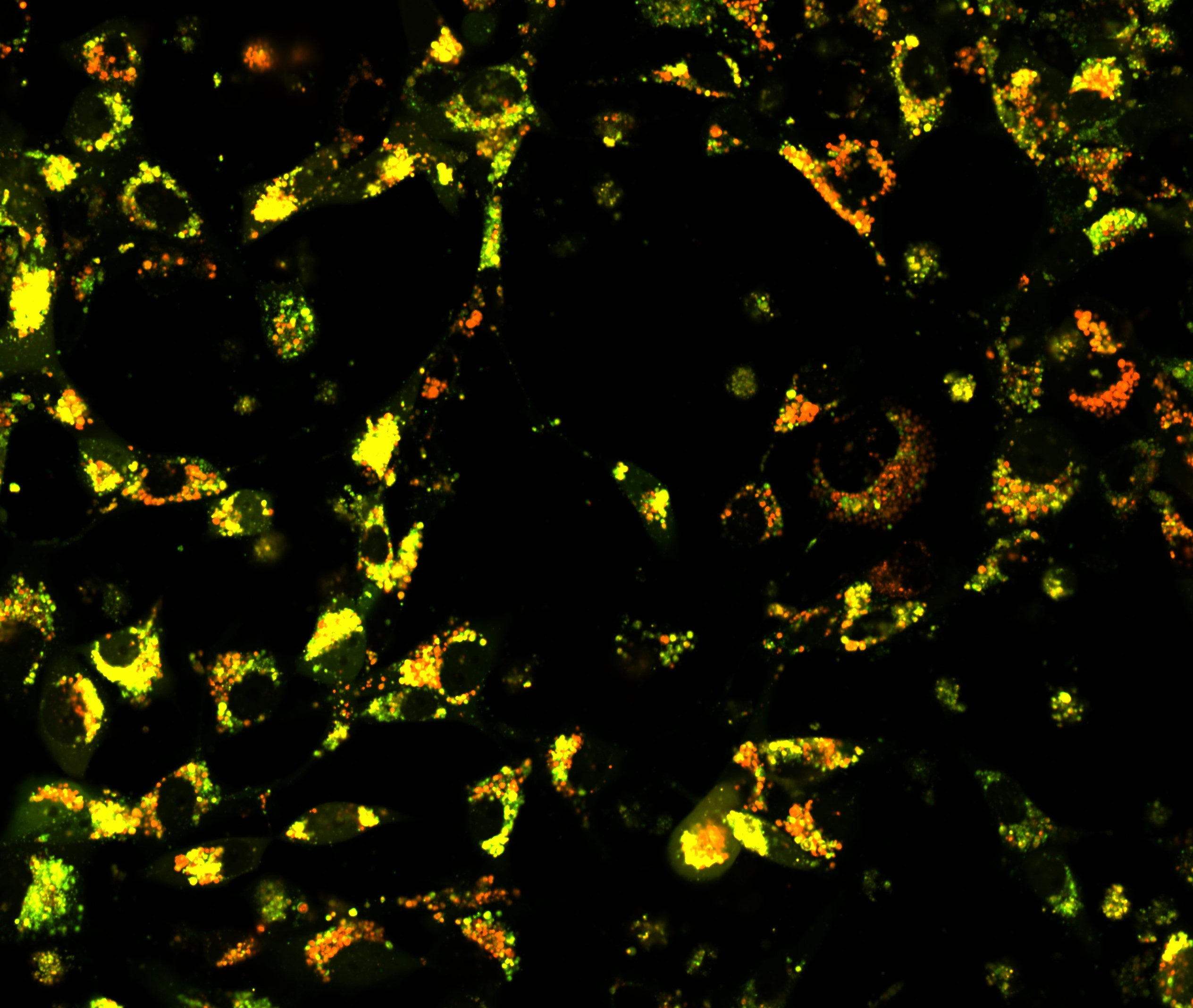

Supplement: Supplementary file 7 [file DataSheet5.ZIP › original data of sensGFP-stubRFP-LC3/FIG.8/CQ/RFP+GFP.jpg]

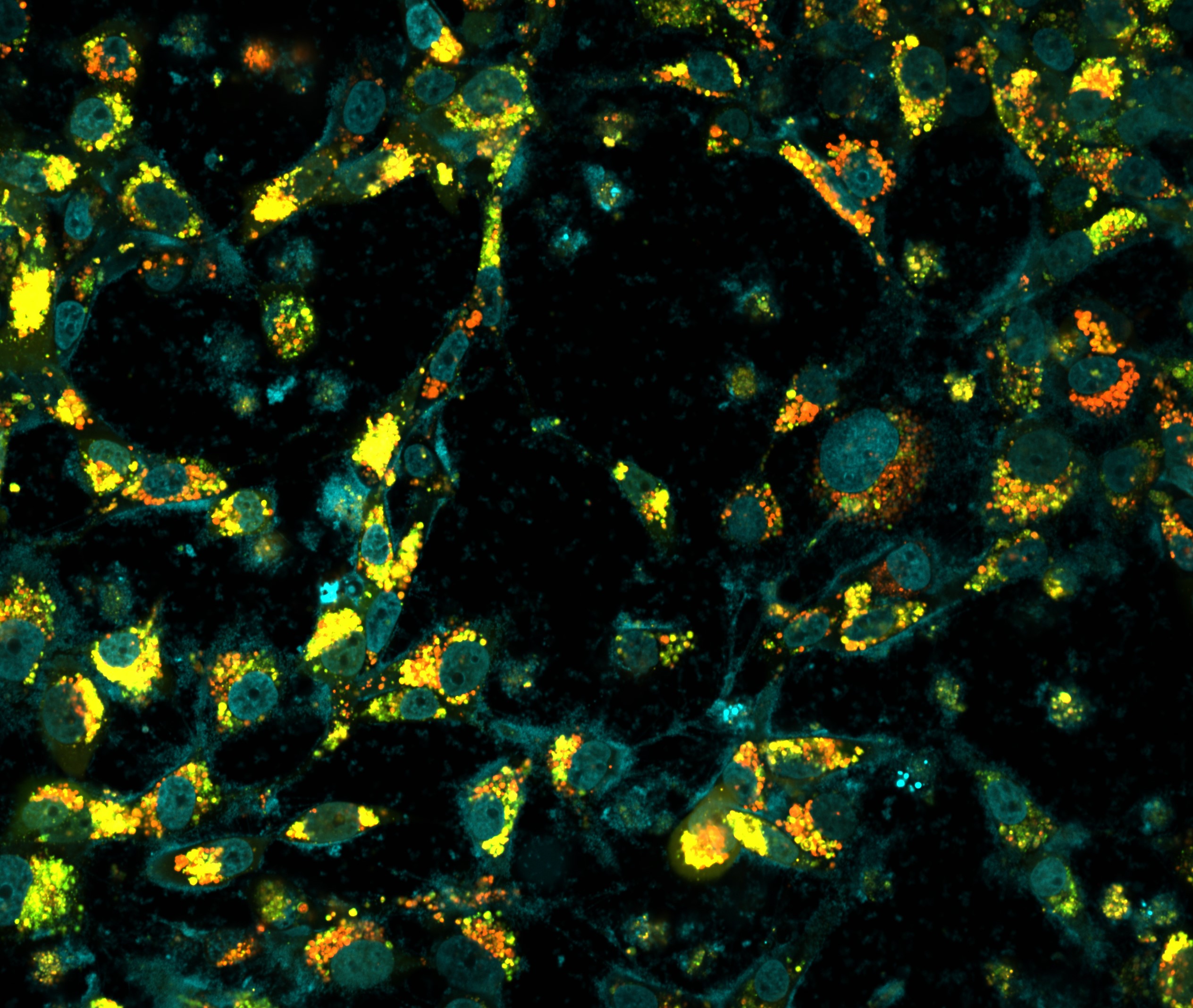

Supplement: Supplementary file 7 [file DataSheet5.ZIP › original data of sensGFP-stubRFP-LC3/FIG.8/CQ/RFP+GFP+Hoechst.jpg]
